# Capturing sources of health system legitimacy in fragmented conflict zones under different governance models: a case study of northwest Syria

**DOI:** 10.1186/s12992-024-01074-4

**Published:** 2024-10-03

**Authors:** Munzer Alkhalil, Rim Turkmani, Mazen Gharibah, Preeti Patel, Zaki Mehchy

**Affiliations:** 1 LSE IDEAS, Conflict and Civicness Research Group (CCRG), London School of Economics and Political Science, London, UK; 2 Research for Health System Strengthening in Northern Syria (R4HSSS), Union for Medical and Relief Organizations, Gaziantep, Turkey; 3 Research for Health System Strengthening in Northern Syria (R4HSSS), The Centre for Conflict & Health Research (CCHR), King’s College London, London, UK

**Keywords:** Health system, Governance, Legitimacy index, Top-down, Bottom-up, Legality, Justification, Consent, Performance

## Abstract

**Introduction:**

Legitimacy and trust are crucial for resilient health systems in fragmented conflict zones. This study evaluates the legitimacy of health systems in northwest Syria under different governance models.

**Methods:**

Using a deductive and inductive mixed-methods approach, the research team developed a framework with an index, 4 sub-indices and 18 indicators to assess the legitimacy of health systems using different governance models – top-down, bottom-up, and hybrid – in the context of the response to the earthquake that hit Syria in February 2023. The study includes surveys, workshops, stakeholder consultations, and an expert panel conducted in northwest Syria and online.

**Results:**

The findings indicate that bottom-up health governance model is perceived as the most legitimate, followed by the mixed model, while top-down model is perceived as the least legitimate. This preference is measured across all legitimacy source sub-indices, including legality, justification, consent and performance and across the overall Health System Legitimacy Index (HSLI). However, the hybrid governance approach showed limited superiority at two indicator levels regarding long-term health system response.

**Conclusion:**

This study highlights the importance of considering the legitimacy of the health system in fragmented conflict zones. It helps explain the effectiveness of the bottom-up approach and community-based governance in enhancing trust, cooperative behaviour, health interventions and achieving sustainability. Additionally, the study highlighted the role of legitimate health systems in practising civic virtue and promoting social justice, thus contributing to peace-building efforts. These insights are crucial for policymakers and development donors to strengthen health systems in challenging contexts.

**Supplementary Information:**

The online version contains supplementary material available at 10.1186/s12992-024-01074-4.

## Key messages

- What is already known on this topic
  Health systems in fragmented conflict zones face significant challenges, including issues of governance, effectiveness and securing compliance. Studies have shown that health governance evaluations in these settings are often limited in scope, favouring simplified designs focusing on health governance principles and lacking comprehensive approaches. Studies focusing on health systems legitimacy are scarce, indicating a clear gap in existing research.
- What this study adds
  This study added a new dimension to health governance evaluation frameworks that goes beyond the principle dimensions to include legitimacy sources. In addition, the study created a framework to evaluate health systems’ legitimacy and a Health System Legitimacy Index (HSLI), which is useful for fragmented conflict zones.
- How this study might affect research, practice or policy
  The research team develops a comprehensive framework for evaluating health governance in conflict areas that includes legitimacy as one of the key dimensions. This paper contributes directly to the building of that framework. Moreover, we argue that using our new approach to capture sources of trust and legitimacy reveals a more granular perspective that is required for optimising health interventions. It also reveals important variations between the different models of governance that could guide the design of the health systems in conflict zones so that they can generate cooperative behaviour required for enhancing efficiency and sustainability. In addition, policymakers, including development donors, can use this study to boost the ability of health systems in conflict areas to exercise civic virtue and enhance social justice and thus their ability to play a peace-generating role.

**Supplementary Information:**

The online version contains supplementary material available at 10.1186/s12992-024-01074-4.

## Introduction

In the past two decades, the legitimacy of healthcare systems has garnered more attention due to its correlation with enhanced governance and voluntary compliance [1–3]. Additionally, legitimacy and trust are recognised as crucial elements in strengthening the resilience and efficiency of health systems, particularly in low- and middle-income countries [4, 5] and strengthening the humanitarian localisation agenda [6].

Governing and service institutions generally seek to achieve public compliance through voluntary compliance, legal authority, and coercive power. Voluntary compliance refers to the public’s willingness to adhere to rules and directives issued by an institution without the need for coercion. In contrast, coercive power involves the institution’s ability to enforce compliance using force, threats, or sanctions. It is widely accepted in the literature that the more an institution is perceived as legitimate by the public, the less it needs to rely on coercion to secure compliance [7–10]. This perception significantly influences how the public and individuals view the authority and actions of the institution, affecting their willingness to comply with its rules and directives [9, 10].

Securing voluntary compliance is particularly important for health institutions because they rely on the cooperation of the recipients of their services and do not typically exercise coercive power to ensure this cooperative behaviour [3, 9].

The literature on the legitimacy of public health authorities, however, remains predominantly concerned with the legitimacy of formal state health institutions [1–3, 9] and international health organisations [11]. There is also a greater focus on the question of trust in health institutions rather than the broader legitimacy of these institutions [3–6].

The legitimacy of governance institutions and actors is found to be mainly based on trust [12] [13]. Academic literature supports the interdependence of trust and legitimacy through a positive feedback loop where each reinforces the other [14, 15]. Trust forms the foundation for legitimacy [7, 16]; when an institution is perceived as legitimate by the public, it is more likely to be trusted. This is because legitimacy signals adherence to societal norms and values, fostering greater trust.

However, understanding the nuances of trust in public health authorities or systems alone does not fully address other sources of health system legitimacy, such as the perception of the legality of these institutions and their conformity to local beliefs and values.

Responding to health emergencies during times of crisis brings the questions of legitimacy and trust to the fore, which is why these issues attracted particular attention during and after the COVID-19 pandemic [11, 17]. In such a context, trust has been found to be vital for adherence to prevention measures [18]. A literature review on the role of trust in COVID-19 vaccine acceptance found that institutional trust is key in addressing vaccine hesitancy [19].

A 2021 cross-country comparative study revealed why maintaining trust in public health institutions and providers is a crucial task in fragile contexts. By investigating seven areas in three conflict-affected countries, the study concluded that trust in healthcare systems and their wider legitimacy are key drivers of seeking health services in such contexts [20]. Yet, it is particularly in the context of fragile and fragmented conflict zones that the nuances of legitimacy are least explored in the literature on legitimacy in general [21–23].

Institutions and the systems of governance in fragmented conflict zones have unique characteristics that make it difficult to use the same methods of assessing what makes them legitimate as those used in more stable contexts [21, 24]. Three main issues call for attention in such contexts.

First, there is the issue of governance and its correlation with legitimacy. In fragmented conflict zones, the centrally coordinated formal system of governance disintegrates. New and diverse forms of de facto systems emerge out of the chaos within different zones to respond to people’s health needs. Because international health organisations and donor countries are often part of the health response in these contexts, they play a direct and indirect role in the formation of new health systems and the provision of health services. With this, they bring in new norms, such as an emphasis on transparency and impartiality, and thus affect the expectations of the citizens. In some areas, the emerging systems are strongly influenced and controlled by an external state. Traditional norms also rise to prominence in conflict settings and affect the way new systems are built. As such, the emerging health systems in such contexts are a mix of varying degrees of the old formal system, new bottom-up systems and global humanitarian system. In some cases, interventions from an external state could also impose a top-down component on the emerging system. This mix produces a spectrum of forms of governance that has the predominantly bottom-up system on one end and the predominantly top-down system on the other end.

The literature exploring the correlation between health systems and legitimacy typically investigate the link between the provision of health services by a state-run institution and the legitimacy of that state [1–3]. But in the context of fragmented conflict zones, the emerging health system is either not linked to a formal state governing structure or weakly linked to it. As such, the legitimacy of the health system itself becomes the focus of legitimacy inquiry. The link between the different forms of health system governance that emerge in conflict zones and the perceived legitimacy of these systems is not explored in the literature.

Second, there is the linked central issue of securing compliance. In a stable state setting, we expect an alignment between legal means of securing compliance and the means based on consent and coercion. Health institutions in a functioning state could rely, for example, on the law of the land and its enforcement to ensure vaccine uptake and adherence to protective measures during a pandemic. In the turmoil of conflict zones, such alignment rarely exists, and emerging health systems tend to distance themselves from political and armed actors in areas to protect their impartiality and independence. As such, voluntary compliance becomes the cornerstone for delivering health services and protecting health workers.

Third, there is the issue of representation, which is tightly linked to trust. In a stable state setting, the formal institutions represent and communicate people’s health needs and interests, especially with international humanitarian organisations. In conflict zones, with the collapse of state authority and the absence of democratic mechanisms for representation, the de facto health systems often play a representative role, especially with international organisations that typically play an important role in providing health services in conflicts. As such, the capacity of those in the health system to understand and communicate the needs of the people, to create a system of accountability, and their ability to secure the trust of the people to represent them in important decision-making arenas becomes very important. Evidence shows that in humanitarian emergencies, despite the rhetoric about accountability to people and their actual priorities in such settings, practice often shows little respect for these issues [6]**.** Recent literature highlights the importance of decisions by international humanitarian organisations having legitimacy by being attuned to people’s needs and backed by consultations with them [25]. Capturing the views of affected people in a humanitarian crisis has been shown to improves the efficiency of humanitarian response [26].

Therefore, understanding the nuances of trust and legitimacy in such complex environments could strongly impact the design of more effective and acceptable health interventions [25–29]. Legitimate and effective service sectors in conflict zones, notably health and education, have impacts beyond their respective sectors. They can significantly contribute to track two (civil society track) in political negotiations, peacebuilding activities, the exercise of civic virtue, the promotion of social justice, the sustainability of services, and play a critical role in the conflict-to-recovery transition [30].

Although the question of how to understand and measure trust of healthcare systems has attracted some attention [3, 12, 20], the question of the wider legitimacy of healthcare systems has received considerably less attention in the literature in general, and in particular in the context of fragmented conflict zones. The only example we identified is a framework for assessing the legitimacy and practices of discursive (de)legitimation of global governance institutions (GGIs) developed by Hai Yang [11]. By assessing the legitimacy and discursive (de)legitimation of a GGI, Yang’s framework can capture the finer grains of the empirical sources of legitimacy of a GGI, such as purpose irreplaceability, legality, inclusiveness, transparency, accountability, integrity, impartiality, problem-solving effectiveness and executive leadership [11]. But the framework was developed to examine specifically the legitimacy of international health institutions and is short of capturing the nuances of the sources of legitimacy of national and local health institutions and thus health systems.

Our research aims to contribute conceptually and empirically to understanding the sources of legitimacy of healthcare systems in fragmented conflict zones. This will be achieved by developing a new framework in the methodology section. We will utilise this framework to assess citizens' perceptions of the multiple sources of legitimacy of healthcare systems under different governance models (top-down, hybrid, and bottom-up), using a northwest Syria (NWS) case study.

Our hypothesis is that the people’s perception of the legitimacy of public health systems varies across different de facto governance models. We operationalise our framework to design a questionnaire and conduct a survey to capture the sources of legitimacy of the health system in three different areas in NWS. These areas are characterised by three different models of de facto health governance: the top-down, hybrid, and bottom-up models (see Table 1 for definitions). The survey was carried out six months after the earthquake that hit Syria and Turkey on 6 February 2023. The health system played a pivotal role as a frontline responder immediately after the earthquake and in addressing multiple health issues in the period following, both on the ground and through representing the local community in the media and in front of international organisations to communicate needs and coordinate appeals [31].

**Table 1:** Key Definitions

Legitimacy of a public authority	The legitimacy of a public authority is the recognised and accepted right to exert influence and act, both by political elites and the general public, seen as lawful and justified in society and national politics [32–36]
Trust in public authorities	Trust in public authorities refers to the public's confidence and satisfaction in these institutions and their leaders to act in the best interests of society [13, 18, 37]
Public Authority	Any authority beyond the immediate family that asks for voluntary obedience from the people [38]
Conflict-zone	Areas experiencing armed conflict, post-conflict fragility, weak governance, poor security, and systematic violations of international law, including human rights [39]
Health System	“All organizations, people and actions whose primary intent is to promote, restore and maintain health” [40]
Top-down governance	Hierarchical governance involves the direct application of state authority to target populations. It is used to enforce rules or standards of behaviour on other actors, supported by incentives and penalties, to achieve collective objectives [41]
Bottom-up governance	An approach that enables local communities and stakeholders to express views and help determine the development direction for their area in line with their opinions, expectations and plans [42]
Hybrid governance	Refers to shared decision-making processes and responsibilities between higher-level governmental bodies and local or community-based organisations. It aims to balance standardisation and adaptability to ensure that health services are effective and meet local needs and expectations

As such, the period after the earthquake was a real test of the efficiency and responsiveness of the health system, and it was also a period when most people had direct experiences with the health system, thus forming views of both its performance and its capacity to represent them. Table 1 outlines key concepts utilised in this paper.

## Contextual background

The Syrian conflict started in 2011 and resulted in more than 874,000 deaths and more than 13 million refugees and internally displaced people (IDPs) [43]. It led to the political fragmentation of the country with different areas of control [44]. The NWS, (focus of this study), came under the military control of various armed actors since 2015 after the central government in Damascus lost control [45]. The withdrawal of the state led to a governance vacuum which led to the development of various de facto governance structures. By 2017 the NWS itself was split into two main areas under the control of two different de facto governments, with internal crossings between them. These are Idilb, governed by the Syrian Salvation Government (SSG), which is linked to Hayat Tahrir al-Sham, a military group classified as a terrorist organization [46, 47], and northern Aleppo which is governed by the Syrian Interim Government (SIG), which is derived from the National Coalition for Revolutionary and Opposition Forces with strong influence from Turkey [48].

But despite the chaos of governance in the NWS and the affiliation of governance bodies with armed and political actors, the health sector in Idlib maintained relative independence from armed and political actors [49]. This is primarily due to the way this sector emerged in this area, depending on a bottom-up institution driven by local medical staff who were determined to keep the sector independent to protect it from the political and armed competition [50]. To regulate the health sector, the leading figures in the health sector founded the Idlib Health Directorate (IHD) in 2013. To maintain the independence of IHD from the controlling authorities, it was designed so that it derives its legitimacy from the grassroots community or bottom-up. IHD would have a general assembly, which includes representatives of more than two hundred health facilities in the governorate [51]. The General Assembly elects a board of trustees, which in turn elects the head of IHD [52]. Although the SSG formed its own Ministry of Health (MoH) in Idlib in 2017, its role remained marginal [53]. Between 2017 and 2019, MoH/SSG had no more than three employees, while IHD had around 800 employees in the same period, supported by different donors. Although recently, MoH/SSG enhanced its capacity by increasing its staff and supporting a few health facilities in the area, more than 90% of public health facilities are supported by non-governmental institutions (NGOs) and are members of the General Assembly of IHD. This meant that the health sector in Idlib remained mainly a bottom-up sector managed by the IHD-NGO allies [31, 50, 51].

In northern Aleppo, the Aleppo Health Directorate (AHD) emerged in 2013 from local health staff and maintained independence until 2014, when it joined the SIG umbrella [54], representing a different governance approach than IHD; it did not establish an election process or have a board of trustees, but rather the MoH at SIG names the head of AHD. The influence of the MoH of the SIG became clear in 2014 when the SIG received a $68 million grant from Qatar [55]. However, its influence became marginal after that due to the lack of capacity, legitimacy and resources [47, 55, 56]. Its role was almost limited to hiring the head of AHD and marginal health activities in northern Aleppo. This weak role of the SIG keeps a balance in power between AHD and MoH/SIG.

After the Turkish military intervention in northern Aleppo in 2016 [57], Turkey played a key role in the health sector in the area. It built many health facilities and managed them directly through health directorates in southern Turkey. In addition, Turkey established a different governance structure in the area, including health offices linked to local councils. In parts of northern Aleppo, Turkey took control of the management of the health sector and even applied the Turkish health system in these areas (such as Afrin, Al-Bab, Ar-Ra’ee, A’zaz and Mare). This resulted in a top-down managed health sector.

In other parts of northern Aleppo, the Turkish authorities did not assume full control of the health sector and left it to be managed by AHD, NGOs, and the MoH/SIG, such as Akhtarin and Raju. This arrangement resulted in a hybrid model combining aspects of bottom-up governance, represented by the supervision of Turkish authorities and MoH/SIG, and top-down governance, represented by IHD and NGOs.

The strength and resilience of the health sector in NWS were repeatedly tested during the multiple crises it faced, particularly during the coronavirus pandemic and the February 2023 earthquake. Over 51,000 people died in both Turkey and Syria, with around 4540 in NWS [58, 59]. In addition to the health directorates in Aleppo and Idlib, other actors played a role in response to the earthquake, mainly the NGOs, INGOs, the Health Cluster in Gaziantep, which is led by the World Health Organisation (WHO) and other united nations’ agencies (UN), including United Nations Development Programme, International Organization for Migration, and United Nations International Children’s Emergency Fund.

## Methodology

### Methodology processes

Our mixed empirical methodology consists of seven main activities: 1) a scoping literature review; 2) using grounded theory to draft the conceptual framework; 3) three workshops to develop the framework and a questionnaire, validate the health governance models criteria and discuss the results; 4) two training sessions for data collecting team; 5) a field survey; 6) thirteen stakeholder consultations to identify the governance models and legitimacy level; and 7) an expert panel to validate the weight value for sub-indices and indicators and to discuss the initial findings based on the given weight.

#### Framework development processes

To develop the conceptual framework, we began with the types and subtypes of institutional legitimacy in complex contexts identified in the Turkmani framework [21]. We then identified eighteen legitimacy indicators (principles) specific to health systems in conflict zones, in line with the grounded theory approach, based on more than a decade of the first author’s experience in health governance amidst complex conflicts. This process was further refined through discussions among all the authors. The last step involved scoping the literature to further refine and support all legitimacy sources and indicators.

Then, we held two one-day workshops for the research team, including an external governance expert at London School of Economics in London, between April and June 2023 to develop and adapt the conceptual framework and to design related questions for the survey and to validate the health governance models criteria.

#### Training and preparation for fieldwork

In preparation for the fieldwork activities, we organised two online training sessions for 12 field enumerators based in NWS, except one based in Gaziantep/Turkey in June 2023, to elucidate the survey’s objectives and provide detailed clarification for each question within the questionnaire. Additionally, we compiled a comprehensive local researcher guide in Arabic that offers thorough explanations and examples for each question, aiming to minimise subjectivity in question comprehension by both enumerators and surveyed individuals. The lead author maintained direct supervision over field activities regularly, as well as the data entry process. The fieldwork team utilised Excel for entering data and following a thorough data cleaning and verification process by the research team, the data was imported into Stata 17.0 for further analysis.

#### Survey execution

We conducted the survey between July and September 2023 in 35 sub-districts in NWS, 17 of which are in the Aleppo governorate and the remaining in Idlib. The sample was initially drawn from the overall population of 2.2 million individuals aged 18 years or older, residing in NWS, as per data from the UN Humanitarian Needs Assessment Programme for Syria [60]. With a sample size of 1089 persons, representative of the population with a 95% confidence interval and 3% margin of error, the selection process ensured statistical validity.

Subsequently, the sample was allocated across the 35 sub-districts based on their respective population sizes. This two-step approach aimed to guarantee representation across the entire region while maintaining proportional representation within each sub-district. Furthermore, within each sub-district, individuals were randomly selected, with deliberate efforts to achieve gender balance in the sample. Data analysis indicates that the sample has a relatively well-balanced gender and age distribution. Among the respondents, 45% are female, and 36% are between the ages of 18 and 33. The employment rate within the sample is 44%, with 28% of the employed individuals being female. Nearly half of the employed individuals work in the service sector. Regarding the geographical distribution of the sample, approximately 65% are from Idlib, and 35% are from Aleppo (More demographic information, including sex and age, as well as distribution by governorate, sub-district, and governance model, are available in Appendix 2).

#### Identification of governance models

Since we aimed to test our framework in areas that we identified as governed under three different governance models, we identified from the literature the criteria required to classify health governance models. Then, we validated the criteria in two workshops for the research team with an external health governance expert. The results are summarised in Table 2.

**Table 2:** Health governance model criteria

Criteria	Top-Down Approach	Bottom-Up Approach	Hybrid Control Approach
Hierarchical Structure	Clear, top-to-bottom reporting and control mechanisms [61]	Decentralised structure [62]	Elements of both hierarchical and decentralised control [63]
Centrality of decision making and level of participatory approach	Policies and decisions are made primarily by centralised authority with little or no participation of local community [64]	Policies and decision-making are heavily influenced by local communities or grassroots organisations [42, 65]	Shared Decision-making: Policies are made through collaboration between centralised and local entities [61]
Standardisation	Uniform health practices and services across all areas [62]	Adaptable health practices and services that are tailored to local needs and preferences [66]	Partial Standardization: Some uniformity in health practices, but also room for localised adjustments

The allocated sample size for each governance model is the sum of the sample sizes of the sub-districts under the same governance model. Accordingly, the sample distribution by the three governance models is as follows: 17.7% top-down, 11.7% hybrid, and 70.6% bottom-up model.

#### Stakeholder consultations

To identify the governance model each surveyed sub-district followed, we conducted four rounds of virtual stakeholder consultations via Zoom involving representatives from quasi-government institutions and NGOs. We followed that with short phone consultations with four stakeholders’ representatives from NGOs and WHO. The aim was to identify the governance models within each sub-district. We explained the criteria outlined in Table 2 and asked the local consultants to group the subdistricts under the three mentioned governance models. We conducted another consultation with Health Information System Unit (HISU) to validate the primary results of the health governance models.

To determine the level of legitimacy in this context, we need to assess, whether it is at the public health authorities or health system level. We then conducted four additional brief phone consultations with representatives from NGOs and WHO. All consultations were done with people working for the health sector in NWS and were based in Syria or Turkey. All the consultations were done between July 2023 and May 2024.

#### Validation

We held a workshop in October 2023 for the research team in London to discuss the results. This was followed by organising a virtual expert panel in December 2023, which included five local experts and medical staff, to validate the given weight of sub-indices and indicators and our descriptive analysis results, aiming to establish a robust analytical narrative that complements the quantitative findings.

### Developing the conceptual framework

Our starting point in developing a framework to capture the perception of the legitimacy of healthcare systems in fragmented conflict zones is a framework developed by Turkmani to capture the perceived legitimacy of institutions in general in complex settings, including fragmented conflict zones [21]. The framework aims to assess legitimacy as believed by citizens rather than as claimed by authorities. The framework starts from four subtypes of legitimacy: views of legality (which is divided into internal and external legality), views of justification, acts of consent, and views of performance (also referred to in the literature as instrumental legitimacy). The definitions of these subtypes are outlined in Table 3.

**Table 3:** Framework for evaluating the legitimacy of health systems in fragmented conflict zones

Legitimacy Sources	Definitions	Questionnaire indicators	Constitutive or substitutive
Views of legality	Whether the authority gets and exercises political power in accordance with citizens’ views on laws, rules, and norms [67]	1- Coordination and cooperation among responding agencies [68, 69]	Constitutive
		2- Cooperation of responding bodies with local authorities [70]	Constitutive
		3- Involvement of the local community in the decision-making process [71, 72]	Constitutive
		4- Community accountability [73, 74]	Constitutive
		5- The legal foundation of the responding bodies [75]	Constitutive
		6- Ability to coordinate with international donors and to secure services and support from them [76]	Constitutive
views of justification	Agreement with shared principles, beliefs, values and ideas [67]	1- Transparency [73, 77, 78]	Constitutive
		2- Equity [9, 35, 79, 80]	Constitutive
		3- Incorruptness [35, 81]	Constitutive
		4- Understanding people’s needs [82]	Constitutive
		5- Impartiality [81, 83]	Constitutive
		6- Respect for community customs and traditions [84]	Constitutive
acts of consent	When someone gives permission for something to be done or acknowledges the authority of someone or an entity [85]	1- People’s compliance with health advice and instructions issued by responding agencies [81]	Constitutive
		2- Delegation of the local community to the responding bodies to represent community health interests and provide services [86]	Constitutive
Views of performance (Instrumental legitimacy)	Assessing an organisation’s capability to achieve its objectives effectively and efficiently while considering aligning its actions with societal expectations [35, 78, 87–89]	1- Rapid response [90]	Substitutive
		2- Quality of health services [91]	Substitutive
		3- Availability of health services [92]	Substitutive
		4- Effective health services (reliability) [35, 91]	Substitutive

To adapt the Turkmani framework to the health system we used deductive and inductive approaches to come up with a set of constitutive and substitutive indicators for each sub-type of legitimacy using grounded theory relying on the first author’s lived experience in the health sector in NWS. We followed the methodology developed by Bollen and Lennox (1991) to derive specific ‘substitutive and constitutive indicators’ for each of the four sources [93]. Constitutive indicators are direct indicators that constitute the essence of what is being measured [93–95], and are used to measure people’s perception of procedure or input legitimacy sources. While substitutive indicators are used as substitutes for direct measurement when constitutive indicators are hard to quantify or observe. They do not directly measure the concept but are close enough to provide a useful proxy to measure people’s perception of performance or output legitimacy sources [93–95].

The resulting 18 indicators we derived are summarised in Table 3 together with the literature that we relied on to justify each indicator. Our main criterion in selecting these indicators is that the indicator should be significant and justifiably related to one of the legitimacy sources. Table 3 shows that these indicators were divided into four sub-indices (sub-types) of the legitimacy index. We also ensured that each one of the four sub-indices of legitimacy index is covered by several indicators that are suitable to capture the specifics of this sub-index within a fragmented conflict setting. The indicators for the view of performance were also chosen to assess the tangible performance of health system in particular. For the indicators of the act of consent, we chose to look not only for people’s willingness to voluntarily comply with the instructions of the health authority, but also to investigate people’s willingness to delegate the health authorities to represent them and convey their needs to international bodies, such as donors and the WHO. This corresponds to the question of representation raised in the introduction. The indicators for the view of legality were also chosen following the Turkmani approach in compensating for the absence of formal legality by examining the procedures and performances that produce legality and social accountability and we tailored this to the context of health systems. The indicators for the view of justification to answer the issues raised in the introduction in regard to the expectations of legitimacy audience in conflict setting where institutions are not only expected to conform to local customs and understand local needs but also match the norms invoked by international organisations.

We also relied on two one day workshops organised include the research team and an external governance expert to validate these indicators.

We discussed the level of legitimacy in this context, whether health system legitimacy or public health authorities’ legitimacy. Given that we have a multipolar health system with no dominant government and thus multilateral health governance, and we used two types of indicators: 1) constitutive indicators, which are linked more to the public health authorities’ legitimacy and 2) substitutive indicators, which are linked more to the health system level. We considered two levels: the first one is the ‘collective legitimacy of coopetition public health authorities’, and the second level is ‘health system legitimacy.’ After a discussion within the research team and four consultations with local experts, we agreed that the collective legitimacy in this context expresses the health system legitimacy, which includes the public health authorities by default, supported by the two types of indicators.

We operationalised this framework to design a questionnaire with a question for each indicator where people are asked to rate their perception on a five-point scale, ranging from 1 (very poor) to 5 (very good) (Appendix 1).

Finally, this framework is not a model for how health systems should work or behave in crises because all results are based on people’s perspective regarding the mentioned indicators (principles) rather than actual evaluation to these indicators within the health system. These perspectives are influenced by many reasons including people’s interest and principles’ visibility. This framework is to capture and evaluate legitimacy sources of health system in conflict areas and thus enhance the legitimacy of these systems and health governance.

### Developing the health system legitimacy index (HSLI)

With the aim of developing an index for health system legitimacy, we constructed sub-indices for the four legitimacy sources to construct the Health System Legitimacy Index (HSLI) using a simple weighted arithmetic average formula:

HSLI = $$\sum_{j=1}^{n}\sum_{i=1}^{d}{w}_{i}{x}_{ji}$$ , j = 1,2,…n.

Where ${x}_{ji}$ represents the score of the indicator i for observation (individual) j, ${w}_{i}$ denotes the weight assigned to i indicator. The weights satisfy $\sum_{i=1}^{d}{w}_{i}$ = 1 and 0 < ${w}_{i}$  < 1. In constructing the HSLI, four sub-indices are utilized, resulting in d being equal to 4. HSLI value ranges between 1 and 5. It is calculated as the sum of the four weighted sub-indices for each observation (individual) j.

Following expert discussions and consultations with local practitioners, we assigned equal importance to each indicator within the four composite sub-indices. This method also aligns with Bruce Gilley’s ‘unweighted score’ concept [93]. We also applied Principal Component Analysis (PCA) to check for the relative importance for each of the four selected indicators. The results show that indicators loadings on the first principal component range between 0.47 and 0.51, reflecting the correctness of using equal weights in the index. Thus, *wi* equals 0.25 for each sub-indices in the above equation.

We applied the Cronbach’s alpha coefficient (c-alpha) to assess the internal consistency of the indicators used in developing HSLI (Appendix 2). This statistical measure is particularly relevant when creating a composite index as a scale [96], which aligns with our approach. The computed c-alpha for HSLI exceeds 0.70, indicating a satisfactory level of internal consistency within this set of indicators [97]. We also applied c-alpha analysis on the four sub-indices (Appendix 2), the results show that the lowest scale reliability coefficient is between the indicators constructing the Act of Consent sub-index at 0.58, and the highest is between the indicators of View of Justification sub-index at 0.83.

## Results and discussion

### Health governance approaches in NWS

**Fig. 1:**
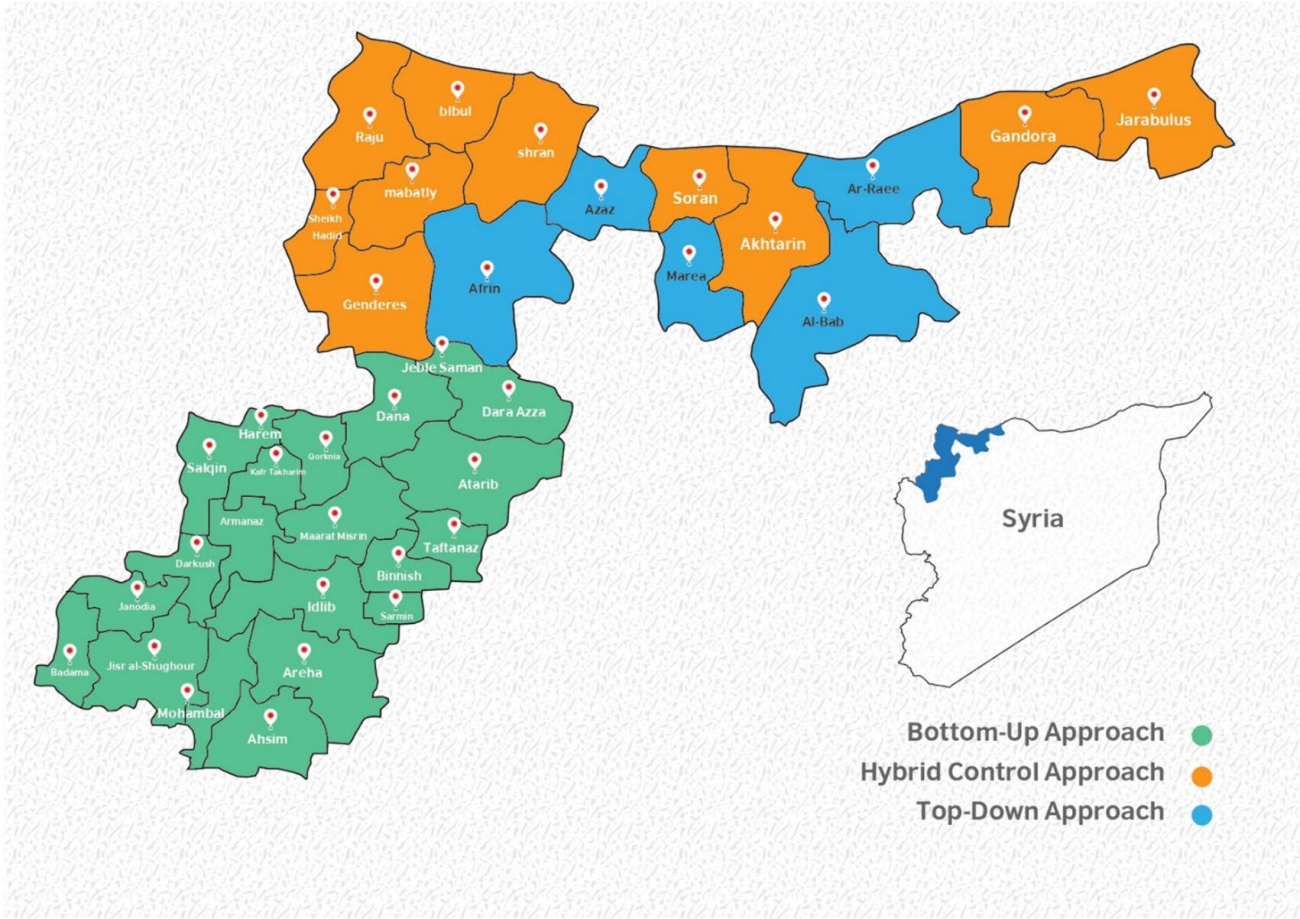
Different types of health governance approaches in northwest Syria

The consultations with experts revealed three distinct approaches to health governance in NWS. In Idlib governorate and western Aleppo, the governance structure follows a bottom-up approach, encompassing 20 sub-districts (Fig. 1, green). In northern Aleppo, two primary approaches were identified: a top-down approach in 5 sub-districts (Fig. 1, blue) and a hybrid approach in 10 sub-districts (Fig. 1, orange). However, the hybrid approach is more aligned with the top-down model, operating under the strategic plan of the Turkish health authorities, as noted by participants in the consultations.

### Quantitative results

#### Average scale of HSLI, sub-indices and indicators across the governance models

Table 4 illustrates that HSLI ranks highest in the bottom-up governance model, followed by the hybrid model, and is lowest in the top-down model. These differences across governance models are statistically significant (Appendix 2). This trend is similarly observed in the four sub-indices. Statistically significant differences exist across all governance models except for the difference between hybrid and top-down for the Act of Consent sub-index and between bottom-up and hybrid for the View of Performance sub-index (Appendix 2). The View of Performance sub-index has the highest average score across all three governance models, while the View of Justification sub-index has the lowest average score. The average score of substitutive indicators is better than that of constitutive indicators in all governance models.

**Table 4:**
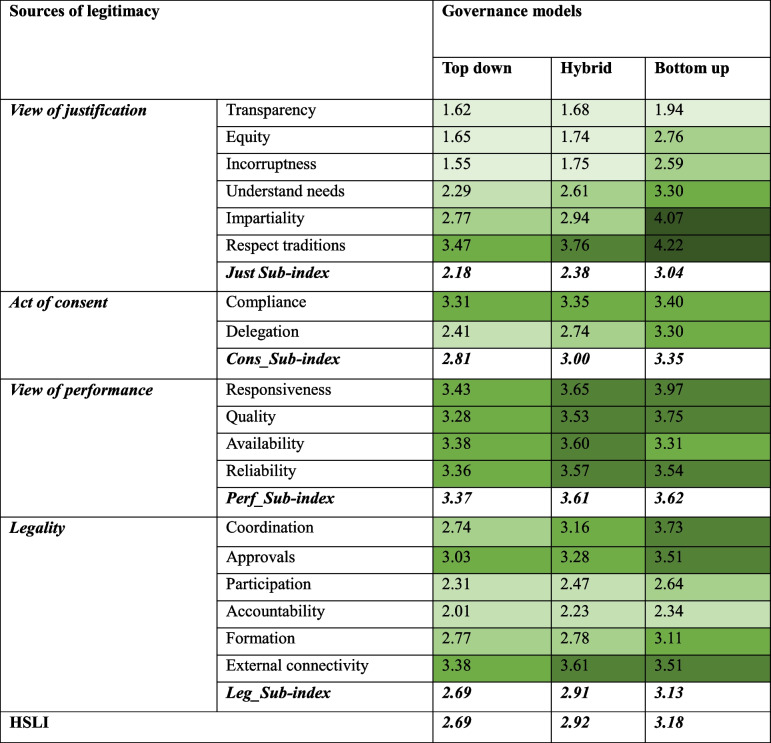
Average scale^a^ of HSLI, its sub-indices^b^ and indicators^c^ across the governance models

The color is darker with bigger values

^a^The scale ranges from 1 to 5, with 1 representing ‘very bad’ and 5 representing ‘very good.’ Although HSLI and its sub-indices are derived from various ordinal variables, their values cover a wide range of categories with very small differences between adjacent categories. As a result, they are treated as continuous variables. Moreover, given the large sample size, ANOVA is generally robust to possible violations of normality [98]

^b^ANOVA tests were conducted to assess statistically significant differences (*P*<0.05) among the means of HSLI and its four sub-indices across different governance models (Appendix 2)

^c^Multinomial logistic regression were conducted to assess statistically significant differences (*P*<0.05) among the mean of the categorical indicators across different governance models (Appendix 2)

Moreover, the analysis reveals no statistically significant differences observed across the three governance models concerning four indicators (Appendix 2). These indicators include ‘compliance’ of the Act of Consent sub-index, ‘reliability’ of the View of Performance sub-index, and ‘approval’ and ‘participation’ within the Legality sub-index. The remaining 14 indicators within the bottom-up governance model exhibit statistically higher values compared to those within the top-down governance model. Conversely, the differences between these indicators within the hybrid governance model, in comparison to both the bottom-up and top-down governance models, do not reach statistical significance (Appendix 2).

#### Percentage distribution of HSLI and sub-indices levels across the governance models

**Fig. 2:**
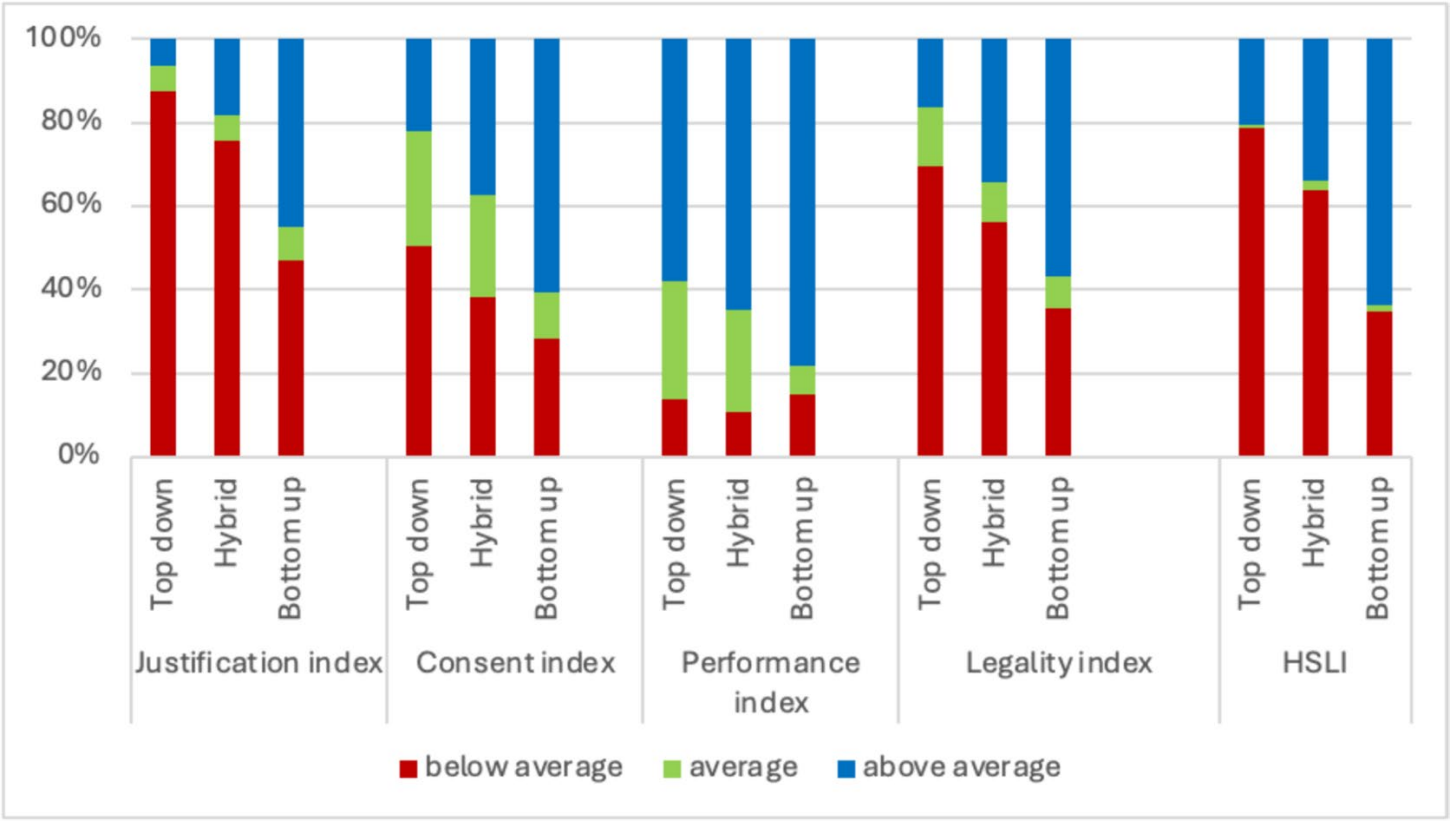
Percentage distribution of HSLI* level and its sub-indices by governance models**, * HSLI and its sub-indices are continuous variables ranging from 1 to 5, with 1 indicating very poor and 5 signifying very good. Thus, we consider any value below 3 as ‘below average’, while values above 3 are classified as ‘above average’, and a value of 3 is assigned as ‘average’. ** We used a two-sample test of proportions to check for statistical significance between each pair of percentages within the same scale category across the three governance models (Appendix 2)

Figure 2 presents the percentage distribution of HSLI and its sub-indices across three governance models, categorized as ‘below average,’ ‘average,’ or ‘above average.’ The HSLI results show that the percentage of ‘above average’ is highest in the bottom-up governance model (63.6%), followed by the hybrid model (34.1%), while the top-down governance model exhibits the lowest percentage (20.4%). These differences between percentages are substantial and statistically significant. The ‘average’ level of HSLI is the lowest across the three governance models; however, the differences in ‘average’ levels among the different governance models are statistically insignificant.

In terms of HSLI sub-indices, the bottom-up model consistently exhibits statistically significant higher proportions of the ‘above average’ across all sub-indices compared to the other two governance models. Conversely, the top-down governance model consistently shows statistically significant higher proportions of scores in the ‘below average’ across all sub-indices, except for the Performance sub-index. It is worth noting that the Performance sub-index shows relatively low ‘below average’ scores across all governance models, with insignificant differences observed between them.

#### Percentage distribution of legitimacy indicators across the governance models

Table 5 provides a detailed percentage distribution of the indicators within the four sub-indices across the three governance models. These indicators are categorised as very bad, bad, average, good, very good, don’t want to answer, don’t know. Analysing the table highlights crucial patterns and disparities between governance models, complementing the insights derived from the analysis of the average scale in Table 4. For instance, when examining the View of Justification sub-index, which includes transparency and equity in addition to other indicators, the bottom-up model consistently demonstrates relatively higher proportions of scores in the ‘good’ and ‘v. good’ categories across these indicators. These high scores could suggest that bottom-up governance structures foster greater transparency, equity, impartiality, and understanding in public health services decision-making compared to more centralized governance models.

**Table 5:** The percentage of people’s perceptions of legitimacy indicators

Subtype/ subindex	Indicator	Governance model	V.Bad	Bad	Avg	Good	V.Good	Dw^a^	Dk^a^
**View of Justification**	***Transparency***	Top_Down	54.4	21.2	11.9	3.1	0.5	2.6	6.2
		Hybrid	50.4	29.9	15.0	0.0	1.6	0.8	2.4
		Bottom_up	38.1	33.8	14.0	6.1	2.1	0.3	5.6
	***Equity***	Top_Down	53.9	21.2	14.0	3.1	0.5	2.6	4.7
		Hybrid	46.5	33.9	15.8	1.6	0.8	0.8	0.8
		Bottom_up	21.5	28.7	12.7	19.6	14.6	0.3	2.6
	***Incorruptness***	Top_Down	58.0	20.7	8.8	4.2	0.0	3.1	5.2
		Hybrid	44.9	32.3	15.0	3.2	0.0	2.4	2.4
		Bottom_up	19.4	28.7	17.2	16.1	7.5	0.3	10.8
	***Understand needs***	Top_Down	18.1	45.6	22.3	8.3	2.6	1.6	1.6
		Hybrid	13.4	37.0	23.6	19.7	3.2	0.0	3.2
		Bottom_up	6.2	20.0	27.2	30.2	16.1	0.1	0.1
	***Impartiality***	Top_Down	7.8	22.8	54.9	9.8	3.1	0.0	1.6
		Hybrid	7.1	31.5	29.9	22.8	8.7	0.0	0.0
		Bottom_up	3.0	11.1	14.3	18.1	52.3	0.3	1.0
	***Respect traditions***	Top_Down	1.0	5.7	50.8	29.5	12.4	0.0	0.5
		Hybrid	0.8	4.7	35.4	36.2	22.8	0.0	0.0
		Bottom_up	1.6	8.8	14.2	15.9	58.7	0.1	0.8
**Act of Consent**	***Compliance***	Top_Down	3.6	9.3	51.8	20.7	13.0	0.0	1.6
		Hybrid	3.9	13.4	43.3	21.3	17.3	0.0	0.8
		Bottom_up	3.4	13.5	36.5	31.1	14.7	0.3	0.5
	***Delegation***	Top_Down	11.9	45.1	21.8	8.8	3.1	2.1	7.3
		Hybrid	9.5	30.7	31.5	11.8	7.1	0.0	9.5
		Bottom_up	11.2	14.4	25.6	28.4	19.1	0.1	1.2
**View of Performance**	***Responsiveness***	Top_Down	1.6	8.8	43.5	35.2	9.8	0.0	1.0
		Hybrid	3.2	8.7	29.9	36.2	22.1	0.0	0.0
		Bottom_up	2.9	7.5	22.2	23.9	42.9	0.3	0.3
	***Quality***	Top_Down	1.0	10.4	51.8	31.6	4.2	1.0	0.0
		Hybrid	3.9	8.7	35.4	32.3	18.1	0.0	1.6
		Bottom_up	1.4	7.7	27.8	38.9	22.6	0.4	1.2
	***Availability***	Top_Down	1.6	5.2	48.7	42.0	2.1	0.5	0.0
		Hybrid	3.2	6.3	37.0	33.1	19.7	0.8	0.0
		Bottom_up	3.0	17.3	39.7	24.7	14.8	0.1	0.4
	***Reliability***	Top_Down	1.0	7.3	48.2	36.8	3.6	1.0	2.1
		Hybrid	1.6	8.7	38.6	29.9	18.9	0.0	2.4
		Bottom_up	2.0	10.0	33.9	34.9	15.6	0.1	3.5
**Legality**	***Coordination***	Top_Down	6.7	31.6	38.9	15.0	2.6	2.1	3.1
		Hybrid	6.3	22.8	30.7	18.9	15.8	0.8	4.7
		Bottom_up	2.7	12.0	22.4	30.6	28.2	0.1	4.0
	***Approvals***	Top_Down	5.2	15.5	44.6	20.2	4.2	4.2	6.2
		Hybrid	5.5	18.1	31.5	23.6	15.8	2.4	3.2
		Bottom_up	2.7	10.8	31.3	27.8	16.9	0.4	10.0
	***Participation***	Top_Down	15.0	45.1	22.8	5.7	3.1	3.1	5.2
		Hybrid	8.7	44.9	27.6	8.7	2.4	0.8	7.1
		Bottom_up	22.0	17.3	37.8	16.4	4.6	0.3	1.7
	***Accountability***	Top_Down	22.8	48.7	18.1	2.1	0.5	5.2	2.6
		Hybrid	16.5	47.2	25.2	3.9	1.6	2.4	3.2
		Bottom_up	33.3	22.2	24.1	15.0	4.4	0.1	0.9
	***Formation***	Top_Down	7.8	20.7	46.1	9.8	3.1	5.2	7.3
		Hybrid	7.9	26.0	32.3	13.4	4.7	2.4	13.4
		Bottom_up	5.7	22.1	32.5	20.6	11.6	1.2	6.4
	***External connectivity***	Top_Down	0.5	5.2	53.4	26.9	7.3	1.6	5.2
		Hybrid	1.6	6.3	40.2	28.4	19.7	0.8	3.2
		Bottom_up	6.0	11.6	27.1	33.9	20.0	0.3	1.2

(^a^): Dw: I do not want to answer, and Dk: I do not know

Similarly, in the Act of Consent sub-index, which includes compliance with guidelines and delegation of authority, the bottom-up model again reveals higher proportions of scores in the ‘good’ and ‘v. good’ categories for these indicators. This could also indicate that the decentralized governance model of public health services may enable more effective compliance and better delegation than hybrid and top-down governance approaches.

In terms of Views of Performance sub-index, the highest percentage of ‘good’ and ‘v. good’ is also seen in the bottom-up governed areas regarding all indicators. However, the top-down model demonstrates relatively better scores in the indicators of this sub-index compared to the top-down indicators of other sub-indices. Within the Legality sub-index, and across all indicators, the bottom-up model consistently demonstrates the highest percentage of indicators classified as ‘good’ and ‘v. good’. In contrast, the top-down model generally displays the lowest percentage of indicators classified as ‘good’ and ‘v. good’.

### Qualitative insights and discussion

From the beginning of our study, we assumed that the international response in the aftermath of the earthquake used the existing structures and governance models and did not change or replace them. This assumption was confirmed by consultancies with local experts. They confirmed that although the response to the earthquake enhanced collaboration between different actors, it did not change governance models.

#### Views of justification

The ‘bottom-up’ model scores are higher across all aspects of the ‘views of justifications’ compared to ‘top-down’ and ‘hybrid’ models. These results suggest that a community-based governance structure, as the ‘bottom-up’ model was described in the expert panel, is more attuned to the local context and specific needs and values.

The lack of transparency in all different models aligns with previous research by Alaref et al., which reported poor transparency in the governance system in NWS. The most significant deficiency was ‘weakness in the internal system or operational model and project design,’ followed by ‘the lack of resources and poor sustainability planning’ and ‘the lack of legitimacy.’ [68] Our participants in the expert panel mentioned more reasons, in areas heavily reliant on aid, medical staff supported by NGOs earn much more than the average person. As a result, financial data is kept private to avoid clashes with local communities. Additionally, sharing health-related information in opposition-controlled areas is risky, as the central government has criminalised health practitioners and systematically targeted health facilities. In regions with poor security, sharing essential information could pose a threat, making institutions vulnerable to armed robbery.

Equity scores below average in all areas. The impact of conflict on health equity is not well-documented, especially in conflict-affected fragile states. Factors such as displacement, gender inequity, and financial barriers can affect health equity [99, 100]. According to participants in the expert panel, the distribution of health services in NWS tends to depend more on institutional systems than on people's needs. For example, since the military campaign by the Syrian and Russian armies started in NWS in 2019 [101], which led to displacing a million people [102] and destroyed more than 60 health facilities [103], more than 200,000 people in Al-Zawya Mountain have remained without facilities. All the attempts by the local health authorities and NGOs to provide health services in this area have failed because of the routine targeting by the Russian and Syrian armies just as they open. Additionally, there is a gap observed in general between people's needs and health and humanitarian aid [45, 104].

Regarding corruption, one of the main challenges in the ‘bottom-up’ area is the weakness of the rule of law and the monopoly of law enforcement by the SSG's courts. Health institutions tend to handle corruption issues internally to avoid dealing with these courts and to comply with red lines drawn by donors regarding interactions with the SSG, according to the expert panel. However, the incorruptibility indicator in the ‘bottom-up’ area is significantly better than that of the ‘top-down’ and ‘hybrid’ areas controlled by the SIG and Turkish authorities. Participants in the expert panel also noted that the system of courts that emerged in northern Aleppo has no authority over Turkish health facilities. Conversely, it is unclear to the public if there is an active internal mechanism to combat corruption in these areas.

Therefore, the ‘bottom-up’ model exhibits acceptable internal regulatory and customary mechanisms to control corruption, with community oversight being more transparent than in other models. However, evidence from a 2012 bribery experiment by Serra comparing top-down and bottom-up accountability indicated that a “combined” accountability system might be highly effective at reducing corruption, even in environments with weak institutions where the likelihood of formal punishment and fines is low [105].

The impartiality score in the ‘bottom-up’ area (4.07) is significant, especially considering the Idlib population of 3 million, 65% of whom are IDPs. More than a million people live in camps. Concerns have been raised about the hostility of local communities and discrimination against IDPs in other sectors. Several studies have investigated the ethical dilemmas faced by medical personnel in overseas humanitarian military operations, including issues of impartiality [106]. However, studies on the impartiality of local medical staff in conflict areas are rare.

According to the expert panel, several factors contribute to this positive result:

1) Entire communities, including their Health Care Workforces (HCWs), were forcibly displaced by the Syrian government from several governorates, such as Daraa, Ghouta, Homs, and Hama, to NWS. These HCWs integrated with the local health sector and became part of the service delivery process. 2) The nature of health system governance, which involves the participation of all health facilities in the elections of the General Assembly and then the Board of Trustees of the IHD, allows for the inclusion of diverse HCW backgrounds in the decision-making process. 3) The majority of the population shares similar political positions regarding the ongoing war in Syria against the Syrian government. And 4) There are no significant ethnic and religious differences, as most people are from the same ethnic and religious background. In contrast, the ‘top-down’ approach, while involving displaced HCWs in providing health services, does not include them in the decision-making process, which is concentrated in the hands of Turkish health officials. Additionally, there are allegations of discrimination against Kurdish people in northern Aleppo by Turkish authorities and the military groups that control the area.

The respect for traditions indicator scores higher in all models compared to other justification values because most HCWs are from the same community or understand its traditions and values.

#### Acts of consent

The overall score of acts of consent is better in the bottom-up governance compared to top-down and hybrid governance. Compliance with HCWs’ orders and advice is above average, with no significant differences among governance approaches. This result demonstrates trust in HCWs who gain significant practical experience in dealing with ‘war medicine,’ especially trauma cases. This result is in line with previous research by Ekzayez et al., which mentioned good compliance to health authorities in Idlib during the response to COVID-19 due to many reasons, including gained experience from prior health emergencies, local-level coordination, community engagement, local health leadership, and the role of diaspora medical networks—using knowledge networks and eHealth tools to great effect [51]. However, the expert panel stressed that the HCWs still need a higher level of professional training to handle such emergencies.

When the contribution of society, including the medical community, in selecting medical leaders increases, even if this is imperfect, people feel more confident in the ability of these leaders to express their interests and act on it, according to a participant in the expert panel. This is a possible explanation for the relatively high delegation score in the ‘bottom-up’ approach in Idlib compared to the ‘top-down’ approach in northern Aleppo. In the ‘top-down’ area, people do not have the right to delegate or remove authorisation by any known mechanism, so the score was below average. However, delegating Turkish health authorities is part of a big dilemma in which Turkish authorities play a role in political negotiation as a partner and sometimes a representative of the opposition. In political science, the extent of delegation in the case of crises and resource constraints is typically influenced by the need for specialisation and efficiency of decision-making of responding bodies, the level of trust, the desire for centralized control, and the situational context [107–109].

#### Views of performance

When evaluating the views of performance of various health systems in NWS in the aftermath of the earthquake, the overall scores of the different health systems were above average.

In the immediate emergency response phase, particularly in the initial days following the earthquake, the ‘bottom-up’ approach exhibited an advantageous capability to swiftly mobilize and deliver urgent health services, outperforming other health systems in terms of the speed of the health response and the quality of health services. This result is in line with previous research by Alzoubi & Alkhalil et al., which emphasised the significance of civil society and bottom-up networks in responding to compound crises compared to governmental institutions and top-down structures [31]. On the other hand, the ‘hybrid’ health system demonstrated a relatively better capacity for maintaining the sustained provision of health services (availability).

By comparing the strengths of these various approaches during different phases of the earthquake response, it is evident that the ‘bottom-up’ system was able to promptly assess immediate healthcare needs, mobilize resources, and establish direct engagement with local communities, which played a crucial role in the rapid deployment of humanitarian health assistance [31]. However, our data shows that such flexibility in decision-making capacity may fall short of ensuring the durability of health services over the long run. Achieving sustained effectiveness necessitates a more comprehensive needs assessment and coordination among responders, including national and international NGOs, as well as adopting long-term policies and procedures that often require a more robust institutional capacity and harmony at the national and sub-national levels. Such requirements are found better in ‘hybrid’ health systems, where the sustainability of health services depends on more centralised management of resources and assessment of long-term needs at the macro level.

Compared to other systems, the ‘top-down’ approach, found in the health governance structures in Azaz, Afrin and al-Bab, for instance, has notably lower scores concerning responsiveness and quality. According to the experts, the internal patient’s referral in the aftermath of the earthquake was clearly from the ‘top-down’ area to the ‘bottom-up’ area in Idlib, especially for advanced specialised services. This could be attributed to the overreliance of the ‘top-down’ structures on the Turkish health authorities, their main conduit of support, which were institutionally overburdened and overwhelmed by the profound impact of the earthquake on the southern provinces of Turkey, thus impeding the efficacy of the health services provided in the aforementioned regions.

The only indicator in the ‘top-down’ approach overtaking that in the ‘bottom-up’ is the ‘availability’ of health services. According to the expert panel, this is because of the significant hospitals built by Turkey in northern Aleppo. Additionally, transferring patients with complicated diseases, including cancers, for treatment in Turkey is also much easier compared to the ‘bottom-up’ area because the ‘top-down’ area applies the Turkish health system.

Globally, national and international frameworks like the 2005 Hyogo Framework for Action by the United Nations have been established to reduce the impact of natural disasters [110]. While a top-down approach is helpful for governments, a bottom-up approach focusing on individual and community responsibility could be even more effective, potentially saving more lives [111].

#### Views of legality

Most respondents indicate a level of coordination between health and humanitarian responders that surpasses the average in areas where the ‘bottom-up’ approach to health governance is implemented, followed by the hybrid system. This can also be linked to the responsive coordination role played by the Health Cluster in Gaziantep in the ‘bottom-up’ area compared to the Turkish health authorities’ role in both ‘top-down’ and ‘hybrid areas’ [31].

The consistently lower scores assigned to all health systems in terms of involving the local community in decision-making processes during the initial response phase (participation), coupled with the deficiencies in community-based accountability of responding bodies, indicate persistent inadequacies in good governance capabilities and institutional capacity beyond the immediate provision of health services. Most respondents expressed dissatisfaction with the local health actors’ level of community-based consultations and their transparency in sharing progress and financial reports in an accessible manner. WHO emphasises community participation as a core element in enhancing primary health care, integrated health services and diminishing health disparities [112–114]. However, despite the growing interest in participation, the evidence linking participation directly to better health remains weak, which creates barriers to gaining full support from governments, funding agencies and health professionals to enhance this concept [72].

All models are below average in terms of accountability. These results align with previous research by Alaref et al., which mentioned poor accountability of the health governance system in the area [68]. However, the ‘Bottom-up’ system has a slightly higher score than the other two systems due to the direct engagement with local communities and their representatives, albeit restricted, in the decision-making mechanisms. Three types of accountabilities were mentioned by Brinkerhoff in 2004, including financial, performance and political, with three purposes: reducing abuse, assuring compliance with procedures and standards, and improving performance [115]. However, criminal accountability is another significant type in conflict zones due to some parties’ involvement in attacking health facilities and medical personnel [116]. The last type is beyond the scope of this paper.

Notably, the public perception of the health systems’ external connectivity in all studied areas, such as the abilities to coordinate with international bodies such as the WHO, and to liaise with and secure funding from external donors, both international donor agencies and the diaspora, surpassed the average, with the ‘hybrid’ system receiving the highest score, closely followed by the ‘bottom-up’ system, and the ‘top-down’ approach, respectively.

The slightly higher rating of the ‘hybrid’ health system regarding ‘external connectivity’ could be due to the profound impact of the earthquake on particular localities within these areas, such as Genderes and its surroundings, which prompted the Syrian diaspora to organise highly effective fundraising campaigns within the first week of the earthquake. According to our respondents, most of these funds were directed towards addressing the specific needs of these affected areas. Additionally, diaspora organisations played a significant role in bridging the gap between donors and local actors, understanding urgent local needs in the aftermath of the earthquake and the first response.

#### Overall legitimacy

To assess the overall legitimacy under different governance models, two key factors need to be considered: the Health System Legitimacy Index (HLSI) scores and the percentage distribution of the index across the ‘below average,’ ‘average,’ and ‘above average’ scales in each governance area. The findings highlight the advantage of the ‘bottom-up’ model in conflict zones, where it is perceived as a more legitimate model.

When examining the HLSI scores, the ‘bottom-up,’ ‘hybrid,’ and ‘top-down’ models scored 3.18, 2.92, and 2.69, respectively. Although the ‘bottom-up’ model outperformed the others, all models scored around the average. This indicates that perceptions regarding health system legitimacy were slightly ‘above average’ in areas where the ‘bottom-up’ model was implemented. However, this slight advantage in a highly volatile and unstable region is considered somewhat muddled.

The superiority of substitutive indicators average over constitutive indicators across all governance models illustrates that people perceive public health authorities' performance as better than the procedures they adopt. This result aligns with Alzoubi & Alkhalil’s findings that undocumented, tacit governance developed during the conflict, relying on health personnel's experience and collective memory, created efficient responses to disasters [31].

## Limitations

- Giving the same weight to the indicators and sub-indices of legitimacy is subjective. Although it is justified in terms of methodology, further studies with different weights depending on the context may be useful.
- In the ‘top-down’ health governance area, the top level is related to foreign authorities (Turkish health institutions), which has an unknown impact on the results and cannot be isolated.
- The influence of neighbouring subdistricts that follow different governance models cannot be isolated because although there are some restrictions between areas due to the internal crossings, mainly between Idlib and northern Aleppo, people can move around and obtain services from other areas. However, people were asked about their perspectives on different legitimacy sources in their area, and the results were statistically significant and justified.

## Conclusion and recommendations

Our study emphasises the importance of factoring in legitimacy in the design and evaluation of health systems in fragmented conflict zones. The framework and index presented in this paper are designed to capture the sources of legitimacy in the health system using 18 substitutive and constitutive indicators.

Health system design and development in conflict zones have significant implications on its legitimacy and subsequently its ability to secure voluntary compliance. The study shows the advantage of the ‘bottom-up’ approach regarding all legitimacy sources’ sub-indices and overall legitimacy, emphasising the importance of community-based governance. The Health System Legitimacy Index (HSLI) score for the ‘bottom-up’ approach is 3.18, with a perception distribution of 63.6% above average. However, the ‘hybrid’ approach shows a slight advantage concerning long-term response indicators, with an HSLI score of 2.92 and a perception distribution of 34.1% above average. The ‘top-down’ approach ranked the lowest in all legitimacy sub-indices and indicators, with an HSLI score of 2.69 and a perception distribution of 20.4% above average. The legitimacy of the health system in all studied areas requires additional efforts to improve and sustain it. Five indicators scored below average across all governance models and need more attention from public health authorities: transparency, equity, incorruptness, participation, and accountability.

Building on these findings, we propose four sets of recommendations for the design and support of health systems in conflict zones.

First, bottom-up health governance in conflict zones should be encouraged and supported with the necessary resources and tools needed to build its capacity. Additionally, strengthening the role of diaspora organisations in aiding emerging health systems in conflict zones is another way to enhance community-based governance because of their commitment to local communities’ interests and their ability to understand donors and local community requirements. The Syrian example demonstrated very well this positive role that diaspora organisations could play.

The ‘bottom-up’ health governance however, especially in conflict-affected areas, must improve its capacity to deal with long-term responses to compound crises. This could be achieved through improving strategic vision and planning, improving international partnerships, and ensuring financial sustainability through a context-specific and context-sensitive approach. While it is difficult to guarantee financial sustainability in conflict settings, helping health systems to have a sustainable basic budget and to diversify their resources is essential to ensure the functionality of health governance. Significant attention should be given to transparency, equity, incorruptness, participation, and accountability, as they all are under the average score.

Second, a balance between top-down and bottom-up governance is essential in responding to emergencies [31]. Modern states possess extensive powers and resources, including legal authority, fiscal, administrative, and informational capabilities, which far exceed those of other non-state actors [41]. However, in conflict areas, the legitimacy and power of national governments are often compromised, necessitating a revaluation of the top-down approach. In the context of NWS, where top-down governance is implemented by a foreign country, the effectiveness of this approach in building trust and legitimacy requires greater scrutiny. The ‘top-down’ health system, represented by the Turkish health authorities in the areas under Turkish control, should establish a meaningful dialogue and negotiation with MoH/SIG, AHD and the Heath cluster in Gaziantep, which includes most of the Syrian NGOs about the governance structure and model in this area to be more inclusive, localised and responsive to the local health needs. Additionally, more attention should be given to transparency, equity, incorruptness, understand needs, impartiality, delegation, coordination, participation, accountability, and formation as all of them are under the average score.

Third, in the area adopted a ‘hybrid’ approach, Community-based governance, should be enhanced since it plays a significant role in improving legitimacy in all governance models. This could include encouraging more democratic mechanisms to represent local communities’ interest in decision-making. Specific attention should be given to transparency, equity, incorruptness, understanding needs, impartiality, delegation, participation, accountability, and formation, as all of them are under the average score.

Fourth, since trust has emerged as a key issue that could improve the effectiveness of health systems, it is important to build the systems and its workers’ capacity to generate and maintain people’s confidence and trust in the leadership and governance system. There are many ways to do that, including creating and supporting precise accountability and transparency mechanisms within the health sector. Additionally, with medical education systems being affected in conflict settings, supporting emerging educational initiatives such as Syrian Board of Medical Specialists and medical colleges would enhance people’s confidence in the medical system [117].

It is important to build on the role that health systems and health workers play in conflict zones beyond their role in providing health services. Most importantly, benefiting from their legitimacy, the health system could play a pivotal role in the peacebuilding process, community dialogue, and the conflict-to-recovery transition.

It is also important to build the capacity of the health system and workers as important upholders of civic virtues such as impartiality and transparency. In that context, it is vital to train them on how to maintain the principle of equity and participation by giving particular attention to women, children, people with disabilities, IDPs, and minorities, as they are usually the most marginalised groups during conflicts.

Our results however demonstrate how delicate is the issue of maintain transparency and fighting corruption in a conflict zone. So, while transparency and free flow of information should be encouraged, it is key to be mindful of its potential consequences in such context. Information-sharing should not endanger operations or put the safety of HCWs and beneficiaries at risk, which means adopting conflict-sensitive transparency (Alkhalil M, Alzoubi Z: From Ground Realities to Policy: A Practical Framework for Assessing Multilateral Health Governance in Conflict-Affected Areas, unpublished). In the absence of credible national mechanisms for ensuring the rule of law, it is important to develop internal anti-corruption mechanisms within health institutions in conflict zones, particularly in ‘top down’ and ‘hybrid’ models.

Finally, while our study shed light on the role of legitimacy as an important dimension of health governance, we believe that more research should be done to discover possible other dimensions that could improve the efficiency of health systems in conflict zones such as power dynamics. This is a significant dimension where there are multipolar health systems with different governance models, such as the situation in Syria. Further research is required to develop a health governance framework in conflict areas.

## Supplementary Information

Supplementary Material 1.

Supplementary Material 2.

## Data Availability

The research team cannot currently share the dataset used in this study due to constraints imposed by the Health Directorate in Idleb, which facilitated the survey process. The Directorate has requested that the data remain confidential. However, sample data, including demographic information (sex, age), distribution by governorate, sub-district, and governance model, and statistical tests, are available in Appendix 2. Future data-sharing procedures will strictly adhere to ethical considerations and relevant data policy guidelines. For inquiries about accessing the dataset once it becomes available for sharing, interested parties can contact the corresponding author, Munzer Alkhalil, for further details.

## Abbreviations

GGIs: Global Governance Institutions
OECD: Organisation for Economic Co-operation and Development
UN: United Nations
HIS: Health Information System
IHD: Idlib Health Directorate
AHD: Aleppo Health Directorate
NGOs: Non-governmental Organisations
INGOs: International Non-governmental Organisations
WHO: World Health Organization
MoH: Ministry of Health
IDPs: Internally Displaced People
SSG: Syrian Salvation Government
LSE: London School of Economics and Political Science
SIG: Syrian Interim Government
HCWs: Health Care Workforces
NWS: Northwest Syria
HSLI: Health System Legitimacy Index
