# Supplementary material for: Capturing sources of health system legitimacy in fragmented conflict zones under different governance models: a case study of northwest Syria
Source: Global Health. 2024 Oct 3;20:71. doi: 10.1186/s12992-024-01074-4 (PMC11451179; doi:10.1186/s12992-024-01074-4)
Supplement: Supplementary file 1 — Supplementary Material 1. [file 12992_2024_1074_MOESM1_ESM.pdf]

Appendix 1
Questionnaire

Capturing sources of health system legitimacy in fragmented conflict zones under different governance models. A case study of northwest Syria.

Munzer Alkhalil, Rim Turkmani, Mazen Gharibah, Preeti Patel, Zaki Mehchy

This questionnaire aims to measure citizens' perception of the multiple sources of the legitimacy of health systems in fragmented conflict zones. It is derived from the health system legitimacy framework the research team has developed for this study. The survey was conducted six months after the earthquake that hit Syria and Turkey on 6 February 2023 in northwest Syria. The health system played a pivotal role as a frontline responder immediately after the earthquake and in addressing multiple health issues in the period following, both on the ground and through representing the local community in the media and in front of international organisations to communicate needs and coordinate appeals. As such, the period after the earthquake was a real test of the efficiency and responsiveness of the health system, and it was also a period when most people had direct experiences with the health system, thus forming views of both its performance and its capacity to represent them.

This questionnaire was conducted in Arabic by local researchers who live in northwest Syria, an area outside the Syrian regime's control.

The text in blue is provided as guidance to the local researcher who is conducting the survey.

I. Information about the interviewee

Respondent must be over 18 years old. Please seek a balance of age, gender, nature of residence (displaced and resident), and different ethnicities (Arab and Kurdish).

Question	Options
Gender	Male, Female
Age	Year of birth
Work	Employed, unemployed
If employed, employment sector	Agriculture, industry, trade, services
Marital status	Married, single, separated, widowed
Accommodation	House, tent
Ethnicity	Arab, Kurdish, other (specify)
Displacement status	Local resident – internally displaced

II. Evaluating sources of legitimacy

How do you rate the following topics from 1 to 5? 1 indicates a very bad rating. 2 bad, 3 average, 4 good and 5 indicates a very good rating. In addition to the options of ‘I do not know’ and ‘I do not wish to answer’.

**Nr**	Question	**Legitimacy sources classification**
**Q 1**	How do you evaluate the **level of coordination** and cooperation among responding agencies?	**Views of Legality**

	It is not necessary for the interviewee to have direct knowledge of the level of coordination, but this could be felt from medical practices and activities on the ground.	
**Q 2**	How do you evaluate the **cooperation of responding bodies with local authorities** and obtaining the necessary licenses and approvals to work in the health response phase of the earthquake?<br>Example: When an organisation or entity carries out a medical activity or opens a facility, do they obtain the necessary licenses to do so from the Idlib Health Directorate, the Ministry of Health in the Salvation Government, the Ministry of Health in the Syrian Interim Government, the medical offices of local councils, and the Turkish authorities - depending on the region? Examples could be given of public health programs such as vaccinations, do vaccine teams have the necessary approvals, do community health workers have the necessary approvals for their work, etc.	
**Q 3**	How do you evaluate **the involvement of the local community in the decision-making process** by the responding agencies?<br>Are people's opinions taken directly or indirectly (through local councils in cases where they are representative) before starting a new medical project in order to evaluate the needs of the community? Is there any role for the community during the project implementation process? Is there a role in evaluating the projects after its completion? When a problem occurs in a medical facility, for example a medical error, is there a role for the community through community leaders in resolving the issue (e.g., the local mayor, the head of the local council - a committee from the village…etc.).	
**Q 4**	How do you evaluate **community accountability** for responding agencies?<br>Does the community exercise a supervisory or directing role for medical organisations or facilities? Is the community, or its representatives, provided with medical facility completion reports, in case there is an issue? Does the community have accountability or pressure mechanisms that can be used?	
**Q 5**	How would you rate the way healthy, responsive bodies were **formed**?<br>This includes the Ministry of Health, Health Directorate, health organisations and facilities that have been or are being established.	
**Q 6**	How do you evaluate the ability of responding agencies to coordinate and secure services and support from **donors** (e.g., in the cases of vaccine campaigns, earthquake response, pandemics)?<br>It is not necessary for the interviewee to know directly what is happening between medical authorities and donors, but he can evaluate this through the availability of certain health services in his area and the availability of some vital programs such as vaccines, advanced treatments such as cardiac surgeries, oncology patients…etc.	
**Q 7**	How would you rate the **transparency** of health responders regarding fundraising and the use of funds for the health response?<br>Example: Have the organisations that launched fundraising campaigns announced the size of the donations collected and how they will be spent? Do people feel that they have access to information about organisations' finances and spendings?	**Views of Justification**

**Q 8**	How do you evaluate the **equity** of distributing resources/services to earthquake-affected areas according to needs of the area?<br>Example: Did the authorities distribute material and in-kind resources to the various health facilities in a fair manner corresponding with their needs and the number of casualties in the regions?	
**Q 9**	How do you evaluate the **incorruptness** of responders regarding the use of resources during the emergency response phase?<br>Example: Do you believe that some of the aid allocated for the earthquake response was stolen or spent inappropriately through rigging tenders, fake projects, very high salaries, paying employees who have nothing to do with the response, hiring people on the basis of favouritism... etc?	
**Q 10**	How do you evaluate the ability of the responding teams to **understand your needs** and issues?<br>Example: Sometimes a doctor may give advice that does not fit with the reality of life in a particular area, such as prescribing high-priced medications to people who are unable to afford it, or advice a change in lifestyles that does not fit with the lifestyle in the area.	
**Q 11**	How do you evaluate the **impartiality** of the responding teams towards those affected?<br>Example: The absence of discrimination between Arab vs. Kurds, displaced vs. local resident, Sunni vs. non-Sunni, military vs. civilian person.	
**Q 12**	How do you evaluate **respect for community customs and traditions** by responding health agencies?<br>Example: respecting traditions when dealing with women and elders. E.g., When a woman is examined, is this done in the presence of the nurse or a relative? Is women's privacy respected during medical examination? Are sensitive issues discussed in a way that respects community customs and traditions such as family planning issues - contraception, early marriage…etc., is priority given to the elderly? Are religious beliefs taken into account when developing treatment plans (e.g., a plan that is compatible with fasting during the month of Ramadan - if possible)	
**Q 13**	How do you evaluate **people's compliance** with health advice and instructions issued by responding agencies? (e.g., transferring patients, discharging patients, decisions on therapeutic interventions, including surgeries, and vaccines)?<br>Example: Do people voluntarily comply when there is a decision by a doctor to transfer the patient to another hospital, or to perform a certain surgical intervention? Do people respond to health instructions regarding public health issues?	**Acts of Consent**
**Q 14**	How to evaluate the level of **delegation** of the local community to the responding bodies to represent community health interests and provide services?<br>Although there is no clear delegation mechanism such as elections, but do people feel comfortable when responsive bodies, such as local authorities, organisations and health directorates meet donors and talk about health problems and needs in this region and obtaining funding to implement projects?	

**Q 15**	How do you evaluate the **speed of the health response** to the earthquake?<br>The assessment includes all stages of the response from the moment the earthquake hit until ambulances started transporting the injured and health facilities received the injured and provide emergency services.	**Views of Performance (Instrumental Legitimacy)**
**Q 16**	How do you evaluate the **quality** of health services provided during the earthquake response?<br>Example: Are health services provided at the right time when the patient needs them? Are the results of the medical service satisfactory?	
**Q 17**	How do you evaluate the **availability** of health services provided after the earthquake?<br>Example: Is there a doctor in the hospital? If a patient needs an x-ray, CT scan, or analysis, is it reasonably available in health facilities?	
**Q 18**	How do you evaluate the **reliability** of health services provided after the earthquake?<br>This includes for example accurate diagnosis, treatment and surgical interventions.	

End of Appendix 2
