# Supplementary material for: Capturing sources of health system legitimacy in fragmented conflict zones under different governance models: a case study of northwest Syria
Source: Global Health. 2024 Oct 3;20:71. doi: 10.1186/s12992-024-01074-4 (PMC11451179; doi:10.1186/s12992-024-01074-4)
Supplement: Supplementary file 2 — Supplementary Material 2. [file 12992_2024_1074_MOESM2_ESM.pdf]

Appendix 2

/*Capturing sources of health system legitimacy in fragmented conflict zones under different governance models. A case study of northwest Syria*/
.

**Munzer Alkhalil, Rim Turkmani, Mazen Gharibah, Preeti Patel, Zaki Mehchy

.
. /*DEMOGRAPHIC INFORMATION*/

sex	Freq.	Percent	Cum.
Female	490	45.00	45.00
Male	599	55.00	100.00
Total	1,089	100.00	

age	Freq.	Percent	Cum.
18_24	137	12.58	12.58
25_33	253	23.23	35.81
34_44	325	29.84	65.66
45_55	215	19.74	85.40
56_64	93	8.54	93.94
64+	66	6.06	100.00
Total	1,089	100.00	

.
. /*DISTRIBUTION BY GOVERNORATE AND MODE OF GOVERNANCE*/

gov	Freq.	Percent	Cum.
Aleppo	384	35.26	35.26

Idleb	705	64.74	100.00
Total	1,089	100.00	

/*TD is top-down approach, MIX is Hybrid approach and BU is Bottom-up approach*/

govmode3	Freq.	Percent	Cum.
TD	193	17.72	17.72
MIX	127	11.66	29.38
BU	769	70.62	100.00
Total	1,089	100.00	

.
. table region govmode3

	govmode3			
	TD	MIX	BU	Total
Region				
A'zaz	77			77
Afrin	45			45
Aghtrin		25		25
Al Bab	50			50
Ar-Ra'ee	6			6
Ariha			26	26
Armanaz			21	21
Atareb			48	48
Badama			15	15
Bennsh			15	15
Dana			278	278
Daret Azza			16	16
Darkosh			22	22

Ehsem			10	10
Harim			19	19
Idleb			69	69
Jandairis		26		26
Janudiyeh			16	16
Jarablus+Ghandoura		23		23
Jisr-Ash-Shugur			27	27
Kafartakharim			9	9
Ma'btali+Sharan		10		10
Maaret Tamsrin			82	82
Mare	15			15
Mhambal			10	10
Qourqeena			26	26
Raju+Blbul		15		15
Salqin			50	50
Sarmin			4	4
Sheikh El-Hadid		3		3
Suran		25		25
Teftnaz			6	6
Total	193	127	769	1,089

.
. /* PERCENTAGE DISTRIBUTION ON A SCLAE FROM 1 TO 5 WHERE 1 IS VERY BAD, IN ADDITION TO DW "DONT WANT TO ANSWER" AND DK "DONT KNOW"*/
.
. **View of Justification sub indicators**
.
. *transparency

	v.bad	bad	average	good	v.good	dw	dk	Total

govmode3								
TD								
transparency	54.40	21.24	11.92	3.11	0.52	2.59	6.22	100.00
govmode3	22.73	12.09	15.33	11.32	5.26	62.50	20.69	17.72
MIX								
transparency	50.39	29.92	14.96		1.57	0.79	2.36	100.00
govmode3	13.85	11.21	12.67		10.53	12.50	5.17	11.66
BU								
transparency	38.10	33.81	14.04	6.11	2.08	0.26	5.59	100.00
govmode3	63.42	76.70	72.00	88.68	84.21	25.00	74.14	70.62
Total								
transparency	42.42	31.13	13.77	4.87	1.74	0.73	5.33	100.00
govmode3	100.00	100.00	100.00	100.00	100.00	100.00	100.00	100.00

.
. *Fairness

	v.bad	bad	average	good	v.good	dw	dk	Total
govmode3								
TD								
justice	53.89	21.24	13.99	3.11	0.52	2.59	4.66	100.00
govmode3	31.71	13.44	18.62	3.77	0.88	62.50	30.00	17.72
MIX								
justice	46.46	33.86	15.75	1.57	0.79	0.79	0.79	100.00
govmode3	17.99	14.10	13.79	1.26	0.88	12.50	3.33	11.66
BU								
justice	21.46	28.74	12.74	19.64	14.56	0.26	2.60	100.00
govmode3	50.30	72.46	67.59	94.97	98.25	25.00	66.67	70.62
Total								
justice	30.12	28.01	13.31	14.60	10.47	0.73	2.75	100.00

govmode3	100.00	100.00	100.00	100.00	100.00	100.00	100.00	100.00

.
. *Incorruptness

	v.bad	bad	average	good	v.good	dw	dk	Total
govmode3								
TD								
corrupt	58.03	20.73	8.81	4.15		3.11	5.18	100.00
govmode3	35.22	13.25	10.12	5.88		54.55	10.42	17.72
MIX								
corrupt	44.88	32.28	14.96	3.15		2.36	2.36	100.00
govmode3	17.92	13.58	11.31	2.94		27.27	3.12	11.66
BU								
corrupt	19.38	28.74	17.17	16.12	7.54	0.26	10.79	100.00
govmode3	46.86	73.18	78.57	91.18	100.00	18.18	86.46	70.62
Total								
corrupt	29.20	27.73	15.43	12.49	5.33	1.01	8.82	100.00
govmode3	100.00	100.00	100.00	100.00	100.00	100.00	100.00	100.00

.
. *Understand needs

	v.bad	bad	average	good	v.good	dw	dk	Total
govmode3								

TD								
capacity	18.13	45.60	22.28	8.29	2.59	1.55	1.55	100.00
govmode3	35.00	30.45	15.25	5.86	3.76	75.00	37.50	17.72
MIX								
capacity	13.39	37.01	23.62	19.69	3.15		3.15	100.00
govmode3	17.00	16.26	10.64	9.16	3.01		50.00	11.66
BU								
capacity	6.24	20.03	27.18	30.17	16.12	0.13	0.13	100.00
govmode3	48.00	53.29	74.11	84.98	93.23	25.00	12.50	70.62
Total								
capacity	9.18	26.54	25.90	25.07	12.21	0.37	0.73	100.00
govmode3	100.00	100.00	100.00	100.00	100.00	100.00	100.00	100.00

.
. *Impartiality

	v.bad	bad	average	good	v.good	dw	dk	Total
govmode3								
TD								
neutral	7.77	22.80	54.92	9.84	3.11		1.55	100.00
govmode3	31.91	26.04	41.73	10.16	1.43		27.27	17.72
MIX								
neutral	7.09	31.50	29.92	22.83	8.66			100.00
govmode3	19.15	23.67	14.96	15.51	2.63			11.66
BU								
neutral	2.99	11.05	14.30	18.08	52.28	0.26	1.04	100.00
govmode3	48.94	50.30	43.31	74.33	95.94	100.00	72.73	70.62
Total								
neutral	4.32	15.52	23.32	17.17	38.48	0.18	1.01	100.00
govmode3	100.00	100.00	100.00	100.00	100.00	100.00	100.00	100.00

```
------------------------------------------------------------------------------------------

.
. *Respect tradition


-------------------------------------------------------------------------------------------
              |
              |   v.bad       bad   average      good    v.good        dw        dk     Total
--------------+----------------------------------------------------------------------------
govmode3      |
  TD          |
    tradition |    1.04      5.70     50.78     29.53     12.44                0.52    100.00
    govmode3  |   13.33     12.94     38.89     25.33      4.76               14.29     17.72
  MIX         |
    tradition |    0.79      4.72     35.43     36.22     22.83                        100.00
    govmode3  |    6.67      7.06     17.86     20.44      5.75                         11.66
  BU          |
    tradition |    1.56      8.84     14.17     15.86     58.65      0.13      0.78    100.00
    govmode3  |   80.00     80.00     43.25     54.22     89.48    100.00     85.71     70.62
  Total       |
    tradition |    1.38      7.81     23.14     20.66     46.28      0.09      0.64    100.00
    govmode3  |  100.00    100.00    100.00    100.00    100.00    100.00    100.00    100.00
-------------------------------------------------------------------------------------------

.
. **Act of consent sub-indicators**
.
. *Compliance


------------------------------------------------------------------------------------------
             |
             |   v.bad       bad   average      good    v.good        dw        dk     Total
-------------+----------------------------------------------------------------------------
```

govmode3								
TD								
commit	3.63	9.33	51.81	20.73	12.95		1.55	100.00
govmode3	18.42	12.95	22.94	13.07	15.62		37.50	17.72
MIX								
commit	3.94	13.39	43.31	21.26	17.32		0.79	100.00
govmode3	13.16	12.23	12.61	8.82	13.75		12.50	11.66
BU								
commit	3.38	13.52	36.54	31.08	14.69	0.26	0.52	100.00
govmode3	68.42	74.82	64.45	78.10	70.62	100.00	50.00	70.62
Total								
commit	3.49	12.76	40.04	28.10	14.69	0.18	0.73	100.00
govmode3	100.00	100.00	100.00	100.00	100.00	100.00	100.00	100.00

.
. *Delegation

	v.bad	bad	average	good	v.good	dw	dk	Total
govmode3								
TD								
represent	11.92	45.08	21.76	8.81	3.11	2.07	7.25	100.00
govmode3	19.01	36.71	15.05	6.80	3.70	80.00	40.00	17.72
MIX								
represent	9.45	30.71	31.50	11.81	7.09		9.45	100.00
govmode3	9.92	16.46	14.34	6.00	5.56		34.29	11.66
BU								
represent	11.18	14.43	25.62	28.35	19.12	0.13	1.17	100.00
govmode3	71.07	46.84	70.61	87.20	90.74	20.00	25.71	70.62
Total								
represent	11.11	21.76	25.62	22.96	14.88	0.46	3.21	100.00
govmode3	100.00	100.00	100.00	100.00	100.00	100.00	100.00	100.00

.
. **View of Performance**
.
. *Responsiveness

	v.bad	bad	average	good	v.good	dw	dk	Total
govmode3								
TD								
timely	1.55	8.81	43.52	35.23	9.84		1.04	100.00
govmode3	10.34	19.77	28.67	22.82	5.04		50.00	17.72
MIX								
timely	3.15	8.66	29.92	36.22	22.05			100.00
govmode3	13.79	12.79	12.97	15.44	7.43			11.66
BU								
timely	2.86	7.54	22.24	23.93	42.91	0.26	0.26	100.00
govmode3	75.86	67.44	58.36	61.74	87.53	100.00	50.00	70.62
Total								
timely	2.66	7.90	26.91	27.36	34.62	0.18	0.37	100.00
govmode3	100.00	100.00	100.00	100.00	100.00	100.00	100.00	100.00

.
. *Quality

	v.bad	bad	average	good	v.good	dw	dk	Total
govmode3								

TD								
quality	1.04	10.36	51.81	31.61	4.15	1.04		100.00
govmode3	11.11	22.22	27.86	15.21	3.90	40.00		17.72
MIX								
quality	3.94	8.66	35.43	32.28	18.11		1.57	100.00
govmode3	27.78	12.22	12.53	10.22	11.22		18.18	11.66
BU								
quality	1.43	7.67	27.83	38.88	22.63	0.39	1.17	100.00
govmode3	61.11	65.56	59.61	74.56	84.88	60.00	81.82	70.62
Total								
quality	1.65	8.26	32.97	36.82	18.82	0.46	1.01	100.00
govmode3	100.00	100.00	100.00	100.00	100.00	100.00	100.00	100.00

.
. *Available

	v.bad	bad	average	good	v.good	dw	dk	Total
govmode3								
TD								
available	1.55	5.18	48.70	41.97	2.07	0.52		100.00
govmode3	10.00	6.62	21.08	25.88	2.80	33.33		17.72
MIX								
available	3.15	6.30	37.01	33.07	19.69	0.79		100.00
govmode3	13.33	5.30	10.54	13.42	17.48	33.33		11.66
BU								
available	2.99	17.30	39.66	24.71	14.82	0.13	0.39	100.00
govmode3	76.67	88.08	68.39	60.70	79.72	33.33	100.00	70.62
Total								
available	2.75	13.87	40.96	28.74	13.13	0.28	0.28	100.00
govmode3	100.00	100.00	100.00	100.00	100.00	100.00	100.00	100.00

.
. *Reliability

	v.bad	bad	average	good	v.good	dw	dk	Total
govmode3								
TD								
credibility	1.04	7.25	48.19	36.79	3.63	1.04	2.07	100.00
govmode3	10.53	13.73	23.08	18.83	4.64	66.67	11.76	17.72
MIX								
credibility	1.57	8.66	38.58	29.92	18.90		2.36	100.00
govmode3	10.53	10.78	12.16	10.08	15.89		8.82	11.66
BU								
credibility	1.95	10.01	33.94	34.85	15.60	0.13	3.51	100.00
govmode3	78.95	75.49	64.76	71.09	79.47	33.33	79.41	70.62
Total								
credibility	1.74	9.37	37.01	34.62	13.87	0.28	3.12	100.00
govmode3	100.00	100.00	100.00	100.00	100.00	100.00	100.00	100.00

.
. **Legality sub indicators**
.
. *Coordination

	v.bad	bad	average	good	v.good	dw	dk	Total
govmode3								

TD								
cooperation	6.74	31.61	38.86	15.03	2.59	2.07	3.11	100.00
govmode3	30.95	33.52	26.22	10.07	2.07	66.67	13.95	17.72
MIX								
cooperation	6.30	22.83	30.71	18.90	15.75	0.79	4.72	100.00
govmode3	19.05	15.93	13.64	8.33	8.26	16.67	13.95	11.66
BU								
cooperation	2.73	11.96	22.37	30.56	28.22	0.13	4.03	100.00
govmode3	50.00	50.55	60.14	81.60	89.67	16.67	72.09	70.62
Total								
cooperation	3.86	16.71	26.26	26.45	22.22	0.55	3.95	100.00
govmode3	100.00	100.00	100.00	100.00	100.00	100.00	100.00	100.00

.
. *Approvals

	v.bad	bad	average	good	v.good	dw	dk	Total
govmode3								
TD								
approval	5.18	15.54	44.56	20.21	4.15	4.15	6.22	100.00
govmode3	26.32	22.06	23.43	13.78	5.06	57.14	12.90	17.72
MIX								
approval	5.51	18.11	31.50	23.62	15.75	2.36	3.15	100.00
govmode3	18.42	16.91	10.90	10.60	12.66	21.43	4.30	11.66
BU								
approval	2.73	10.79	31.34	27.83	16.91	0.39	10.01	100.00
govmode3	55.26	61.03	65.67	75.62	82.28	21.43	82.80	70.62
Total								
approval	3.49	12.49	33.70	25.99	14.51	1.29	8.54	100.00
govmode3	100.00	100.00	100.00	100.00	100.00	100.00	100.00	100.00

.
. *Participation

	v.bad	bad	average	good	v.good	dw	dk	Total
govmode3								
TD								
participation	15.03	45.08	22.80	5.70	3.11	3.11	5.18	100.00
govmode3	13.88	31.41	11.89	7.43	13.64	66.67	31.25	17.72
MIX								
participation	8.66	44.88	27.56	8.66	2.36	0.79	7.09	100.00
govmode3	5.26	20.58	9.46	7.43	6.82	11.11	28.12	11.66
BU								
participation	21.98	17.30	37.84	16.38	4.55	0.26	1.69	100.00
govmode3	80.86	48.01	78.65	85.14	79.55	22.22	40.62	70.62
Total								
participation	19.19	25.44	33.98	13.59	4.04	0.83	2.94	100.00
govmode3	100.00	100.00	100.00	100.00	100.00	100.00	100.00	100.00

.
. *Accountability

	v.bad	bad	average	good	v.good	dw	dk	Total
govmode3								
TD								

	v.bad	bad	average	good	v.good	dw	dk	Total
accountability	22.80	48.70	18.13	2.07	0.52	5.18	2.59	100.00
govmode3	13.71	28.92	13.89	3.23	2.70	71.43	31.25	17.72
MIX								
accountability	16.54	47.24	25.20	3.94	1.57	2.36	3.15	100.00
govmode3	6.54	18.46	12.70	4.03	5.41	21.43	25.00	11.66
BU								
accountability	33.29	22.24	24.06	14.95	4.42	0.13	0.91	100.00
govmode3	79.75	52.62	73.41	92.74	91.89	7.14	43.75	70.62
Total								
accountability	29.48	29.84	23.14	11.39	3.40	1.29	1.47	100.00
govmode3	100.00	100.00	100.00	100.00	100.00	100.00	100.00	100.00

.
. *Formation

	v.bad	bad	average	good	v.good	dw	dk	Total
govmode3								
TD								
formation	7.77	20.73	46.11	9.84	3.11	5.18	7.25	100.00
govmode3	21.74	16.46	23.42	9.79	5.94	45.45	17.50	17.72
MIX								
formation	7.87	25.98	32.28	13.39	4.72	2.36	13.39	100.00
govmode3	14.49	13.58	10.79	8.76	5.94	13.64	21.25	11.66
BU								
formation	5.72	22.11	32.51	20.55	11.57	1.17	6.37	100.00
govmode3	63.77	69.96	65.79	81.44	88.12	40.91	61.25	70.62
Total								
formation	6.34	22.31	34.89	17.81	9.27	2.02	7.35	100.00
govmode3	100.00	100.00	100.00	100.00	100.00	100.00	100.00	100.00

.
. *Fundraising

govmode3	v.bad	bad	average	good	v.good	dw	dk	Total
TD								
fundraise	0.52	5.18	53.37	26.94	7.25	1.55	5.18	100.00
govmode3	2.04	9.35	28.45	14.90	7.25	50.00	43.48	17.72
MIX								
fundraise	1.57	6.30	40.16	28.35	19.69	0.79	3.15	100.00
govmode3	4.08	7.48	14.09	10.32	12.95	16.67	17.39	11.66
BU								
fundraise	5.98	11.57	27.05	33.94	20.03	0.26	1.17	100.00
govmode3	93.88	83.18	57.46	74.79	79.79	33.33	39.13	70.62
Total								
fundraise	4.50	9.83	33.24	32.05	17.72	0.55	2.11	100.00
govmode3	100.00	100.00	100.00	100.00	100.00	100.00	100.00	100.00

.
/*An example of how we tested for significance between percentages across the governance model for each indicator is demonstrated through the "transparency" indicator*/

Proportion test for transparency = 1 (v.bad): TD vs. MIX

Two-sample test of proportions x: Number of obs = 193
y: Number of obs = 127

	Mean	Std. err.	z	P>|z|	[95% conf. interval]	
x	.5440415	.0358509			.473775	.6143079

```
           y |    .503937   .0443665                              .4169804    .5908937
-------------+----------------------------------------------------------------
        diff |   .0401044   .0570409                             -.0716937    .1519026
             |  under H0:   .0570396     0.70   0.482
------------------------------------------------------------------------------
        diff = prop(x) - prop(y)                                  z =   0.7031
    H0: diff = 0

    Ha: diff < 0                 Ha: diff != 0                 Ha: diff > 0
 Pr(Z < z) = 0.7590         Pr(|Z| > |z|) = 0.4820          Pr(Z > z) = 0.2410
Proportion test for transparency = 1 (v.bad): TD vs. BU

Two-sample test of proportions                     x: Number of obs =      193
                                                   y: Number of obs =      769
------------------------------------------------------------------------------
             |       Mean   Std. err.      z    P>|z|     [95% conf. interval]
-------------+----------------------------------------------------------------
           x |   .5440415   .0358509                               .473775    .6143079
           y |   .3810143   .0175125                              .3466905    .4153381
-------------+----------------------------------------------------------------
        diff |   .1630271   .0398995                              .0848255    .2412288
             |  under H0:   .0396508     4.11   0.000
------------------------------------------------------------------------------
        diff = prop(x) - prop(y)                                  z =   4.1116
    H0: diff = 0

    Ha: diff < 0                 Ha: diff != 0                 Ha: diff > 0
 Pr(Z < z) = 1.0000         Pr(|Z| > |z|) = 0.0000          Pr(Z > z) = 0.0000
Proportion test for transparency = 1 (v.bad): MIX vs. BU

Two-sample test of proportions                     x: Number of obs =      127
                                                   y: Number of obs =      769
------------------------------------------------------------------------------
             |       Mean   Std. err.      z    P>|z|     [95% conf. interval]
-------------+----------------------------------------------------------------
```

```
           x |    .503937   .0443665                              .4169804    .5908937
           y |   .3810143   .0175125                              .3466905    .4153381
-------------+----------------------------------------------------------------
        diff |   .1229227   .0476977                               .029437    .2164084
             |  under H0:   .0468932     2.62   0.009
------------------------------------------------------------------------------
        diff = prop(x) - prop(y)                                  z =   2.6213
    H0: diff = 0

    Ha: diff < 0                 Ha: diff != 0                 Ha: diff > 0
 Pr(Z < z) = 0.9956         Pr(|Z| > |z|) = 0.0088          Pr(Z > z) = 0.0044

    Variable |        Obs        Mean    Std. dev.       Min        Max
-------------+---------------------------------------------------------
          TD |         41           1           0          1          1

    Variable |        Obs        Mean    Std. dev.       Min        Max
-------------+---------------------------------------------------------
         MIX |         38           2           0          2          2

    Variable |        Obs        Mean    Std. dev.       Min        Max
-------------+---------------------------------------------------------
          BU |        260           3           0          3          3
Proportion test for transparency = 2(bad): TD vs. MIX

Two-sample test of proportions                     x: Number of obs =      193
                                                   y: Number of obs =      127
------------------------------------------------------------------------------
             |       Mean   Std. err.      z    P>|z|     [95% conf. interval]
-------------+----------------------------------------------------------------
           x |   .2124352   .0294427                              .1547286    .2701419
           y |   .2992126   .0406332                               .219573    .3788522
-------------+----------------------------------------------------------------
        diff |  -.0867774    .050179                             -.1851264    .0115717
             |  under H0:   .0492682    -1.76   0.078
```

```
------------------------------------------------------------------------------
        diff = prop(x) - prop(y)                                  z =  -1.7613
    H0: diff = 0

    Ha: diff < 0                 Ha: diff != 0                 Ha: diff > 0
 Pr(Z < z) = 0.0391         Pr(|Z| > |z|) = 0.0782          Pr(Z > z) = 0.9609
Proportion test for transparency = 2(bad): TD vs. BU

Two-sample test of proportions                     x: Number of obs =      193
                                                   y: Number of obs =      769
------------------------------------------------------------------------------
             |       Mean   Std. err.      z    P>|z|     [95% conf. interval]
-------------+----------------------------------------------------------------
           x |   .2124352   .0294427                      .1547286    .2701419
           y |   .3381014   .0170591                      .3046662    .3715366
-------------+----------------------------------------------------------------
        diff |  -.1256662   .0340277                     -.1923593   -.0589731
             |  under H0:   .0373297    -3.37   0.001
------------------------------------------------------------------------------
        diff = prop(x) - prop(y)                                  z =  -3.3664
    H0: diff = 0

    Ha: diff < 0                 Ha: diff != 0                 Ha: diff > 0
 Pr(Z < z) = 0.0004         Pr(|Z| > |z|) = 0.0008          Pr(Z > z) = 0.9996
Proportion test for transparency = 2(bad): MIX vs. BU

Two-sample test of proportions                     x: Number of obs =      127
                                                   y: Number of obs =      769
------------------------------------------------------------------------------
             |       Mean   Std. err.      z    P>|z|     [95% conf. interval]
-------------+----------------------------------------------------------------
           x |   .2992126   .0406332                       .219573    .3788522
           y |   .3381014   .0170591                      .3046662    .3715366
-------------+----------------------------------------------------------------
        diff |  -.0388888   .0440689                     -.1252624    .0474847
```

```
             |  under H0:   .0451273    -0.86   0.389
------------------------------------------------------------------------------
        diff = prop(x) - prop(y)                                  z =  -0.8618
    H0: diff = 0

    Ha: diff < 0                 Ha: diff != 0                 Ha: diff > 0
 Pr(Z < z) = 0.1944         Pr(|Z| > |z|) = 0.3888          Pr(Z > z) = 0.8056

    Variable |        Obs        Mean    Std. dev.       Min        Max
-------------+---------------------------------------------------------
          TD |         23           1           0          1          1

    Variable |        Obs        Mean    Std. dev.       Min        Max
-------------+---------------------------------------------------------
         MIX |         19           2           0          2          2

    Variable |        Obs        Mean    Std. dev.       Min        Max
-------------+---------------------------------------------------------
          BU |        108           3           0          3          3
Proportion test for transparency = 3 (average): TD vs. MIX

Two-sample test of proportions                    x: Number of obs =      193
                                                  y: Number of obs =      127
------------------------------------------------------------------------------
             |       Mean   Std. err.      z    P>|z|     [95% conf. interval]
-------------+----------------------------------------------------------------
           x |    .119171   .0233213                       .0734621    .1648799
           y |   .1496063   .0316507                       .0875721    .2116405
-------------+----------------------------------------------------------------
        diff |  -.0304353   .0393147                      -.1074908    .0466201
             |  under H0:   .0385826    -0.79   0.430
------------------------------------------------------------------------------
        diff = prop(x) - prop(y)                                  z =  -0.7888
    H0: diff = 0
```

```
    Ha: diff < 0                 Ha: diff != 0                 Ha: diff > 0
 Pr(Z < z) = 0.2151         Pr(|Z| > |z|) = 0.4302          Pr(Z > z) = 0.7849
Proportion test for transparency = 3(average): TD vs. BU

Two-sample test of proportions                     x: Number of obs =      193
                                                   y: Number of obs =      769
------------------------------------------------------------------------------
             |       Mean   Std. err.      z    P>|z|     [95% conf. interval]
-------------+----------------------------------------------------------------
           x |    .119171   .0233213                      .0734621    .1648799
           y |   .1404421   .0125292                      .1158854    .1649989
-------------+----------------------------------------------------------------
        diff |  -.0212711   .0264738                     -.0731589    .0306166
             |  under H0:   .0276126    -0.77   0.441
------------------------------------------------------------------------------
        diff = prop(x) - prop(y)                                  z =  -0.7703
    H0: diff = 0

    Ha: diff < 0                 Ha: diff != 0                 Ha: diff > 0
 Pr(Z < z) = 0.2205         Pr(|Z| > |z|) = 0.4411          Pr(Z > z) = 0.7795
Proportion test for transparency = 3(average): MIX vs. BU

Two-sample test of proportions                     x: Number of obs =      127
                                                   y: Number of obs =      769
------------------------------------------------------------------------------
             |       Mean   Std. err.      z    P>|z|     [95% conf. interval]
-------------+----------------------------------------------------------------
           x |   .1496063   .0316507                      .0875721    .2116405
           y |   .1404421   .0125292                      .1158854    .1649989
-------------+----------------------------------------------------------------
        diff |   .0091642   .0340404                     -.0575537    .0758821
             |  under H0:   .0334077     0.27   0.784
------------------------------------------------------------------------------
        diff = prop(x) - prop(y)                                  z =   0.2743
    H0: diff = 0
```

```
    Ha: diff < 0                 Ha: diff != 0                 Ha: diff > 0
 Pr(Z < z) = 0.6081         Pr(|Z| > |z|) = 0.7838          Pr(Z > z) = 0.3919

    Variable |        Obs        Mean    Std. dev.       Min        Max
-------------+---------------------------------------------------------
          TD |          6           1           0          1          1

    Variable |        Obs        Mean    Std. dev.       Min        Max
-------------+---------------------------------------------------------
         MIX |          0

    Variable |        Obs        Mean    Std. dev.       Min        Max
-------------+---------------------------------------------------------
          BU |         47           3           0          3          3
Proportion test for transparency = 4(good): TD vs. MIX

Two-sample test of proportions                   x: Number of obs =      193
                                                 y: Number of obs =      127
------------------------------------------------------------------------------
             |       Mean   Std. err.      z    P>|z|     [95% conf. interval]
-------------+----------------------------------------------------------------
           x |   .0310881   .0124928                      .0066026    .0555736
           y |          0          0                             0           0
-------------+----------------------------------------------------------------
        diff |   .0310881   .0124928                      .0066026    .0555736
             |  under H0:   .0154983     2.01   0.045
------------------------------------------------------------------------------
        diff = prop(x) - prop(y)                                  z =   2.0059
    H0: diff = 0

    Ha: diff < 0                 Ha: diff != 0                 Ha: diff > 0
 Pr(Z < z) = 0.9776         Pr(|Z| > |z|) = 0.0449          Pr(Z > z) = 0.0224
Proportion test for transparency = 4(good): TD vs. BU
```

```
Two-sample test of proportions                   x: Number of obs =      193
                                                 y: Number of obs =      769
------------------------------------------------------------------------------
             |       Mean   Std. err.      z    P>|z|     [95% conf. interval]
-------------+----------------------------------------------------------------
           x |   .0310881   .0124928                      .0066026    .0555736
           y |   .0611183   .0086383                      .0441876    .0780491
-------------+----------------------------------------------------------------
        diff |  -.0300303   .0151885                     -.0597992   -.0002613
             |  under H0:   .0183692    -1.63   0.102
------------------------------------------------------------------------------
        diff = prop(x) - prop(y)                                  z =  -1.6348
    H0: diff = 0

    Ha: diff < 0                 Ha: diff != 0                 Ha: diff > 0
 Pr(Z < z) = 0.0510         Pr(|Z| > |z|) = 0.1021          Pr(Z > z) = 0.9490
Proportion test for transparency = 4(good): MIX vs. BU

Two-sample test of proportions                   x: Number of obs =      127
                                                 y: Number of obs =      769
------------------------------------------------------------------------------
             |       Mean   Std. err.      z    P>|z|     [95% conf. interval]
-------------+----------------------------------------------------------------
           x |          0          0                             0           0
           y |   .0611183   .0086383                      .0441876    .0780491
-------------+----------------------------------------------------------------
        diff |  -.0611183   .0086383                     -.0780491   -.0441876
             |  under H0:   .0213542    -2.86   0.004
------------------------------------------------------------------------------
        diff = prop(x) - prop(y)                                  z =  -2.8621
    H0: diff = 0

    Ha: diff < 0                 Ha: diff != 0                 Ha: diff > 0
 Pr(Z < z) = 0.0021         Pr(|Z| > |z|) = 0.0042          Pr(Z > z) = 0.9979
```

```
    Variable |        Obs        Mean    Std. dev.       Min        Max
-------------+---------------------------------------------------------
          TD |          1           1           .          1          1

    Variable |        Obs        Mean    Std. dev.       Min        Max
-------------+---------------------------------------------------------
         MIX |          2           2           0          2          2

    Variable |        Obs        Mean    Std. dev.       Min        Max
-------------+---------------------------------------------------------
          BU |         16           3           0          3          3
Proportion test for transparency = 5(v.good): TD vs. MIX

Two-sample test of proportions                     x: Number of obs =      193
                                                   y: Number of obs =      127
------------------------------------------------------------------------------
             |       Mean   Std. err.      z    P>|z|     [95% conf. interval]
-------------+----------------------------------------------------------------
           x |   .0051813   .0051679                     -.0049476    .0153103
           y |    .015748   .0110475                     -.0059047    .0374008
-------------+----------------------------------------------------------------
        diff |  -.0105667   .0121965                     -.0344714     .013338
             |  under H0:   .0110112    -0.96   0.337
------------------------------------------------------------------------------
        diff = prop(x) - prop(y)                                  z =  -0.9596
    H0: diff = 0

    Ha: diff < 0                 Ha: diff != 0                 Ha: diff > 0
 Pr(Z < z) = 0.1686         Pr(|Z| > |z|) = 0.3372          Pr(Z > z) = 0.8314
Proportion test for transparency = 5(v.good): TD vs. BU

Two-sample test of proportions                     x: Number of obs =      193
                                                   y: Number of obs =      769
------------------------------------------------------------------------------
             |       Mean   Std. err.      z    P>|z|     [95% conf. interval]
```

```
-------------+----------------------------------------------------------------
           x |   .0051813   .0051679                      -.0049476    .0153103
           y |   .0208062   .0051472                        .010718    .0308945
-------------+----------------------------------------------------------------
        diff |  -.0156249   .0072939                      -.0299206   -.0013292
             |  under H0:   .0106075    -1.47   0.141
------------------------------------------------------------------------------
        diff = prop(x) - prop(y)                                  z =  -1.4730
    H0: diff = 0

    Ha: diff < 0                 Ha: diff != 0                 Ha: diff > 0
 Pr(Z < z) = 0.0704         Pr(|Z| > |z|) = 0.1407          Pr(Z > z) = 0.9296
Proportion test for transparency = 5(v.good): MIX vs. BU

Two-sample test of proportions                     x: Number of obs =      127
                                                   y: Number of obs =      769
------------------------------------------------------------------------------
             |       Mean   Std. err.      z    P>|z|     [95% conf. interval]
-------------+----------------------------------------------------------------
           x |    .015748   .0110475                      -.0059047    .0374008
           y |   .0208062   .0051472                        .010718    .0308945
-------------+----------------------------------------------------------------
        diff |  -.0050582   .0121877                      -.0289457    .0188293
             |  under H0:   .0134389    -0.38   0.707
------------------------------------------------------------------------------
        diff = prop(x) - prop(y)                                  z =  -0.3764
    H0: diff = 0

    Ha: diff < 0                 Ha: diff != 0                 Ha: diff > 0
 Pr(Z < z) = 0.3533         Pr(|Z| > |z|) = 0.7066          Pr(Z > z) = 0.6467

    Variable |        Obs        Mean    Std. dev.       Min        Max
-------------+---------------------------------------------------------
          TD |          5           1           0          1          1
```

```
    Variable |        Obs        Mean    Std. dev.       Min        Max
-------------+---------------------------------------------------------
         MIX |          1           2           .          2          2

    Variable |        Obs        Mean    Std. dev.       Min        Max
-------------+---------------------------------------------------------
          BU |          2           3           0          3          3
Proportion test for transparency = 6(dw): TD vs. MIX

Two-sample test of proportions                    x: Number of obs =      193
                                                  y: Number of obs =      127
------------------------------------------------------------------------------
             |       Mean   Std. err.      z    P>|z|     [95% conf. interval]
-------------+----------------------------------------------------------------
           x |   .0259067   .0114348                       .003495    .0483185
           y |    .007874    .007843                     -.0074979    .0232459
-------------+----------------------------------------------------------------
        diff |   .0180327    .013866                     -.0091442    .0452096
             |  under H0:   .0154983     1.16   0.245
------------------------------------------------------------------------------
        diff = prop(x) - prop(y)                                  z =   1.1635
    H0: diff = 0

    Ha: diff < 0                 Ha: diff != 0                 Ha: diff > 0
 Pr(Z < z) = 0.8777         Pr(|Z| > |z|) = 0.2446          Pr(Z > z) = 0.1223
Proportion test for transparency = 6(dw): TD vs. BU

Two-sample test of proportions                    x: Number of obs =      193
                                                  y: Number of obs =      769
------------------------------------------------------------------------------
             |       Mean   Std. err.      z    P>|z|     [95% conf. interval]
-------------+----------------------------------------------------------------
           x |   .0259067   .0114348                       .003495    .0483185
           y |   .0026008   .0018366                       -.000999    .0062005
-------------+----------------------------------------------------------------
```

```
        diff |    .023306   .0115813                      .0006069     .046005
             |  under H0:   .0068426     3.41   0.001
------------------------------------------------------------------------------
        diff = prop(x) - prop(y)                                  z =   3.4060
    H0: diff = 0

    Ha: diff < 0                 Ha: diff != 0                 Ha: diff > 0
 Pr(Z < z) = 0.9997         Pr(|Z| > |z|) = 0.0007          Pr(Z > z) = 0.0003
Proportion test for transparency = 6(dw): MIX vs. BU

Two-sample test of proportions                     x: Number of obs =      127
                                                   y: Number of obs =      769
------------------------------------------------------------------------------
             |       Mean   Std. err.      z    P>|z|     [95% conf. interval]
-------------+----------------------------------------------------------------
           x |    .007874    .007843                     -.0074979    .0232459
           y |   .0026008   .0018366                      -.000999    .0062005
-------------+----------------------------------------------------------------
        diff |   .0052732   .0080551                     -.0105145     .021061
             |  under H0:   .0055331     0.95   0.341
------------------------------------------------------------------------------
        diff = prop(x) - prop(y)                                  z =   0.9530
    H0: diff = 0

    Ha: diff < 0                 Ha: diff != 0                 Ha: diff > 0
 Pr(Z < z) = 0.8297         Pr(|Z| > |z|) = 0.3406          Pr(Z > z) = 0.1703

    Variable |        Obs        Mean    Std. dev.       Min        Max
-------------+---------------------------------------------------------
          TD |         12           1           0          1          1

    Variable |        Obs        Mean    Std. dev.       Min        Max
-------------+---------------------------------------------------------
         MIX |          3           2           0          2          2
```

```
    Variable |        Obs        Mean    Std. dev.       Min        Max
-------------+---------------------------------------------------------
          BU |         43           3           0          3          3
Proportion test for transparency = 7(dk): TD vs. MIX

Two-sample test of proportions                    x: Number of obs =      193
                                                  y: Number of obs =      127
------------------------------------------------------------------------------
             |       Mean   Std. err.      z    P>|z|     [95% conf. interval]
-------------+----------------------------------------------------------------
           x |   .0621762   .0173818                      .0281085    .0962438
           y |    .023622   .0134762                     -.0027907    .0500348
-------------+----------------------------------------------------------------
        diff |   .0385541   .0219939                     -.0045532    .0816614
             |  under H0:   .0241513     1.60   0.110
------------------------------------------------------------------------------
        diff = prop(x) - prop(y)                                  z =   1.5964
    H0: diff = 0

    Ha: diff < 0                 Ha: diff != 0                 Ha: diff > 0
 Pr(Z < z) = 0.9448         Pr(|Z| > |z|) = 0.1104          Pr(Z > z) = 0.0552
Proportion test for transparency = 7(dk): TD vs. BU

Two-sample test of proportions                    x: Number of obs =      193
                                                  y: Number of obs =      769
------------------------------------------------------------------------------
             |       Mean   Std. err.      z    P>|z|     [95% conf. interval]
-------------+----------------------------------------------------------------
           x |   .0621762   .0173818                      .0281085    .0962438
           y |   .0559168   .0082854                      .0396777    .0721558
-------------+----------------------------------------------------------------
        diff |   .0062594   .0192555                     -.0314807    .0439994
             |  under H0:    .018692     0.33   0.738
------------------------------------------------------------------------------
        diff = prop(x) - prop(y)                                  z =   0.3349
```

```
    H0: diff = 0

    Ha: diff < 0                 Ha: diff != 0                 Ha: diff > 0
 Pr(Z < z) = 0.6311         Pr(|Z| > |z|) = 0.7377          Pr(Z > z) = 0.3689
Proportion test for transparency = 7(dk): MIX vs. BU

Two-sample test of proportions                     x: Number of obs =      127
                                                   y: Number of obs =      769
------------------------------------------------------------------------------
             |       Mean   Std. err.      z    P>|z|     [95% conf. interval]
-------------+----------------------------------------------------------------
           x |    .023622   .0134762                     -.0027907    .0500348
           y |   .0559168   .0082854                      .0396777    .0721558
-------------+----------------------------------------------------------------
        diff |  -.0322947   .0158194                     -.0633002   -.0012892
             |  under H0:   .0211383    -1.53   0.127
------------------------------------------------------------------------------
        diff = prop(x) - prop(y)                                  z =  -1.5278
    H0: diff = 0

    Ha: diff < 0                 Ha: diff != 0                 Ha: diff > 0
 Pr(Z < z) = 0.0633         Pr(|Z| > |z|) = 0.1266          Pr(Z > z) = 0.9367


.
. /*CONSTRUCTING HLSI AND ITS FOUR SUB-INDICES*/
.
. **View of justification subindex
.
. gen just_ind= (transparency+justice+corrupt+capacity+neutral+tradition)/6

. alpha transparency justice corrupt capacity neutral tradition

Test scale = mean(unstandardized items)
```

```
Average interitem covariance:     .6157643
Number of items in the scale:            6
Scale reliability coefficient:      0.8261
. /* C-alpha analysis shows very good internal consistency between the indicators constructing this
subindex*/
.
-> govmode3 = TD

    Variable |        Obs        Mean    Std. dev.       Min        Max
-------------+---------------------------------------------------------
    just_ind |        167    2.176647    .5664365   1.166667          4


-> govmode3 = MIX

    Variable |        Obs        Mean    Std. dev.       Min        Max
-------------+---------------------------------------------------------
    just_ind |        114    2.383041      .68087   1.166667          4


-> govmode3 = BU

    Variable |        Obs        Mean    Std. dev.       Min        Max
-------------+---------------------------------------------------------
    just_ind |        638    3.041275    .8181738   1.166667          5


-----------------------------------------------------------------------------------------------------
-----------------------------------------------------
-> govmode3 = TD

    just_ind1 |      Freq.     Percent        Cum.
--------------+-----------------------------------
below average |        146       87.43       87.43
      average |         10        5.99       93.41
```

```
above average |         11        6.59      100.00
--------------+-----------------------------------
        Total |        167      100.00

-------------------------------------------------------------------------------
-> govmode3 = MIX

    just_ind1 |      Freq.     Percent        Cum.
--------------+-----------------------------------
below average |         86       75.44       75.44
      average |          7        6.14       81.58
above average |         21       18.42      100.00
--------------+-----------------------------------
        Total |        114      100.00

-------------------------------------------------------------------------------
-> govmode3 = BU

    just_ind1 |      Freq.     Percent        Cum.
--------------+-----------------------------------
below average |        299       46.87       46.87
      average |         53        8.31       55.17
above average |        286       44.83      100.00
--------------+-----------------------------------
        Total |        638      100.00

. 
. *View of Justification two-sample test for significancy
. 
/*      TD_Mix  below average   Just    */

Two-sample test of proportions                   x: Number of obs =      167
                                                 y: Number of obs =      114
------------------------------------------------------------------------------
             |       Mean   Std. err.      z    P>|z|     [95% conf. interval]
```

```
--------------+----------------------------------------------------------------
            x |   .8742515   .0256573                      .8239641    .9245389
            y |    .754386   .0403154                      .6753692    .8334027
--------------+----------------------------------------------------------------
         diff |   .1198655   .0477873                      .0262041     .213527
              |  under H0:   .0460976     2.60   0.009
-------------------------------------------------------------------------------
         diff = prop(x) - prop(y)                                  z =   2.6003
    H0: diff = 0

    Ha: diff < 0                 Ha: diff != 0                 Ha: diff > 0
 Pr(Z < z) = 0.9953         Pr(|Z| > |z|) = 0.0093          Pr(Z > z) = 0.0047


/*      TD_BU   below average   Just    */

Two-sample test of proportions                   x: Number of obs =      167
                                                 y: Number of obs =      638
-------------------------------------------------------------------------------
              |       Mean   Std. err.      z    P>|z|     [95% conf. interval]
--------------+----------------------------------------------------------------
            x |   .8742515   .0256573                      .8239641    .9245389
            y |    .468652   .0197562                      .4299305    .5073736
--------------+----------------------------------------------------------------
         diff |   .4055995   .0323822                      .3421315    .4690674
              |  under H0:    .043218     9.38   0.000
-------------------------------------------------------------------------------
         diff = prop(x) - prop(y)                                  z =   9.3850
    H0: diff = 0

    Ha: diff < 0                 Ha: diff != 0                 Ha: diff > 0
 Pr(Z < z) = 1.0000         Pr(|Z| > |z|) = 0.0000          Pr(Z > z) = 0.0000


/*      Mix_BU  below average   Just    */
```

```
Two-sample test of proportions                   x: Number of obs =      114
                                                 y: Number of obs =      638
------------------------------------------------------------------------------
             |       Mean   Std. err.      z    P>|z|     [95% conf. interval]
-------------+----------------------------------------------------------------
           x |    .754386   .0403154                      .6753692    .8334027
           y |    .468652   .0197562                      .4299305    .5073736
-------------+----------------------------------------------------------------
        diff |   .2857339   .0448959                      .1977396    .3737282
             |  under H0:   .0508267     5.62   0.000
------------------------------------------------------------------------------
        diff = prop(x) - prop(y)                                  z =   5.6217
    H0: diff = 0

    Ha: diff < 0                 Ha: diff != 0                 Ha: diff > 0
 Pr(Z < z) = 1.0000         Pr(|Z| > |z|) = 0.0000          Pr(Z > z) = 0.0000


/*      TD_Mix  average Just    */

Two-sample test of proportions                   x: Number of obs =      167
                                                 y: Number of obs =      114
------------------------------------------------------------------------------
             |       Mean   Std. err.      z    P>|z|     [95% conf. interval]
-------------+----------------------------------------------------------------
           x |   .0598802   .0183601                      .0238951    .0958654
           y |   .0614035   .0224845                      .0173347    .1054724
-------------+----------------------------------------------------------------
        diff |  -.0015233   .0290284                     -.0584179    .0553713
             |  under H0:   .0289643    -0.05   0.958
------------------------------------------------------------------------------
        diff = prop(x) - prop(y)                                  z =  -0.0526
    H0: diff = 0
```

```
    Ha: diff < 0                 Ha: diff != 0                 Ha: diff > 0
 Pr(Z < z) = 0.4790         Pr(|Z| > |z|) = 0.9581          Pr(Z > z) = 0.5210


/*      TD_BU   average Just    */

Two-sample test of proportions                    x: Number of obs =      167
                                                  y: Number of obs =      638
------------------------------------------------------------------------------
             |       Mean   Std. err.      z    P>|z|     [95% conf. interval]
-------------+----------------------------------------------------------------
           x |   .0598802   .0183601                      .0238951    .0958654
           y |   .0830721   .0109266                      .0616564    .1044878
-------------+----------------------------------------------------------------
        diff |  -.0231919   .0213655                     -.0650674    .0186837
             |  under H0:   .0233456    -0.99   0.321
------------------------------------------------------------------------------
        diff = prop(x) - prop(y)                                  z =  -0.9934
    H0: diff = 0

    Ha: diff < 0                 Ha: diff != 0                 Ha: diff > 0
 Pr(Z < z) = 0.1603         Pr(|Z| > |z|) = 0.3205          Pr(Z > z) = 0.8397


/*      Mix_BU  average Just    */

Two-sample test of proportions                    x: Number of obs =      114
                                                  y: Number of obs =      638
------------------------------------------------------------------------------
             |       Mean   Std. err.      z    P>|z|     [95% conf. interval]
-------------+----------------------------------------------------------------
           x |   .0614035   .0224845                      .0173347    .1054724
           y |   .0830721   .0109266                      .0616564    .1044878
-------------+----------------------------------------------------------------
        diff |  -.0216686   .0249989                     -.0706655    .0273283
```

```
             |  under H0:   .0275522    -0.79   0.432
------------------------------------------------------------------------------
        diff = prop(x) - prop(y)                                  z =  -0.7865
    H0: diff = 0

    Ha: diff < 0                 Ha: diff != 0                 Ha: diff > 0
 Pr(Z < z) = 0.2158         Pr(|Z| > |z|) = 0.4316          Pr(Z > z) = 0.7842

/*      TD_Mix  above average   Just    */

Two-sample test of proportions                     x: Number of obs =      167
                                                   y: Number of obs =      114
------------------------------------------------------------------------------
             |       Mean   Std. err.      z    P>|z|     [95% conf. interval]
-------------+----------------------------------------------------------------
           x |   .0658683   .0191948                      .0282471    .1034894
           y |   .1842105   .0363073                      .1130495    .2553715
-------------+----------------------------------------------------------------
        diff |  -.1183423    .041069                      -.198836   -.0378486
             |  under H0:   .0385932    -3.07   0.002
------------------------------------------------------------------------------
        diff = prop(x) - prop(y)                                  z =  -3.0664
    H0: diff = 0

    Ha: diff < 0                 Ha: diff != 0                 Ha: diff > 0
 Pr(Z < z) = 0.0011         Pr(|Z| > |z|) = 0.0022          Pr(Z > z) = 0.9989

/*      TD_BU   above average   Just    */

Two-sample test of proportions                     x: Number of obs =      167
                                                   y: Number of obs =      638
------------------------------------------------------------------------------
             |       Mean   Std. err.      z    P>|z|     [95% conf. interval]
-------------+----------------------------------------------------------------
           x |   .0658683   .0191948                      .0282471    .1034894
```

```
           y |   .4482759    .019689                         .4096862    .4868656
-------------+----------------------------------------------------------------
        diff |  -.3824076   .0274972                        -.4363012    -.328514
             |  under H0:   .0419415    -9.12   0.000
------------------------------------------------------------------------------
        diff = prop(x) - prop(y)                                  z =  -9.1177
    H0: diff = 0

    Ha: diff < 0                 Ha: diff != 0                 Ha: diff > 0
 Pr(Z < z) = 0.0000         Pr(|Z| > |z|) = 0.0000          Pr(Z > z) = 1.0000

/*      Mix_BU  above average   Just    */

Two-sample test of proportions                     x: Number of obs =      114
                                                   y: Number of obs =      638
------------------------------------------------------------------------------
             |       Mean   Std. err.      z    P>|z|     [95% conf. interval]
-------------+----------------------------------------------------------------
           x |   .1842105   .0363073                         .1130495    .2553715
           y |   .4482759    .019689                         .4096862    .4868656
-------------+----------------------------------------------------------------
        diff |  -.2640653   .0413022                        -.3450162   -.1831144
             |  under H0:   .0499778    -5.28   0.000
------------------------------------------------------------------------------
        diff = prop(x) - prop(y)                                  z =  -5.2836
    H0: diff = 0

    Ha: diff < 0                 Ha: diff != 0                 Ha: diff > 0
 Pr(Z < z) = 0.0000         Pr(|Z| > |z|) = 0.0000          Pr(Z > z) = 1.0000


.
. **Act of consent subindex
. gen consent_ind= (commit+represent)/2
```

```
Test scale = mean(unstandardized items)

Average interitem covariance:     .5172275
Number of items in the scale:            2
Scale reliability coefficient:      0.5802
. /* C-alpha analysis shows an acceptable internal consistency between the indicators constructing this
subindex*/
.
--------------------------------------------------------------
-> govmode3 = TD

    Variable |        Obs        Mean    Std. dev.       Min        Max
-------------+---------------------------------------------------------
 consent_ind |        173    2.806358    .7081872          1          5

--------------------------------------------------------------
-> govmode3 = MIX

    Variable |        Obs        Mean    Std. dev.       Min        Max
-------------+---------------------------------------------------------
 consent_ind |        115    3.004348    .8823206          1          5

--------------------------------------------------------------
-> govmode3 = BU

    Variable |        Obs        Mean    Std. dev.       Min        Max
-------------+---------------------------------------------------------
 consent_ind |        754    3.345491    .9633039          1          5


----------------------------------------------------------------------------------------------------------
-----------------------------------------------------
-> govmode3 = TD
```

```
 consent_ind1 |      Freq.     Percent        Cum.
--------------+-----------------------------------
below average |         87       50.29       50.29
      average |         48       27.75       78.03
above average |         38       21.97      100.00
--------------+-----------------------------------
        Total |        173      100.00

-------------------------------------------------------------------------------
-> govmode3 = MIX

 consent_ind1 |      Freq.     Percent        Cum.
--------------+-----------------------------------
below average |         44       38.26       38.26
      average |         28       24.35       62.61
above average |         43       37.39      100.00
--------------+-----------------------------------
        Total |        115      100.00

-------------------------------------------------------------------------------
-> govmode3 = BU

 consent_ind1 |      Freq.     Percent        Cum.
--------------+-----------------------------------
below average |        212       28.12       28.12
      average |         84       11.14       39.26
above average |        458       60.74      100.00
--------------+-----------------------------------
        Total |        754      100.00


. *Act of consent two-sample test for significancy
.
 /*      TD_Mix  below average   Consent */
```

```
Two-sample test of proportions                   x: Number of obs =      173
                                                 y: Number of obs =      115
------------------------------------------------------------------------------
             |       Mean   Std. err.      z    P>|z|     [95% conf. interval]
-------------+----------------------------------------------------------------
           x |   .5028902   .0380137                      .4283848    .5773956
           y |   .3826087    .045322                      .2937793    .4714381
-------------+----------------------------------------------------------------
        diff |   .1202815   .0591534                       .004343    .2362199
             |  under H0:   .0599125     2.01   0.045
------------------------------------------------------------------------------
        diff = prop(x) - prop(y)                                  z =   2.0076
    H0: diff = 0

    Ha: diff < 0                 Ha: diff != 0                 Ha: diff > 0
 Pr(Z < z) = 0.9777         Pr(|Z| > |z|) = 0.0447          Pr(Z > z) = 0.0223

/*      TD_BU   below average   Consent */

Two-sample test of proportions                   x: Number of obs =      173
                                                 y: Number of obs =      754
------------------------------------------------------------------------------
             |       Mean   Std. err.      z    P>|z|     [95% conf. interval]
-------------+----------------------------------------------------------------
           x |   .5028902   .0380137                      .4283848    .5773956
           y |   .2811671   .0163723                      .2490779    .3132563
-------------+----------------------------------------------------------------
        diff |   .2217231   .0413895                      .1406011     .302845
             |  under H0:   .0394064     5.63   0.000
------------------------------------------------------------------------------
        diff = prop(x) - prop(y)                                  z =   5.6266
    H0: diff = 0

    Ha: diff < 0                 Ha: diff != 0                 Ha: diff > 0
 Pr(Z < z) = 1.0000         Pr(|Z| > |z|) = 0.0000          Pr(Z > z) = 0.0000
```

```
/*      Mix_BU  below average   Consent */

Two-sample test of proportions                     x: Number of obs =      115
                                                   y: Number of obs =      754
------------------------------------------------------------------------------
             |       Mean   Std. err.      z    P>|z|     [95% conf. interval]
-------------+----------------------------------------------------------------
           x |   .3826087    .045322                      .2937793    .4714381
           y |   .2811671   .0163723                      .2490779    .3132563
-------------+----------------------------------------------------------------
        diff |   .1014416   .0481885                      .0069938    .1958894
             |  under H0:   .0456358     2.22   0.026
------------------------------------------------------------------------------
        diff = prop(x) - prop(y)                                  z =   2.2229
    H0: diff = 0

    Ha: diff < 0                 Ha: diff != 0                 Ha: diff > 0
 Pr(Z < z) = 0.9869         Pr(|Z| > |z|) = 0.0262          Pr(Z > z) = 0.0131

/*      TD_Mix  average Consent */

Two-sample test of proportions                     x: Number of obs =      173
                                                   y: Number of obs =      115
------------------------------------------------------------------------------
             |       Mean   Std. err.      z    P>|z|     [95% conf. interval]
-------------+----------------------------------------------------------------
           x |   .2774566   .0340413                      .2107369    .3441764
           y |   .2434783   .0400214                      .1650378    .3219187
-------------+----------------------------------------------------------------
        diff |   .0339784   .0525407                     -.0689994    .1369562
             |  under H0:   .0530282     0.64   0.522
------------------------------------------------------------------------------
        diff = prop(x) - prop(y)                                  z =   0.6408
```

```
    H0: diff = 0

    Ha: diff < 0                 Ha: diff != 0                 Ha: diff > 0
 Pr(Z < z) = 0.7392         Pr(|Z| > |z|) = 0.5217          Pr(Z > z) = 0.2608

/*      TD_BU   average Consent */

Two-sample test of proportions                     x: Number of obs =      173
                                                   y: Number of obs =      754
------------------------------------------------------------------------------
             |       Mean   Std. err.      z    P>|z|     [95% conf. interval]
-------------+----------------------------------------------------------------
           x |   .2774566   .0340413                      .2107369    .3441764
           y |   .1114058   .0114583                       .088948    .1338637
-------------+----------------------------------------------------------------
        diff |   .1660508    .035918                      .0956528    .2364488
             |  under H0:   .0294593     5.64   0.000
------------------------------------------------------------------------------
        diff = prop(x) - prop(y)                                  z =   5.6366
    H0: diff = 0

    Ha: diff < 0                 Ha: diff != 0                 Ha: diff > 0
 Pr(Z < z) = 1.0000         Pr(|Z| > |z|) = 0.0000          Pr(Z > z) = 0.0000

/*      Mix_BU  average Consent */

Two-sample test of proportions                     x: Number of obs =      115
                                                   y: Number of obs =      754
------------------------------------------------------------------------------
             |       Mean   Std. err.      z    P>|z|     [95% conf. interval]
-------------+----------------------------------------------------------------
           x |   .2434783   .0400214                      .1650378    .3219187
           y |   .1114058   .0114583                       .088948    .1338637
-------------+----------------------------------------------------------------
        diff |   .1320724   .0416293                      .0504804    .2136644
```

```
             |  under H0:   .0335438     3.94   0.000
------------------------------------------------------------------------------
        diff = prop(x) - prop(y)                                  z =   3.9373
    H0: diff = 0

    Ha: diff < 0                 Ha: diff != 0                 Ha: diff > 0
 Pr(Z < z) = 1.0000         Pr(|Z| > |z|) = 0.0001          Pr(Z > z) = 0.0000

/*      TD_Mix  above average   Consent */

Two-sample test of proportions                    x: Number of obs =      173
                                                  y: Number of obs =      115
------------------------------------------------------------------------------
             |       Mean   Std. err.      z    P>|z|     [95% conf. interval]
-------------+----------------------------------------------------------------
           x |   .2196532   .0314767                      .1579599    .2813464
           y |    .373913   .0451184                      .2854826    .4623435
-------------+----------------------------------------------------------------
        diff |  -.1542599   .0550132                     -.2620838   -.0464359
             |  under H0:   .0540953    -2.85   0.004
------------------------------------------------------------------------------
        diff = prop(x) - prop(y)                                  z =  -2.8516
    H0: diff = 0

    Ha: diff < 0                 Ha: diff != 0                 Ha: diff > 0
 Pr(Z < z) = 0.0022         Pr(|Z| > |z|) = 0.0043          Pr(Z > z) = 0.9978

/*      TD_BU   above average   Consent */

Two-sample test of proportions                    x: Number of obs =      173
                                                  y: Number of obs =      754
------------------------------------------------------------------------------
             |       Mean   Std. err.      z    P>|z|     [95% conf. interval]
-------------+----------------------------------------------------------------
           x |   .2196532   .0314767                      .1579599    .2813464
```

```
            y |   .6074271   .0177837                      .5725717    .6422824
--------------+----------------------------------------------------------------
         diff |  -.3877739   .0361531                     -.4586326   -.3169152
              |  under H0:   .0420466    -9.22   0.000
-------------------------------------------------------------------------------
        diff = prop(x) - prop(y)                                  z =  -9.2225
    H0: diff = 0

    Ha: diff < 0                 Ha: diff != 0                 Ha: diff > 0
 Pr(Z < z) = 0.0000         Pr(|Z| > |z|) = 0.0000          Pr(Z > z) = 1.0000

/*      Mix_BU  above average   Consent */

Two-sample test of proportions                     x: Number of obs =      115
                                                   y: Number of obs =      754
-------------------------------------------------------------------------------
             |       Mean   Std. err.      z    P>|z|     [95% conf. interval]
--------------+----------------------------------------------------------------
            x |    .373913   .0451184                      .2854826    .4623435
            y |   .6074271   .0177837                      .5725717    .6422824
--------------+----------------------------------------------------------------
         diff |   -.233514   .0484967                     -.3285658   -.1384622
              |  under H0:    .049465    -4.72   0.000
-------------------------------------------------------------------------------
        diff = prop(x) - prop(y)                                  z =  -4.7208
    H0: diff = 0

    Ha: diff < 0                 Ha: diff != 0                 Ha: diff > 0
 Pr(Z < z) = 0.0000         Pr(|Z| > |z|) = 0.0000          Pr(Z > z) = 1.0000

.
. **View of performance subindex
. gen perf_ind= (timely+quality+available+credibility)/4
```

```
Test scale = mean(unstandardized items)

Average interitem covariance:     .3924305
Number of items in the scale:            4
Scale reliability coefficient:      0.7363
. /* C-alpha analysis shows good internal consistency between the indicators constructing this subindex*/
.
--------------------------------------------------------------
-> govmode3 = TD

    Variable |        Obs        Mean    Std. dev.       Min        Max
-------------+---------------------------------------------------------
    perf_ind |        183     3.36612    .5986245        1.5          5

--------------------------------------------------------------
-> govmode3 = MIX

    Variable |        Obs        Mean    Std. dev.       Min        Max
-------------+---------------------------------------------------------
    perf_ind |        122    3.610656    .8849343          1          5

--------------------------------------------------------------
-> govmode3 = BU

    Variable |        Obs        Mean    Std. dev.       Min        Max
-------------+---------------------------------------------------------
    perf_ind |        729    3.624143    .7214246       1.25          5


.
---------------------------------------------------------------------------------------------------------
-----------------------------------------------------
-> govmode3 = TD

    perf_ind1 |      Freq.     Percent        Cum.
```

```
--------------+-----------------------------------
below average |         25       13.66       13.66
      average |         52       28.42       42.08
above average |        106       57.92      100.00
--------------+-----------------------------------
        Total |        183      100.00

-------------------------------------------------------------------
-> govmode3 = MIX

    perf_ind1 |      Freq.     Percent        Cum.
--------------+-----------------------------------
below average |         13       10.66       10.66
      average |         30       24.59       35.25
above average |         79       64.75      100.00
--------------+-----------------------------------
        Total |        122      100.00

-------------------------------------------------------------------
-> govmode3 = BU

    perf_ind1 |      Freq.     Percent        Cum.
--------------+-----------------------------------
below average |        109       14.95       14.95
      average |         51        7.00       21.95
above average |        569       78.05      100.00
--------------+-----------------------------------
        Total |        729      100.00


.
. *View of performance two-sample test for significancy
.
/*      TD_Mix  below average   Perf    */
```

```
Two-sample test of proportions                   x: Number of obs =      183
                                                 y: Number of obs =      122
------------------------------------------------------------------------------
             |       Mean   Std. err.      z    P>|z|     [95% conf. interval]
-------------+----------------------------------------------------------------
           x |    .136612   .0253876                      .0868532    .1863708
           y |   .1065574   .0279348                      .0518062    .1613085
-------------+----------------------------------------------------------------
        diff |   .0300546   .0377476                     -.0439293    .1040386
             |  under H0:   .0386004     0.78   0.436
------------------------------------------------------------------------------
        diff = prop(x) - prop(y)                                  z =   0.7786
    H0: diff = 0

    Ha: diff < 0                 Ha: diff != 0                 Ha: diff > 0
 Pr(Z < z) = 0.7819         Pr(|Z| > |z|) = 0.4362          Pr(Z > z) = 0.2181

/*      TD_BU   below average   Perf    */

Two-sample test of proportions                   x: Number of obs =      183
                                                 y: Number of obs =      729
------------------------------------------------------------------------------
             |       Mean   Std. err.      z    P>|z|     [95% conf. interval]
-------------+----------------------------------------------------------------
           x |    .136612   .0253876                      .0868532    .1863708
           y |   .1495199   .0132074                      .1236338    .1754059
-------------+----------------------------------------------------------------
        diff |  -.0129079   .0286176                     -.0689973    .0431816
             |  under H0:   .0292722    -0.44   0.659
------------------------------------------------------------------------------
        diff = prop(x) - prop(y)                                  z =  -0.4410
    H0: diff = 0

    Ha: diff < 0                 Ha: diff != 0                 Ha: diff > 0
 Pr(Z < z) = 0.3296         Pr(|Z| > |z|) = 0.6592          Pr(Z > z) = 0.6704
```

```
/*      Mix_BU  below average   Perf    */

Two-sample test of proportions                   x: Number of obs =      122
                                                 y: Number of obs =      729
------------------------------------------------------------------------------
             |       Mean   Std. err.      z    P>|z|     [95% conf. interval]
-------------+----------------------------------------------------------------
           x |   .1065574   .0279348                      .0518062    .1613085
           y |   .1495199   .0132074                      .1236338    .1754059
-------------+----------------------------------------------------------------
        diff |  -.0429625   .0308996                     -.1035247    .0175997
             |  under H0:   .0342796    -1.25   0.210
------------------------------------------------------------------------------
        diff = prop(x) - prop(y)                                  z =  -1.2533
    H0: diff = 0

    Ha: diff < 0                 Ha: diff != 0                 Ha: diff > 0
 Pr(Z < z) = 0.1050         Pr(|Z| > |z|) = 0.2101          Pr(Z > z) = 0.8950

 /*      TD_Mix  average Perf    */

Two-sample test of proportions                   x: Number of obs =      183
                                                 y: Number of obs =      122
------------------------------------------------------------------------------
             |       Mean   Std. err.      z    P>|z|     [95% conf. interval]
-------------+----------------------------------------------------------------
           x |    .284153   .0333396                      .2188085    .3494975
           y |   .2459016   .0389865                      .1694894    .3223139
-------------+----------------------------------------------------------------
        diff |   .0382514    .051298                     -.0622908    .1387935
             |  under H0:   .0518208     0.74   0.460
------------------------------------------------------------------------------
        diff = prop(x) - prop(y)                                  z =   0.7381
    H0: diff = 0
```

```
    Ha: diff < 0                 Ha: diff != 0                 Ha: diff > 0
 Pr(Z < z) = 0.7698         Pr(|Z| > |z|) = 0.4604          Pr(Z > z) = 0.2302

/*      TD_BU   average Perf    */

Two-sample test of proportions                     x: Number of obs =      183
                                                   y: Number of obs =      729
------------------------------------------------------------------------------
             |       Mean   Std. err.      z    P>|z|     [95% conf. interval]
-------------+----------------------------------------------------------------
           x |    .284153   .0333396                      .2188085    .3494975
           y |   .0699588   .0094473                      .0514424    .0884753
-------------+----------------------------------------------------------------
        diff |   .2141942   .0346523                      .1462769    .2821114
             |  under H0:   .0261701     8.18   0.000
------------------------------------------------------------------------------
        diff = prop(x) - prop(y)                                  z =   8.1847
    H0: diff = 0

    Ha: diff < 0                 Ha: diff != 0                 Ha: diff > 0
 Pr(Z < z) = 1.0000         Pr(|Z| > |z|) = 0.0000          Pr(Z > z) = 0.0000

 /*      Mix_BU  average Perf    */

Two-sample test of proportions                     x: Number of obs =      122
                                                   y: Number of obs =      729
------------------------------------------------------------------------------
             |       Mean   Std. err.      z    P>|z|     [95% conf. interval]
-------------+----------------------------------------------------------------
           x |   .2459016   .0389865                      .1694894    .3223139
           y |   .0699588   .0094473                      .0514424    .0884753
-------------+----------------------------------------------------------------
        diff |   .1759428   .0401149                      .0973191    .2545665
             |  under H0:   .0287064     6.13   0.000
```

```
------------------------------------------------------------------------------
        diff = prop(x) - prop(y)                                  z =   6.1290
    H0: diff = 0

    Ha: diff < 0                 Ha: diff != 0                 Ha: diff > 0
 Pr(Z < z) = 1.0000         Pr(|Z| > |z|) = 0.0000          Pr(Z > z) = 0.0000

/*      TD_Mix  above average   Perf    */

Two-sample test of proportions                     x: Number of obs =      183
                                                   y: Number of obs =      122
------------------------------------------------------------------------------
             |       Mean   Std. err.      z    P>|z|     [95% conf. interval]
-------------+----------------------------------------------------------------
           x |    .579235    .036494                       .507708    .6507619
           y |    .647541   .0432522                      .5627683    .7323137
-------------+----------------------------------------------------------------
        diff |   -.068306   .0565912                     -.1792227    .0426107
             |  under H0:    .057098    -1.20   0.232
------------------------------------------------------------------------------
        diff = prop(x) - prop(y)                                  z =  -1.1963
    H0: diff = 0

    Ha: diff < 0                 Ha: diff != 0                 Ha: diff > 0
 Pr(Z < z) = 0.1158         Pr(|Z| > |z|) = 0.2316          Pr(Z > z) = 0.8842

 /*      TD_BU   above average   Perf    */

Two-sample test of proportions                     x: Number of obs =      183
                                                   y: Number of obs =      729
------------------------------------------------------------------------------
             |       Mean   Std. err.      z    P>|z|     [95% conf. interval]
-------------+----------------------------------------------------------------
           x |    .579235    .036494                       .507708    .6507619
           y |   .7805213   .0153294                      .7504762    .8105663
```

```
-------------+----------------------------------------------------------------
        diff |  -.2012863   .0395829                     -.2788673   -.1237053
             |  under H0:    .036261    -5.55   0.000
------------------------------------------------------------------------------
        diff = prop(x) - prop(y)                                  z =  -5.5510
    H0: diff = 0

    Ha: diff < 0                 Ha: diff != 0                 Ha: diff > 0
 Pr(Z < z) = 0.0000         Pr(|Z| > |z|) = 0.0000          Pr(Z > z) = 1.0000

 /*      Mix_BU  above average   Perf    */

Two-sample test of proportions                   x: Number of obs =      122
                                                 y: Number of obs =      729
------------------------------------------------------------------------------
             |       Mean   Std. err.      z    P>|z|     [95% conf. interval]
-------------+----------------------------------------------------------------
           x |    .647541   .0432522                      .5627683    .7323137
           y |   .7805213   .0153294                      .7504762    .8105663
-------------+----------------------------------------------------------------
        diff |  -.1329803   .0458884                     -.2229198   -.0430407
             |  under H0:   .0416895    -3.19   0.001
------------------------------------------------------------------------------
        diff = prop(x) - prop(y)                                  z =  -3.1898
    H0: diff = 0

    Ha: diff < 0                 Ha: diff != 0                 Ha: diff > 0
 Pr(Z < z) = 0.0007         Pr(|Z| > |z|) = 0.0014          Pr(Z > z) = 0.9993

.
. **Legality subindex
. gen Legal_ind= (cooperation +approval +fundraise +participation +accountability+formation)/6


Test scale = mean(unstandardized items)
```

```
Average interitem covariance:     .3427388
Number of items in the scale:            6
Scale reliability coefficient:      0.7121
/* C-alpha analysis shows good internal consistency between the indicators constructing this subindex*/
.
--------------------------------------------------------------------------------------------------------
-----------------------------------------------------
-> govmode3 = TD

    Variable |        Obs        Mean    Std. dev.       Min        Max
-------------+---------------------------------------------------------
   Legal_ind |        147    2.689342    .5044096        1.5   4.333333

--------------------------------------------------------------
-> govmode3 = MIX

    Variable |        Obs        Mean    Std. dev.       Min        Max
-------------+---------------------------------------------------------
   Legal_ind |         96    2.914931    .7087567   1.333333          5

--------------------------------------------------------------
-> govmode3 = BU

    Variable |        Obs        Mean    Std. dev.       Min        Max
-------------+---------------------------------------------------------
   Legal_ind |        630    3.128307    .6981701   1.333333          5

--------------------------------------------------------------------------------------------------------
-----------------------------------------------------
-> govmode3 = TD

   Legal_ind1 |      Freq.     Percent        Cum.
--------------+-----------------------------------
below average |        102       69.39       69.39
```

```
      average |         21       14.29       83.67
above average |         24       16.33      100.00
--------------+-----------------------------------
        Total |        147      100.00

---------------------------------------------------------------
-> govmode3 = MIX

   Legal_ind1 |      Freq.     Percent        Cum.
--------------+-----------------------------------
below average |         54       56.25       56.25
      average |          9        9.38       65.62
above average |         33       34.38      100.00
--------------+-----------------------------------
        Total |         96      100.00

---------------------------------------------------------------
-> govmode3 = BU

   Legal_ind1 |      Freq.     Percent        Cum.
--------------+-----------------------------------
below average |        223       35.40       35.40
      average |         48        7.62       43.02
above average |        359       56.98      100.00
--------------+-----------------------------------
        Total |        630      100.00


. *Legality two-sample test for significancy
.
/*      TD_Mix  below average   Legality        */

Two-sample test of proportions                    x: Number of obs =      147
                                                  y: Number of obs =       96
------------------------------------------------------------------------------
```

```
             |       Mean   Std. err.      z    P>|z|     [95% conf. interval]
-------------+----------------------------------------------------------------
           x |   .6938776   .0380128                      .6193737    .7683814
           y |      .5625   .0506308                      .4632655    .6617345
-------------+----------------------------------------------------------------
        diff |   .1313776   .0633123                      .0072876    .2554675
             |  under H0:   .0629107     2.09   0.037
------------------------------------------------------------------------------
        diff = prop(x) - prop(y)                                  z =   2.0883
    H0: diff = 0

    Ha: diff < 0                 Ha: diff != 0                 Ha: diff > 0
 Pr(Z < z) = 0.9816         Pr(|Z| > |z|) = 0.0368          Pr(Z > z) = 0.0184

/*      TD_BU   below average   Legality        */

Two-sample test of proportions                     x: Number of obs =      147
                                                   y: Number of obs =      630
------------------------------------------------------------------------------
             |       Mean   Std. err.      z    P>|z|     [95% conf. interval]
-------------+----------------------------------------------------------------
           x |   .6938776   .0380128                      .6193737    .7683814
           y |   .3539683   .0190519                      .3166272    .3913093
-------------+----------------------------------------------------------------
        diff |   .3399093     .04252                      .2565716     .423247
             |  under H0:   .0451826     7.52   0.000
------------------------------------------------------------------------------
        diff = prop(x) - prop(y)                                  z =   7.5230
    H0: diff = 0

    Ha: diff < 0                 Ha: diff != 0                 Ha: diff > 0
 Pr(Z < z) = 1.0000         Pr(|Z| > |z|) = 0.0000          Pr(Z > z) = 0.0000

/*      Mix_BU  below average   Legality        */
```

```
Two-sample test of proportions                    x: Number of obs =       96
                                                  y: Number of obs =      630
------------------------------------------------------------------------------
             |       Mean   Std. err.      z    P>|z|     [95% conf. interval]
-------------+----------------------------------------------------------------
           x |      .5625   .0506308                      .4632655    .6617345
           y |   .3539683   .0190519                      .3166272    .3913093
-------------+----------------------------------------------------------------
        diff |   .2085317   .0540967                      .1025042    .3145593
             |  under H0:   .0532217     3.92   0.000
------------------------------------------------------------------------------
        diff = prop(x) - prop(y)                                  z =   3.9182
    H0: diff = 0

    Ha: diff < 0                 Ha: diff != 0                 Ha: diff > 0
 Pr(Z < z) = 1.0000         Pr(|Z| > |z|) = 0.0001          Pr(Z > z) = 0.0000

/*      TD_Mix  average Legality        */

Two-sample test of proportions                    x: Number of obs =      147
                                                  y: Number of obs =       96
------------------------------------------------------------------------------
             |       Mean   Std. err.      z    P>|z|     [95% conf. interval]
-------------+----------------------------------------------------------------
           x |   .1428571   .0288615                      .0862896    .1994246
           y |     .09375   .0297491                      .0354428    .1520572
-------------+----------------------------------------------------------------
        diff |   .0491071   .0414487                     -.0321308    .1303451
             |  under H0:   .0431671     1.14   0.255
------------------------------------------------------------------------------
        diff = prop(x) - prop(y)                                  z =   1.1376
    H0: diff = 0

    Ha: diff < 0                 Ha: diff != 0                 Ha: diff > 0
 Pr(Z < z) = 0.8724         Pr(|Z| > |z|) = 0.2553          Pr(Z > z) = 0.1276
```

```
/*      TD_BU   average Legality        */

Two-sample test of proportions                   x: Number of obs =      147
                                                 y: Number of obs =      630
------------------------------------------------------------------------------
             |       Mean   Std. err.      z    P>|z|     [95% conf. interval]
-------------+----------------------------------------------------------------
           x |   .1428571   .0288615                      .0862896    .1994246
           y |   .0761905   .0105699                      .0554738    .0969071
-------------+----------------------------------------------------------------
        diff |   .0666667   .0307361                       .006425    .1269084
             |  under H0:   .0260556     2.56   0.011
------------------------------------------------------------------------------
        diff = prop(x) - prop(y)                                  z =   2.5586
    H0: diff = 0

    Ha: diff < 0                 Ha: diff != 0                 Ha: diff > 0
 Pr(Z < z) = 0.9947         Pr(|Z| > |z|) = 0.0105          Pr(Z > z) = 0.0053

/*      Mix_BU  average Legality        */

Two-sample test of proportions                   x: Number of obs =       96
                                                 y: Number of obs =      630
------------------------------------------------------------------------------
             |       Mean   Std. err.      z    P>|z|     [95% conf. interval]
-------------+----------------------------------------------------------------
           x |     .09375   .0297491                      .0354428    .1520572
           y |   .0761905   .0105699                      .0554738    .0969071
-------------+----------------------------------------------------------------
        diff |   .0175595   .0315711                     -.0443186    .0794377
             |  under H0:   .0294697     0.60   0.551
------------------------------------------------------------------------------
        diff = prop(x) - prop(y)                                  z =   0.5958
    H0: diff = 0
```

```
    Ha: diff < 0                 Ha: diff != 0                 Ha: diff > 0
 Pr(Z < z) = 0.7244         Pr(|Z| > |z|) = 0.5513          Pr(Z > z) = 0.2756

 /*      TD_Mix  above average   Legality        */

Two-sample test of proportions                     x: Number of obs =      147
                                                   y: Number of obs =       96
------------------------------------------------------------------------------
             |       Mean   Std. err.      z    P>|z|     [95% conf. interval]
-------------+----------------------------------------------------------------
           x |   .1632653   .0304847                      .1035164    .2230143
           y |     .34375   .0484753                      .2487402    .4387598
-------------+----------------------------------------------------------------
        diff |  -.1804847   .0572641                     -.2927202   -.0682492
             |  under H0:   .0556028    -3.25   0.001
------------------------------------------------------------------------------
        diff = prop(x) - prop(y)                                  z =  -3.2460
    H0: diff = 0

    Ha: diff < 0                 Ha: diff != 0                 Ha: diff > 0
 Pr(Z < z) = 0.0006         Pr(|Z| > |z|) = 0.0012          Pr(Z > z) = 0.9994

/*      TD_BU   above average   Legality        */

Two-sample test of proportions                     x: Number of obs =      147
                                                   y: Number of obs =      630
------------------------------------------------------------------------------
             |       Mean   Std. err.      z    P>|z|     [95% conf. interval]
-------------+----------------------------------------------------------------
           x |   .1632653   .0304847                      .1035164    .2230143
           y |   .5698413   .0197252                      .5311806    .6085019
-------------+----------------------------------------------------------------
        diff |   -.406576   .0363098                     -.4777418   -.3354101
             |  under H0:   .0457939    -8.88   0.000
```

```
------------------------------------------------------------------------------
        diff = prop(x) - prop(y)                                  z =  -8.8784
    H0: diff = 0

    Ha: diff < 0                 Ha: diff != 0                 Ha: diff > 0
 Pr(Z < z) = 0.0000         Pr(|Z| > |z|) = 0.0000          Pr(Z > z) = 1.0000

/*      Mix_BU  above average   Legality        */

Two-sample test of proportions                    x: Number of obs =       96
                                                  y: Number of obs =      630
------------------------------------------------------------------------------
             |       Mean   Std. err.      z    P>|z|     [95% conf. interval]
-------------+----------------------------------------------------------------
           x |     .34375   .0484753                      .2487402    .4387598
           y |   .5698413   .0197252                      .5311806    .6085019
-------------+----------------------------------------------------------------
        diff |  -.2260913   .0523348                     -.3286657   -.1235169
             |  under H0:   .0546062    -4.14   0.000
------------------------------------------------------------------------------
        diff = prop(x) - prop(y)                                  z =  -4.1404
    H0: diff = 0

    Ha: diff < 0                 Ha: diff != 0                 Ha: diff > 0
 Pr(Z < z) = 0.0000         Pr(|Z| > |z|) = 0.0000          Pr(Z > z) = 1.0000

.
.
. **HSLI
. gen HSLI=(just_ind+consent_ind+perf_ind+Legal_ind)/4

Test scale = mean(unstandardized items)

Average interitem covariance:     .3602196
Number of items in the scale:            4
```

```
Scale reliability coefficient:      0.8291
/* C-alpha analysis shows very good internal consistency between the indicators constructing this
subindex*/


Principal components/correlation                  Number of obs    =        772
                                                  Number of comp.  =          1
                                                  Trace            =          4
    Rotation: (unrotated = principal)             Rho              =     0.6747

    --------------------------------------------------------------------------
       Component |   Eigenvalue   Difference         Proportion   Cumulative
    -------------+------------------------------------------------------------
           Comp1 |       2.6987      2.15066             0.6747       0.6747
           Comp2 |      .548039      .140031             0.1370       0.8117
           Comp3 |      .408008     .0627502             0.1020       0.9137
           Comp4 |      .345258            .             0.0863       1.0000
    --------------------------------------------------------------------------

Principal components (eigenvectors)

    --------------------------------------
        Variable |    Comp1 | Unexplained
    -------------+----------+-------------
        just_ind |   0.5062 |       .3086
     consent_ind |   0.5128 |       .2902
        perf_ind |   0.4660 |       .4139
       Legal_ind |   0.5134 |       .2886
    --------------------------------------
/*The results show that sub-indices eigenvector "loadings" on the first PC range between 0.46 and 0.51,
reflecting the correctness of using equal weights in HSLI*/

---------------------------------------------------------------------------------------------------------
-----------------------------------------------------
-> govmode3 = TD
```

Variable	Obs	Mean	Std. dev.	Min	Max
HSLI	137	2.690237	.3981791	1.416667	4

-> govmode3 = MIX

Variable	Obs	Mean	Std. dev.	Min	Max
HSLI	88	2.915483	.6340214	1.416667	4.583333

-> govmode3 = BU

Variable	Obs	Mean	Std. dev.	Min	Max
HSLI	547	3.176226	.6600903	1.3125	4.791667

-> govmode3 = TD

HSLI1	Freq.	Percent	Cum.
below average	108	78.83	78.83
average	1	0.73	79.56
above average	28	20.44	100.00
Total	137	100.00	

-> govmode3 = MIX

```
        HSLI1 |      Freq.     Percent        Cum.
--------------+-----------------------------------
below average |         56       63.64       63.64
      average |          2        2.27       65.91
above average |         30       34.09      100.00
--------------+-----------------------------------
        Total |         88      100.00

-------------------------------------------------------------------------------
-> govmode3 = BU

        HSLI1 |      Freq.     Percent        Cum.
--------------+-----------------------------------
below average |        191       34.92       34.92
      average |          8        1.46       36.38
above average |        348       63.62      100.00
--------------+-----------------------------------
        Total |        547      100.00


.
. *HSLI two-sample test for significancy
.
/*      TD_Mix  below average   HSLI    */

Two-sample test of proportions                     x: Number of obs =      137
                                                   y: Number of obs =       88
------------------------------------------------------------------------------
             |       Mean   Std. err.      z    P>|z|     [95% conf. interval]
-------------+----------------------------------------------------------------
           x |   .7883212   .0349004                      .7199177    .8567246
           y |   .6363636   .0512796                      .5358574    .7368699
-------------+----------------------------------------------------------------
        diff |   .1519575   .0620293                      .0303823    .2735328
             |  under H0:   .0607287     2.50   0.012
```

```
------------------------------------------------------------------------------
        diff = prop(x) - prop(y)                                  z =   2.5022
    H0: diff = 0

    Ha: diff < 0                 Ha: diff != 0                 Ha: diff > 0
 Pr(Z < z) = 0.9938         Pr(|Z| > |z|) = 0.0123          Pr(Z > z) = 0.0062

 /*      TD_BU   below average   HSLI    */

Two-sample test of proportions                     x: Number of obs =      137
                                                   y: Number of obs =      547
------------------------------------------------------------------------------
             |       Mean   Std. err.      z    P>|z|     [95% conf. interval]
-------------+----------------------------------------------------------------
           x |   .7883212   .0349004                      .7199177    .8567246
           y |   .3491773   .0203827                      .3092281    .3891266
-------------+----------------------------------------------------------------
        diff |   .4391438   .0404164                      .3599291    .5183586
             |  under H0:   .0473897     9.27   0.000
------------------------------------------------------------------------------
        diff = prop(x) - prop(y)                                  z =   9.2667
    H0: diff = 0

    Ha: diff < 0                 Ha: diff != 0                 Ha: diff > 0
 Pr(Z < z) = 1.0000         Pr(|Z| > |z|) = 0.0000          Pr(Z > z) = 0.0000

 /*      Mix_BU  below average   HSLI    */

Two-sample test of proportions                     x: Number of obs =       88
                                                   y: Number of obs =      547
------------------------------------------------------------------------------
             |       Mean   Std. err.      z    P>|z|     [95% conf. interval]
-------------+----------------------------------------------------------------
           x |   .6363636   .0512796                      .5358574    .7368699
           y |   .3491773   .0203827                      .3092281    .3891266
```

```
--------------+----------------------------------------------------------------
         diff |   .2871863    .055182                       .1790316    .3953411
              |  under H0:   .0559941     5.13   0.000
-------------------------------------------------------------------------------
         diff = prop(x) - prop(y)                                  z =   5.1289
    H0: diff = 0

    Ha: diff < 0                 Ha: diff != 0                 Ha: diff > 0
 Pr(Z < z) = 1.0000         Pr(|Z| > |z|) = 0.0000          Pr(Z > z) = 0.0000

/*      TD_Mix  average HSLI    */

Two-sample test of proportions                    x: Number of obs =      137
                                                  y: Number of obs =       88
-------------------------------------------------------------------------------
              |       Mean   Std. err.      z    P>|z|     [95% conf. interval]
--------------+----------------------------------------------------------------
            x |   .0072993   .0072726                      -.0069547    .0215533
            y |   .0227273   .0158869                      -.0084106    .0538651
--------------+----------------------------------------------------------------
         diff |   -.015428   .0174724                      -.0496733    .0188173
              |  under H0:   .0156691    -0.98   0.325
-------------------------------------------------------------------------------
         diff = prop(x) - prop(y)                                  z =  -0.9846
    H0: diff = 0

    Ha: diff < 0                 Ha: diff != 0                 Ha: diff > 0
 Pr(Z < z) = 0.1624         Pr(|Z| > |z|) = 0.3248          Pr(Z > z) = 0.8376

/*      TD_BU   average HSLI    */

Two-sample test of proportions                    x: Number of obs =      137
                                                  y: Number of obs =      547
-------------------------------------------------------------------------------
              |       Mean   Std. err.      z    P>|z|     [95% conf. interval]
```

```
-------------+----------------------------------------------------------------
           x |   .0072993   .0072726                      -.0069547    .0215533
           y |   .0146252   .0051328                        .004565    .0246854
-------------+----------------------------------------------------------------
        diff |   -.007326   .0089015                      -.0247726    .0101206
             |  under H0:   .0108866    -0.67   0.501
------------------------------------------------------------------------------
        diff = prop(x) - prop(y)                                  z =  -0.6729
    H0: diff = 0

    Ha: diff < 0                 Ha: diff != 0                 Ha: diff > 0
 Pr(Z < z) = 0.2505         Pr(|Z| > |z|) = 0.5010          Pr(Z > z) = 0.7495

/*      Mix_BU  average HSLI    */

Two-sample test of proportions                     x: Number of obs =       88
                                                   y: Number of obs =      547
------------------------------------------------------------------------------
             |       Mean   Std. err.      z    P>|z|     [95% conf. interval]
-------------+----------------------------------------------------------------
           x |   .0227273   .0158869                      -.0084106    .0538651
           y |   .0146252   .0051328                        .004565    .0246854
-------------+----------------------------------------------------------------
        diff |    .008102   .0166955                      -.0246206    .0408247
             |  under H0:   .0142994     0.57   0.571
------------------------------------------------------------------------------
        diff = prop(x) - prop(y)                                  z =   0.5666
    H0: diff = 0

    Ha: diff < 0                 Ha: diff != 0                 Ha: diff > 0
 Pr(Z < z) = 0.7145         Pr(|Z| > |z|) = 0.5710          Pr(Z > z) = 0.2855

 /*      TD_Mix  above average   HSLI    */

Two-sample test of proportions                     x: Number of obs =      137
```

```
                                              y: Number of obs =       88
------------------------------------------------------------------------------
             |       Mean   Std. err.      z    P>|z|     [95% conf. interval]
-------------+----------------------------------------------------------------
           x |   .2043796   .0344518                      .1368554    .2719038
           y |   .3409091   .0505302                      .2418718    .4399464
-------------+----------------------------------------------------------------
        diff |  -.1365295   .0611573                     -.2563957   -.0166633
             |  under H0:   .0597557    -2.28   0.022
------------------------------------------------------------------------------
        diff = prop(x) - prop(y)                                  z =  -2.2848
    H0: diff = 0

    Ha: diff < 0                 Ha: diff != 0                 Ha: diff > 0
 Pr(Z < z) = 0.0112         Pr(|Z| > |z|) = 0.0223          Pr(Z > z) = 0.9888

/*      TD_BU   above average   HSLI    */

Two-sample test of proportions                x: Number of obs =      137
                                              y: Number of obs =      547
------------------------------------------------------------------------------
             |       Mean   Std. err.      z    P>|z|     [95% conf. interval]
-------------+----------------------------------------------------------------
           x |   .2043796   .0344518                      .1368554    .2719038
           y |   .6361974     .02057                      .5958809     .676514
-------------+----------------------------------------------------------------
        diff |  -.4318179   .0401254                     -.5104623   -.3531735
             |  under H0:   .0475321    -9.08   0.000
------------------------------------------------------------------------------
        diff = prop(x) - prop(y)                                  z =  -9.0848
    H0: diff = 0

    Ha: diff < 0                 Ha: diff != 0                 Ha: diff > 0
 Pr(Z < z) = 0.0000         Pr(|Z| > |z|) = 0.0000          Pr(Z > z) = 1.0000
```

```
/*      Mix_BU  above average   HSLI    */

Two-sample test of proportions                   x: Number of obs =       88
                                                 y: Number of obs =      547
------------------------------------------------------------------------------
             |       Mean   Std. err.      z    P>|z|     [95% conf. interval]
-------------+----------------------------------------------------------------
           x |   .3409091   .0505302                      .2418718    .4399464
           y |   .6361974     .02057                      .5958809     .676514
-------------+----------------------------------------------------------------
        diff |  -.2952883   .0545566                     -.4022173   -.1883594
             |  under H0:   .0563755    -5.24   0.000
------------------------------------------------------------------------------
        diff = prop(x) - prop(y)                                  z =  -5.2379
    H0: diff = 0

    Ha: diff < 0                 Ha: diff != 0                 Ha: diff > 0
 Pr(Z < z) = 0.0000         Pr(|Z| > |z|) = 0.0000          Pr(Z > z) = 1.0000

.
.
. /* THE AVERAGE SCALE OF LEGITIMACY SUB-INDICES and HSLI BY THE MODE OF GOVERNANCE - THE MEAN OF ANSWERS
RANGES BETWEEN 1 AND 5 WHERE 1 IS VERY BAD*/
. /*HSLI and its sub-indices are constructed from different categorical variables, yet, their values have
wide range of possibilities thus they are considered as continuous variables*/
. /*Observations within and between groups are independent. With large sample sizes for each governance
models. anova is robust to moderate deviations from normality "Ghasemi & Zahediasl, 2012" */
. /*Pairwise comparison conducted to determine which specific groups differ from each other*/
.
. ** HSLI

/*significancy of HSLI changes across the three governance models*/

                         Number of obs =        772    R-squared     =  0.0867
                         Root MSE      =    .618776    Adj R-squared =  0.0843
```

Source	Partial SS	df	MS	F	Prob>F
Model	27.95829	2	13.979145	36.51	0.0000
govmode3	27.95829	2	13.979145	36.51	0.0000
Residual	294.43757	769	.38288371		
Total	322.39586	771	.41815287		

	Contrast	Std. err.	Unadjusted t	Unadjusted P>|t|	Unadjusted [95% conf.	interval]
govmode3						
MIX vs TD	.2252457	.0845324	2.66	0.008	.0593041	.3911873
BU vs TD	.4859892	.0591163	8.22	0.000	.3699406	.6020377
BU vs MIX	.2607434	.0710698	3.67	0.000	.1212295	.4002573

```
.
. **View of Justification

/*significancy of justification changes across the three governance models*/
```

Number of obs = 919   R-squared = 0.1863
Root MSE = .762136   Adj R-squared = 0.1846

Source	Partial SS	df	MS	F	Prob>F
Model	121.84501	2	60.922505	104.88	0.0000
govmode3	121.84501	2	60.922505	104.88	0.0000

```
                     |
            Residual |  532.05922        916   .58085068
          -----------+----------------------------------------------------
               Total |  653.90423        918   .71231398


------------------------------------------------------------------------------
             |                         Unadjusted           Unadjusted
             |   Contrast   Std. err.      t    P>|t|     [95% conf. interval]
-------------+----------------------------------------------------------------
    govmode3 |
  MIX vs TD  |   .2063942   .0925923     2.23   0.026     .0246766    .3881119
   BU vs TD  |   .8646281   .0662463    13.05   0.000      .734616    .9946403
  BU vs MIX  |   .6582339   .0774958     8.49   0.000     .5061439    .8103239
------------------------------------------------------------------------------

.
. ** Act of Consent

/*significancy of consent changes across the three governance models*/

                         Number of obs =      1,042    R-squared     =  0.0509
                         Root MSE      =     .91704    Adj R-squared =  0.0490

              Source | Partial SS         df         MS        F    Prob>F
          -----------+----------------------------------------------------
               Model |  46.819394          2   23.409697     27.84  0.0000
                     |
            govmode3 |  46.819394          2   23.409697     27.84  0.0000
                     |
            Residual |   873.7605      1,039   .84096295
          -----------+----------------------------------------------------
               Total |  920.57989      1,041   .88432267
```

	Contrast	Std. err.	Unadjusted t	Unadjusted P>|t|	Unadjusted [95% conf. interval]	
govmode3						
MIX vs TD	.1979894	.1103348	1.79	0.073	-.0185151	.414494
BU vs TD	.5391323	.0773071	6.97	0.000	.3874364	.6908282
BU vs MIX	.3411429	.0918044	3.72	0.000	.1609996	.5212862

```
.
. ** View of Performance

/*significancy of performance changes across the three governance models*/
```

Number of obs = 1,034   R-squared = 0.0180
Root MSE = .722955   Adj R-squared = 0.0161

Source	Partial SS	df	MS	F	Prob>F
Model	9.8963905	2	4.9481952	9.47	0.0001
govmode3	9.8963905	2	4.9481952	9.47	0.0001
Residual	538.86618	1,031	.52266361		
Total	548.76257	1,033	.53123192		

	Contrast	Std. err.	Unadjusted t	Unadjusted P>|t|	Unadjusted [95% conf. interval]	
govmode3						

```
  MIX vs TD  |   .2445355   .0844998     2.89   0.004     .0787243    .4103467
   BU vs TD  |   .2580224    .059775     4.32   0.000      .140728    .3753169
  BU vs MIX  |   .0134869   .0707184     0.19   0.849    -.1252814    .1522553
------------------------------------------------------------------------------

.
. ** Legality

/*significancy of legality changes across the three governance models*/

                         Number of obs =        873    R-squared     =  0.0587
                         Root MSE      =    .670794    Adj R-squared =  0.0565

                  Source | Partial SS         df         MS        F    Prob>F
              -----------+----------------------------------------------------
                   Model |  24.417851          2   12.208925     27.13  0.0000
                         |
                govmode3 |  24.417851          2   12.208925     27.13  0.0000
                         |
                Residual |  391.46931        870   .44996472
              -----------+----------------------------------------------------
                   Total |  415.88716        872   .47693481



------------------------------------------------------------------------------
             |                            Unadjusted           Unadjusted
             |   Contrast   Std. err.      t    P>|t|     [95% conf. interval]
-------------+----------------------------------------------------------------
    govmode3 |
  MIX vs TD  |   .2255882   .0880234     2.56   0.011     .0528251    .3983512
   BU vs TD  |   .4389645   .0614428     7.14   0.000     .3183711    .5595579
  BU vs MIX  |   .2133763    .073494     2.90   0.004     .0691301    .3576225
------------------------------------------------------------------------------
```

```
.
.
. /*Multinomial logistic regression to assess statistically significant differences (P<0.05) among the
mean of the categorical indicators across different governance models*/
.
. * Transparency

Multinomial logistic regression                         Number of obs =  1,023
                                                        LR chi2(8)    =  39.13
                                                        Prob > chi2   = 0.0000
Log likelihood = -1242.7199                             Pseudo R2     = 0.0155

------------------------------------------------------------------------------
transparency | Coefficient  Std. err.      z    P>|z|     [95% conf. interval]
-------------+----------------------------------------------------------------
v_bad        |  (base outcome)
-------------+----------------------------------------------------------------
bad          |
    govmode3 |
        MIX  |   .4191154   .2754193     1.52   0.128    -.1206965    .9589274
         BU  |   .8209744   .2029134     4.05   0.000     .4232714    1.218677
             |
       _cons |  -.9404465   .1841595    -5.11   0.000    -1.301392   -.5795005
-------------+----------------------------------------------------------------
average      |
    govmode3 |
        MIX  |   .3040743   .3482253     0.87   0.383    -.3784347    .9865832
         BU  |   .5205068   .2562741     2.03   0.042     .0182188    1.022795
             |
       _cons |   -1.51854   .2302262    -6.60   0.000    -1.969775   -1.067305
-------------+----------------------------------------------------------------
good         |
    govmode3 |
        MIX  |  -13.87063   537.5327    -0.03   0.979    -1067.415    1039.674
         BU  |    1.03229   .4481995     2.30   0.021      .153835    1.910745
```

_cons	-2.862247	.419755	-6.82	0.000	-3.684952	-2.039543
v_good						
govmode3						
MIX	1.187752	1.234787	0.96	0.336	-1.232386	3.60789
BU	1.745984	1.03681	1.68	0.092	-.2861257	3.778094
_cons	-4.653523	1.004522	-4.63	0.000	-6.622349	-2.684696

Multinomial logistic regression

Number of obs = 299
LR chi2(4) = 9.76
Prob > chi2 = 0.0448
Log likelihood = -316.38722
Pseudo R2 = 0.0152

transparency	Coefficient	Std. err.	z	P>|z|	[95% conf. interval]	
v_bad	(base outcome)					
bad						
2.MB	.4190949	.2754179	1.52	0.128	-.1207143	.9589041
_cons	-.9403828	.1841582	-5.11	0.000	-1.301326	-.5794394
average						
2.MB	.3040223	.3482232	0.87	0.383	-.3784827	.9865273
_cons	-1.518465	.2302228	-6.60	0.000	-1.969694	-1.067237
good						
2.MB	-14.66858	800.9904	-0.02	0.985	-1584.581	1555.244
_cons	-2.861994	.4197115	-6.82	0.000	-3.684614	-2.039375
v_good						

```
        2.MB |   1.188231   1.234877     0.96   0.336    -1.232083    3.608545
       _cons |  -4.653802   1.004677    -4.63   0.000    -6.622932   -2.684672
------------------------------------------------------------------------------

.
. * Fairness

Multinomial logistic regression                         Number of obs =  1,051
                                                        LR chi2(8)    = 190.47
                                                        Prob > chi2   = 0.0000
Log likelihood = -1504.7806                             Pseudo R2     = 0.0595

------------------------------------------------------------------------------
     justice | Coefficient  Std. err.      z    P>|z|     [95% conf. interval]
-------------+----------------------------------------------------------------
v_bad        |  (base outcome)
-------------+----------------------------------------------------------------
bad          |
    govmode3 |
        MIX  |   .6144815   .2724162     2.26   0.024     .0805555    1.148408
         BU  |   1.223036   .2111661     5.79   0.000     .8091581    1.636914
             |
       _cons |  -.9308188   .1844062    -5.05   0.000    -1.292248   -.5693894
-------------+----------------------------------------------------------------
average      |
    govmode3 |
        MIX  |   .2667489   .3370483     0.79   0.429    -.3938537    .9273515
         BU  |    .827576   .2508328     3.30   0.001     .3359527    1.319199
             |
       _cons |  -1.348554   .2159917    -6.24   0.000     -1.77189    -.925218
-------------+----------------------------------------------------------------
good         |
    govmode3 |
        MIX  |  -.5317588   .8326051    -0.64   0.523    -2.163635    1.100117
         BU  |   2.763966   .4347013     6.36   0.000     1.911967    3.615965
```

	Coefficient	Std. err.	z	P>|z|	[95% conf. interval]	
_cons	-2.852631	.4198596	-6.79	0.000	-3.675541	-2.029722
v_good						
govmode3						
MIX	.5668241	1.423563	0.40	0.691	-2.223308	3.356956
BU	4.256913	1.012212	4.21	0.000	2.273014	6.240812
_cons	-4.644359	1.004781	-4.62	0.000	-6.613693	-2.675026

Multinomial logistic regression

Number of obs = 304
LR chi2(4) = 6.13
Prob > chi2 = 0.1896
Log likelihood = -333.46276
Pseudo R2 = 0.0091

justice	Coefficient	Std. err.	z	P>|z|	[95% conf. interval]	
v_bad	(base outcome)					
bad						
2.MB	.6144815	.2724162	2.26	0.024	.0805555	1.148407
_cons	-.9308188	.1844062	-5.05	0.000	-1.292248	-.5693894
average						
2.MB	.2667489	.3370483	0.79	0.429	-.3938537	.9273515
_cons	-1.348554	.2159917	-6.24	0.000	-1.77189	-.925218
good						
2.MB	-.5317513	.8326028	-0.64	0.523	-2.163623	1.10012
_cons	-2.852631	.4198596	-6.79	0.000	-3.675541	-2.029722
v_good						

```
        2.MB |   .5668535   1.423575     0.40   0.690    -2.223302    3.357008
       _cons |  -4.644391   1.004796    -4.62   0.000    -6.613755   -2.675027
------------------------------------------------------------------------------

.
. *Incorruptness

Multinomial logistic regression                         Number of obs =    982
                                                        LR chi2(8)    = 168.02
                                                        Prob > chi2   = 0.0000
Log likelihood = -1360.2329                             Pseudo R2     = 0.0582

------------------------------------------------------------------------------
     corrupt | Coefficient  Std. err.      z    P>|z|     [95% conf. interval]
-------------+----------------------------------------------------------------
v_bad        |  (base outcome)
-------------+----------------------------------------------------------------
bad          |
    govmode3 |
        MIX  |   .7002824   .2754383     2.54   0.011     .1604331    1.240132
         BU  |   1.423994   .2125278     6.70   0.000     1.007447    1.840541
             |
       _cons |  -1.029825   .1842061    -5.59   0.000    -1.390862   -.6687875
-------------+----------------------------------------------------------------
average      |
    govmode3 |
        MIX  |   .7867273   .3713922     2.12   0.034      .058812    1.514643
         BU  |   1.764217   .2864306     6.16   0.000     1.202823    2.325611
             |
       _cons |  -1.885404   .2602987    -7.24   0.000     -2.39558   -1.375228
-------------+----------------------------------------------------------------
good         |
    govmode3 |
        MIX  |  -.0177196   .6335864    -0.03   0.978    -1.259526    1.224087
         BU  |   2.455291   .3855993     6.37   0.000     1.699531    3.211052
```

_cons	-2.638976	.3659387	-7.21	0.000	-3.356202	-1.921749
**v_good**						
govmode3						
MIX	.4521692	1800.236	0.00	1.000	-3527.947	3528.851
BU	17.95538	1200.105	0.01	0.988	-2334.206	2370.117
_cons	-18.89888	1200.104	-0.02	0.987	-2371.06	2333.263

Multinomial logistic regression

Number of obs = 298
LR chi2(3) = 9.13
Prob > chi2 = 0.0277
Pseudo R2 = 0.0144

Log likelihood = -311.44205

corrupt	Coefficient	Std. err.	z	P>|z|	[95% conf.	interval]
**v_bad**	(base outcome)					
**bad**						
2.MB	.7001402	.2754318	2.54	0.011	.1603038	1.239977
_cons	-1.029619	.1841971	-5.59	0.000	-1.390639	-.6685997
**average**						
2.MB	.7866732	.371386	2.12	0.034	.0587701	1.514576
_cons	-1.885286	.2602923	-7.24	0.000	-2.395449	-1.375122
**good**						
2.MB	-.0176996	.6336185	-0.03	0.978	-1.259569	1.22417
_cons	-2.639057	.3659625	-7.21	0.000	-3.356331	-1.921784

```
.
. *Understand needs

Multinomial logistic regression                         Number of obs =  1,077
                                                        LR chi2(8)    = 145.76
                                                        Prob > chi2   = 0.0000
Log likelihood = -1575.7316                             Pseudo R2     = 0.0442

------------------------------------------------------------------------------
    capacity | Coefficient  Std. err.      z    P>|z|     [95% conf. interval]
-------------+----------------------------------------------------------------
v_bad        |
    govmode3 |
        MIX  |  -.0949455    .346461    -0.27   0.784    -.7739967    .5841056
         BU  |  -.2437628    .259349    -0.94   0.347    -.7520776    .2645519
             |
       _cons |  -.9219887   .1998376    -4.61   0.000    -1.313663   -.5303142
-------------+----------------------------------------------------------------
bad          |  (base outcome)
-------------+----------------------------------------------------------------
average      |
    govmode3 |
        MIX  |   .2671865   .2987129     0.89   0.371    -.3182801    .8526531
         BU  |   1.021518   .2142374     4.77   0.000     .6016208    1.441416
             |
       _cons |  -.7161367   .1860631    -3.85   0.000    -1.080814   -.3514598
-------------+----------------------------------------------------------------
good         |
    govmode3 |
        MIX  |   1.073476   .3676143     2.92   0.003     .3529655    1.793987
         BU  |   2.114533   .2909768     7.27   0.000     1.544229    2.684837
             |
       _cons |  -1.704748   .2717787    -6.27   0.000    -2.237425   -1.172072
-------------+----------------------------------------------------------------
v_good       |
```

govmode3						
MIX	.4040495	.6947182	0.58	0.561	-.9575732	1.765672
BU	2.651214	.4753094	5.58	0.000	1.719625	3.582804
_cons	-2.867885	.4597401	-6.24	0.000	-3.768959	-1.966811

Multinomial logistic regression

Number of obs = 310
LR chi2(4) = 10.13
Prob > chi2 = 0.0382
Log likelihood = -420.35839
Pseudo R2 = 0.0119

capacity	Coefficient	Std. err.	z	P>|z|	[95% conf.	interval]
v_bad						
2.MB	-.0949455	.346461	-0.27	0.784	-.7739965	.5841055
_cons	-.9219888	.1998376	-4.61	0.000	-1.313663	-.5303143
bad	(base outcome)					
average						
2.MB	.2671865	.2987129	0.89	0.371	-.31828	.8526529
_cons	-.7161367	.186063	-3.85	0.000	-1.080814	-.3514599
good						
2.MB	1.073476	.3676142	2.92	0.003	.3529656	1.793987
_cons	-1.704748	.2717787	-6.27	0.000	-2.237424	-1.172072
v_good						
2.MB	.4040457	.6947231	0.58	0.561	-.9575867	1.765678
_cons	-2.867899	.459743	-6.24	0.000	-3.768979	-1.966819

.

. * Impartiality

Multinomial logistic regression

Number of obs	= 1,076
LR chi2(8)	= 328.48
Prob > chi2	= 0.0000
Pseudo R2	= 0.1060

Log likelihood = -1384.8441

neutral	Coefficient	Std. err.	z	P>|z|	[95% conf.	interval]
v_bad						
govmode3						
MIX	-1.116961	.6598133	-1.69	0.090	-2.410172	.1762488
BU	-3.777249	.5284876	-7.15	0.000	-4.813065	-2.741432
_cons	.9162907	.4830459	1.90	0.058	-.0304618	1.863043
bad						
govmode3						
MIX	-.701446	.5525423	-1.27	0.204	-1.784409	.3815171
BU	-3.546231	.4512718	-7.86	0.000	-4.430707	-2.661755
_cons	1.99243	.4351941	4.58	0.000	1.139465	2.845395
average						
govmode3						
MIX	-1.631989	.5415953	-3.01	0.003	-2.693496	-.5704814
BU	-4.167651	.4332195	-9.62	0.000	-5.016746	-3.318557
_cons	2.87168	.4196435	6.84	0.000	2.049194	3.694166
good						
govmode3						
MIX	-.183279	.5871031	-0.31	0.755	-1.33398	.9674221
BU	-2.214658	.4785186	-4.63	0.000	-3.152537	-1.276778

_cons	1.15268	.4682929	2.46	0.014	.2348423	2.070517
v_good	(base outcome)					

Multinomial logistic regression

Number of obs	= 317
LR chi2(4)	= 26.12
Prob > chi2	= 0.0000
Pseudo R2	= 0.0306

Log likelihood = -414.41489

neutral	Coefficient	Std. err.	z	P>|z|	[95% conf.	interval]
v_bad						
2.MB	.5150273	.4620904	1.11	0.265	-.3906532	1.420708
_cons	-1.955389	.2758634	-7.09	0.000	-2.496071	-1.414707
bad						
2.MB	.9305428	.2889239	3.22	0.001	.3642623	1.496823
_cons	-.8792495	.1793355	-4.90	0.000	-1.230741	-.5277583
average	(base outcome)					
good						
2.MB	1.44871	.3505197	4.13	0.000	.7617037	2.135716
_cons	-1.719	.2491296	-6.90	0.000	-2.207285	-1.230715
v_good						
2.MB	1.631989	.5415953	3.01	0.003	.5704814	2.693496
_cons	-2.87168	.4196435	-6.84	0.000	-3.694166	-2.049194

.
. * Traditions

```
Multinomial logistic regression                         Number of obs =  1,081
                                                        LR chi2(8)    = 227.75
                                                        Prob > chi2   = 0.0000
Log likelihood = -1271.1409                             Pseudo R2     = 0.0822

------------------------------------------------------------------------------
   tradition | Coefficient  Std. err.      z    P>|z|     [95% conf. interval]
-------------+----------------------------------------------------------------
v_bad        |
    govmode3 |
        MIX  |  -.8823892   1.255448    -0.70   0.482    -3.343022    1.578243
         BU  |  -1.141654   .7919705    -1.44   0.149    -2.693888    .4105796
             |
       _cons |  -2.484907   .7359801    -3.38   0.001    -3.927401   -1.042412
-------------+----------------------------------------------------------------
bad          |
    govmode3 |
        MIX  |  -.7953778   .5776895    -1.38   0.169    -1.927628    .3368728
         BU  |  -1.111801   .3866509    -2.88   0.004    -1.869623   -.3539792
             |
       _cons |  -.7801585   .3641095    -2.14   0.032      -1.4938    -.066517
-------------+----------------------------------------------------------------
average      |
    govmode3 |
        MIX  |   -.967547   .3295083    -2.94   0.003    -1.613371   -.3217226
         BU  |  -2.827033   .2515201   -11.24   0.000    -3.320003   -2.334063
             |
       _cons |   1.406914   .2277515     6.18   0.000     .9605289    1.853298
-------------+----------------------------------------------------------------
good         |
    govmode3 |
        MIX  |  -.4036519   .3397535    -1.19   0.235    -1.069556    .2622527
         BU  |  -2.172444   .2638646    -8.23   0.000    -2.689609   -1.655279
             |
```

```
       _cons |   .8649974   .2433321     3.55   0.000     .3880752     1.34192
-------------+----------------------------------------------------------------
v_good       |  (base outcome)
------------------------------------------------------------------------------


Multinomial logistic regression                         Number of obs =    319
                                                        LR chi2(4)    =  10.26
                                                        Prob > chi2   = 0.0362
Log likelihood = -385.01425                             Pseudo R2     = 0.0132

------------------------------------------------------------------------------
   tradition | Coefficient  Std. err.      z    P>|z|     [95% conf. interval]
-------------+----------------------------------------------------------------
v_bad        |
        2.MB |   .0851578   1.237912     0.07   0.945    -2.341105    2.511421
       _cons |   -3.89182   .7142857    -5.45   0.000    -5.291795   -2.491846
-------------+----------------------------------------------------------------
bad          |
        2.MB |   .1721692   .5385184     0.32   0.749    -.8833075    1.227646
       _cons |  -2.187072    .317983    -6.88   0.000    -2.810307   -1.563837
-------------+----------------------------------------------------------------
average      |  (base outcome)
-------------+----------------------------------------------------------------
good         |
        2.MB |   .5638951   .2677859     2.11   0.035     .0390444    1.088746
       _cons |  -.5419162   .1665771    -3.25   0.001    -.8684014    -.215431
-------------+----------------------------------------------------------------
v_good       |
        2.MB |    .967547   .3295083     2.94   0.003     .3217226    1.613371
       _cons |  -1.406914   .2277515    -6.18   0.000    -1.853298   -.9605289
------------------------------------------------------------------------------

.
. * Compliance
```

Multinomial logistic regression

Number of obs	= 1,079
LR chi2(8)	= 21.06
Prob > chi2	= 0.0070
Pseudo R2	= 0.0070

Log likelihood = -1487.5617

commit	Coefficient	Std. err.	z	P>|z|	[95% conf.	interval]
v_bad						
govmode3						
MIX	.2613647	.6091297	0.43	0.668	-.9325075	1.455237
BU	.2790018	.4414492	0.63	0.527	-.5862227	1.144226
_cons	-2.65926	.3909695	-6.80	0.000	-3.425546	-1.892974
bad						
govmode3						
MIX	.5406786	.3775724	1.43	0.152	-.1993497	1.280707
BU	.7208347	.2805881	2.57	0.010	.1708922	1.270777
_cons	-1.714798	.2560382	-6.70	0.000	-2.216624	-1.212973
average	(base outcome)					
good						
govmode3						
MIX	.2047944	.3003645	0.68	0.495	-.3839092	.7934981
BU	.7543996	.2067434	3.65	0.000	.3491901	1.159609
_cons	-.9162907	.1870829	-4.90	0.000	-1.282966	-.549615
v_good						
govmode3						
MIX	.4700036	.3370999	1.39	0.163	-.1907001	1.130707

```
          BU  |   .4753275   .2498165     1.90   0.057    -.0143038    .9649588
              |
       _cons |  -1.386294   .2236068    -6.20   0.000    -1.824556   -.9480331
------------------------------------------------------------------------------

Multinomial logistic regression                         Number of obs =    316
                                                        LR chi2(4)    =   3.30
                                                        Prob > chi2   = 0.5096
Log likelihood = -418.50827                             Pseudo R2     = 0.0039

------------------------------------------------------------------------------
      commit | Coefficient  Std. err.      z    P>|z|     [95% conf. interval]
-------------+----------------------------------------------------------------
v_bad        |
        2.MB |   .2613648   .6091297     0.43   0.668    -.9325075    1.455237
       _cons |   -2.65926   .3909695    -6.80   0.000    -3.425546   -1.892974
-------------+----------------------------------------------------------------
bad          |
        2.MB |   .5406786   .3775724     1.43   0.152    -.1993497    1.280707
       _cons |  -1.714798   .2560382    -6.70   0.000    -2.216624   -1.212973
-------------+----------------------------------------------------------------
average      |  (base outcome)
-------------+----------------------------------------------------------------
good         |
        2.MB |   .2047944   .3003645     0.68   0.495    -.3839093    .7934981
       _cons |  -.9162907   .1870829    -4.90   0.000    -1.282966    -.549615
-------------+----------------------------------------------------------------
v_good       |
        2.MB |   .4700036   .3370999     1.39   0.163    -.1907001    1.130707
       _cons |  -1.386294   .2236068    -6.20   0.000    -1.824556   -.9480331
------------------------------------------------------------------------------

.
. * Delegation
```

Multinomial logistic regression

Number of obs	= 1,049
LR chi2(8)	= 145.14
Prob > chi2	= 0.0000
Pseudo R2	= 0.0441

Log likelihood = −1571.9613

represent	Coefficient	Std. err.	z	P>|z|	[95% conf.	interval]
v_bad						
govmode3						
MIX	−.6017974	.4190717	−1.44	0.151	−1.423163	.2195681
BU	−.226681	.2898135	−0.78	0.434	−.794705	.3413429
_cons	−.6021754	.2593989	−2.32	0.020	−1.110588	−.0937629
bad						
govmode3						
MIX	−.7535563	.2931634	−2.57	0.010	−1.328146	−.1789665
BU	−1.301912	.2222362	−5.86	0.000	−1.737487	−.8663371
_cons	.7282385	.187893	3.88	0.000	.359975	1.096502
average	(base outcome)					
good						
govmode3						
MIX	−.076373	.4174922	−0.18	0.855	−.8946426	.7418967
BU	1.005748	.3038032	3.31	0.001	.4103044	1.601191
_cons	−.9044563	.2874597	−3.15	0.002	−1.467867	−.3410457
v_good						
govmode3						
MIX	.4542538	.571478	0.79	0.427	−.6658224	1.57433
BU	1.653138	.4498387	3.67	0.000	.77147	2.534805

```
             |
       _cons |  -1.945909   .4364355    -4.46   0.000    -2.801307   -1.090511
------------------------------------------------------------------------------


Multinomial logistic regression                         Number of obs =    290
                                                        LR chi2(4)    =  10.55
                                                        Prob > chi2   = 0.0322
Log likelihood = -392.30832                             Pseudo R2     = 0.0133

------------------------------------------------------------------------------
   represent | Coefficient  Std. err.      z    P>|z|     [95% conf. interval]
-------------+----------------------------------------------------------------
v_bad        |
        2.MB |   .1517589   .4049036     0.37   0.708    -.6418375    .9453553
       _cons |  -1.330414   .2344622    -5.67   0.000    -1.789951   -.8708765
-------------+----------------------------------------------------------------
bad          |  (base outcome)
-------------+----------------------------------------------------------------
average      |
        2.MB |   .7535563   .2931635     2.57   0.010     .1789665    1.328146
       _cons |  -.7282385    .187893    -3.88   0.000    -1.096502    -.359975
-------------+----------------------------------------------------------------
good         |
        2.MB |   .6771833   .4032685     1.68   0.093    -.1132085    1.467575
       _cons |  -1.632695    .265175    -6.16   0.000    -2.152428   -1.112961
-------------+----------------------------------------------------------------
v_good       |
        2.MB |   1.207812   .5611707     2.15   0.031     .1079379    2.307687
       _cons |  -2.674147   .4220908    -6.34   0.000     -3.50143   -1.846864
------------------------------------------------------------------------------

.
. * Responsiveness
.
```

Multinomial logistic regression

Number of obs	= 1,083
LR chi2(8)	= 104.83
Prob > chi2	= 0.0000
Pseudo R2	= 0.0352

Log likelihood = -1435.8297

timely	Coefficient	Std. err.	z	P>|z|	[95% conf.	interval]
v_bad						
govmode3						
MIX	-.1000834	.8195604	-0.12	0.903	-1.706392	1.506225
BU	-.8622235	.659128	-1.31	0.191	-2.154091	.4296437
_cons	-1.845827	.6212607	-2.97	0.003	-3.063475	-.6281781
bad						
govmode3						
MIX	-.8230836	.4879329	-1.69	0.092	-1.779414	.1332473
BU	-1.627424	.3629419	-4.48	0.000	-2.338777	-.916071
_cons	-.1112257	.3338489	-0.33	0.739	-.7655576	.5431062
average						
govmode3						
MIX	-1.180996	.3557617	-3.32	0.001	-1.878276	-.4837159
BU	-2.143807	.2709513	-7.91	0.000	-2.674862	-1.612752
_cons	1.486378	.25404	5.85	0.000	.9884685	1.984287
good						
govmode3						
MIX	-.7786318	.3532575	-2.20	0.028	-1.471004	-.0862598
BU	-1.859226	.2753226	-6.75	0.000	-2.398848	-1.319603
_cons	1.275069	.2594946	4.91	0.000	.7664686	1.783669

```
-------------+----------------------------------------------------------------
v_good       |  (base outcome)
------------------------------------------------------------------------------

Multinomial logistic regression                         Number of obs =    318
                                                        LR chi2(4)    =  12.26
                                                        Prob > chi2   = 0.0155
Log likelihood = -412.30652                             Pseudo R2     = 0.0146

------------------------------------------------------------------------------
      timely | Coefficient  Std. err.      z    P>|z|     [95% conf. interval]
-------------+----------------------------------------------------------------
v_bad        |
        2.MB |   1.080913   .7883869     1.37   0.170    -.4642972    2.626123
       _cons |  -3.332205   .5875697    -5.67   0.000     -4.48382   -2.180589
-------------+----------------------------------------------------------------
bad          |
        2.MB |   .3579126   .4335357     0.83   0.409    -.4918017    1.207627
       _cons |  -1.597603   .2659479    -6.01   0.000    -2.118852   -1.076355
-------------+----------------------------------------------------------------
average      |  (base outcome)
-------------+----------------------------------------------------------------
good         |
        2.MB |   .4023643     .27325     1.47   0.141    -.1331958    .9379245
       _cons |  -.2113091   .1631277    -1.30   0.195    -.5310335    .1084153
-------------+----------------------------------------------------------------
v_good       |
        2.MB |   1.180996   .3557617     3.32   0.001      .483716    1.878276
       _cons |  -1.486378     .25404    -5.85   0.000    -1.984287   -.9884685
------------------------------------------------------------------------------

.
. * Quality

Multinomial logistic regression                         Number of obs =  1,073
```

LR chi2(8) = 70.49
Prob > chi2 = 0.0000
Log likelihood = −1388.4593 Pseudo R2 = 0.0248

quality	Coefficient	Std. err.	z	P>|z|	[95% conf.	interval]
v_bad						
govmode3						
MIX	1.313593	.8606879	1.53	0.127	−.3733243	3.00051
BU	.1151784	.7814392	0.15	0.883	−1.416414	1.646771
_cons	−3.417727	.7186053	−4.76	0.000	−4.826167	−2.009286
bad						
govmode3						
MIX	−.2005352	.4262544	−0.47	0.638	−1.035978	.634908
BU	−.5077645	.2944267	−1.72	0.085	−1.08483	.0693012
_cons	−1.115142	.2576693	−4.33	0.000	−1.620164	−.6101191
average						
govmode3						
MIX	−.4012059	.2701961	−1.48	0.138	−.9307805	.1283687
BU	−.8287639	.1855016	−4.47	0.000	−1.19234	−.4651875
_cons	.4942963	.1624606	3.04	0.002	.1758794	.8127133
good	(base outcome)					
v_good						
govmode3						
MIX	1.453347	.4574507	3.18	0.001	.5567606	2.349934
BU	1.490037	.3879229	3.84	0.000	.729722	2.250352

```
       _cons |  -2.031425   .3760221    -5.40   0.000    -2.768415   -1.294436
------------------------------------------------------------------------------


Multinomial logistic regression                         Number of obs =    316
                                                        LR chi2(4)    =  23.00
                                                        Prob > chi2   = 0.0001
Log likelihood = -387.40997                             Pseudo R2     = 0.0288

------------------------------------------------------------------------------
     quality | Coefficient  Std. err.      z    P>|z|     [95% conf. interval]
-------------+----------------------------------------------------------------
v_bad        |
        2.MB |   1.714798   .8556998     2.00   0.045     .0376576    3.391939
       _cons |  -3.912023   .7141428    -5.48   0.000    -5.311717   -2.512329
-------------+----------------------------------------------------------------
bad          |
        2.MB |   .2006707   .4160905     0.48   0.630    -.6148517    1.016193
       _cons |  -1.609438    .244949    -6.57   0.000    -2.089529   -1.129347
-------------+----------------------------------------------------------------
average      |  (base outcome)
-------------+----------------------------------------------------------------
good         |
        2.MB |   .4012059   .2701961     1.48   0.138    -.1283686    .9307804
       _cons |  -.4942963   .1624606    -3.04   0.002    -.8127132   -.1758794
-------------+----------------------------------------------------------------
v_good       |
        2.MB |    1.85456   .4479961     4.14   0.000     .9765042    2.732617
       _cons |  -2.525729   .3674235    -6.87   0.000    -3.245865   -1.805592
------------------------------------------------------------------------------

.
. * Availability

Multinomial logistic regression                         Number of obs =  1,083
```

LR chi2(8) = 80.14
Prob > chi2 = 0.0000
Log likelihood = -1438.7479   Pseudo R2 = 0.0271

available	Coefficient	Std. err.	z	P>|z|	[95% conf. interval]	
v_bad						
govmode3						
MIX	.9808293	.7843776	1.25	0.211	-.5565226	2.518181
BU	.8598649	.6250829	1.38	0.169	-.365275	2.085005
_cons	-3.444682	.5864909	-5.87	0.000	-4.594184	-2.295181
bad						
govmode3						
MIX	.4700036	.5068677	0.93	0.354	-.5234389	1.463446
BU	1.410747	.3484764	4.05	0.000	.7277459	2.093748
_cons	-2.24071	.3326234	-6.74	0.000	-2.892639	-1.58878
average	(base outcome)					
good						
govmode3						
MIX	.0363676	.2609025	0.14	0.889	-.4749918	.5477271
BU	-.3244421	.1775551	-1.83	0.068	-.6724437	.0235596
_cons	-.1488456	.1516047	-0.98	0.326	-.4459853	.1482941
v_good						
govmode3						
MIX	2.525729	.5673754	4.45	0.000	1.413693	3.637764
BU	2.172887	.5221962	4.16	0.000	1.149401	3.196373

```
       _cons |     -3.157   .5105275    -6.18   0.000    -4.157616   -2.156385
------------------------------------------------------------------------------


Multinomial logistic regression                         Number of obs =    318
                                                        LR chi2(4)    =  32.05
                                                        Prob > chi2   = 0.0000
Log likelihood = -363.33201                             Pseudo R2     = 0.0422

------------------------------------------------------------------------------
   available | Coefficient  Std. err.      z    P>|z|     [95% conf. interval]
-------------+----------------------------------------------------------------
v_bad        |
        2.MB |   .9808293   .7843776     1.25   0.211    -.5565226    2.518181
       _cons |  -3.444682   .5864909    -5.87   0.000    -4.594184   -2.295181
-------------+----------------------------------------------------------------
bad          |
        2.MB |   .4700036   .5068677     0.93   0.354    -.5234389    1.463446
       _cons |   -2.24071   .3326234    -6.74   0.000    -2.892639    -1.58878
-------------+----------------------------------------------------------------
average      |  (base outcome)
-------------+----------------------------------------------------------------
good         |
        2.MB |   .0363676   .2609025     0.14   0.889    -.4749918    .5477271
       _cons |  -.1488456   .1516047    -0.98   0.326    -.4459853    .1482941
-------------+----------------------------------------------------------------
v_good       |
        2.MB |   2.525729   .5673754     4.45   0.000     1.413693    3.637764
       _cons |     -3.157   .5105275    -6.18   0.000    -4.157616   -2.156385
------------------------------------------------------------------------------

.
. * Reliability

Multinomial logistic regression                         Number of obs =  1,052
```

LR chi2(8) = 35.83
Prob > chi2 = 0.0000
Log likelihood = -1363.0432 Pseudo R2 = 0.0130

credibility	Coefficient	Std. err.	z	P>|z|	[95% conf.	interval]
v_bad						
govmode3						
MIX	.6407792	1.015461	0.63	0.528	-1.349488	2.631046
BU	.9829821	.762398	1.29	0.197	-.5112906	2.477255
_cons	-3.839452	.7146696	-5.37	0.000	-5.240179	-2.438726
bad						
govmode3						
MIX	.3996171	.4398847	0.91	0.364	-.462541	1.261775
BU	.6728272	.3146422	2.14	0.032	.0561399	1.289514
_cons	-1.893542	.2866727	-6.61	0.000	-2.45541	-1.331674
average	(base outcome)					
good						
govmode3						
MIX	.0156855	.2675092	0.06	0.953	-.5086228	.5399938
BU	.2963862	.1799999	1.65	0.100	-.0564071	.6491795
_cons	-.2699196	.1575982	-1.71	0.087	-.5788064	.0389672
v_good						
govmode3						
MIX	1.872923	.4644186	4.03	0.000	.962679	2.783166
BU	1.80966	.4071542	4.44	0.000	1.011653	2.607668

```
       _cons |  -2.586689   .3919309    -6.60   0.000     -3.35486   -1.818519
------------------------------------------------------------------------------


Multinomial logistic regression                         Number of obs =    311
                                                        LR chi2(4)    =  21.38
                                                        Prob > chi2   = 0.0003
Log likelihood = -366.83183                             Pseudo R2     = 0.0283

------------------------------------------------------------------------------
 credibility | Coefficient  Std. err.      z    P>|z|     [95% conf. interval]
-------------+----------------------------------------------------------------
v_bad        |
        2.MB |   .6407792   1.015461     0.63   0.528    -1.349488    2.631046
       _cons |  -3.839452   .7146696    -5.37   0.000    -5.240179   -2.438726
-------------+----------------------------------------------------------------
bad          |
        2.MB |   .3996171   .4398847     0.91   0.364     -.462541    1.261775
       _cons |  -1.893542   .2866727    -6.61   0.000     -2.45541   -1.331674
-------------+----------------------------------------------------------------
average      |  (base outcome)
-------------+----------------------------------------------------------------
good         |
        2.MB |   .0156855   .2675092     0.06   0.953    -.5086228    .5399938
       _cons |  -.2699196   .1575982    -1.71   0.087    -.5788064    .0389672
-------------+----------------------------------------------------------------
v_good       |
        2.MB |   1.872923   .4644186     4.03   0.000     .9626791    2.783167
       _cons |  -2.586689   .3919309    -6.60   0.000     -3.35486   -1.818519
------------------------------------------------------------------------------

.
. * Coordination

Multinomial logistic regression                         Number of obs =  1,040
```

	LR chi2(8)	= 145.31
	Prob > chi2	= 0.0000
Log likelihood = −1471.2214	Pseudo R2	= 0.0471

cooperation	Coefficient	Std. err.	z	P>|z|	[95% conf.	interval]
**v_bad**						
govmode3						
MIX	−.2962658	.5273258	−0.56	0.574	−1.329805	.7372738
BU	−1.612717	.4040795	−3.99	0.000	−2.404698	−.8207354
_cons	−.8023465	.3337751	−2.40	0.016	−1.456534	−.1481592
**bad**						
govmode3						
MIX	−.554336	.3564065	−1.56	0.120	−1.25288	.144208
BU	−1.681375	.2569068	−6.54	0.000	−2.184903	−1.177847
_cons	.743578	.2255575	3.30	0.001	.3014934	1.185663
**average**						
govmode3						
MIX	−.4646845	.339299	−1.37	0.171	−1.129698	.2003293
BU	−1.262283	.2405938	−5.25	0.000	−1.733838	−.7907282
_cons	.9501923	.2186689	4.35	0.000	.5216091	1.378775
**good**	(base outcome)					
**v_good**						
govmode3						
MIX	1.575536	.5710949	2.76	0.006	.4562109	2.694862
BU	1.67817	.4933015	3.40	0.001	.7113166	2.645023

_cons	-1.757858	.4842342	-3.63	0.000	-2.70694	-.8087763

Multinomial logistic regression — Number of obs = 303; LR chi2(4) = 21.31; Prob > chi2 = 0.0003; Pseudo R2 = 0.0247

Log likelihood = -420.863

cooperation	Coefficient	Std. err.	z	P>|z|	[95% conf.	interval]
v_bad						
2.MB	.1684187	.490813	0.34	0.731	-.7935572	1.130395
_cons	-1.752539	.300427	-5.83	0.000	-2.341365	-1.163713
bad						
2.MB	-.0896516	.2997508	-0.30	0.765	-.6771524	.4978493
_cons	-.2066142	.1724145	-1.20	0.231	-.5445406	.1313121
average	(base outcome)					
good						
2.MB	.4646845	.339299	1.37	0.171	-.2003293	1.129698
_cons	-.9501923	.2186689	-4.35	0.000	-1.378775	-.5216091
v_good						
2.MB	2.040221	.5375633	3.80	0.000	.986616	3.093826
_cons	-2.70805	.4618802	-5.86	0.000	-3.613319	-1.802782

.
. * Approval

Multinomial logistic regression — Number of obs = 982

```
                                                        LR chi2(8)    =  44.27
                                                        Prob > chi2   = 0.0000
Log likelihood = -1372.273                              Pseudo R2     = 0.0159

------------------------------------------------------------------------------
    approval | Coefficient  Std. err.      z    P>|z|     [95% conf. interval]
-------------+----------------------------------------------------------------
v_bad        |
    govmode3 |
        MIX  |   .4087929   .5286635     0.77   0.439    -.6273684    1.444954
         BU  |  -.2885123   .4042231    -0.71   0.475    -1.080775    .5037505
             |
       _cons |  -2.151762   .3341076    -6.44   0.000    -2.806601   -1.496923
-------------+----------------------------------------------------------------
bad          |
    govmode3 |
        MIX  |   .4997647   .3368078     1.48   0.138    -.1603665    1.159896
         BU  |  -.0128064   .2473031    -0.05   0.959    -.4975115    .4718987
             |
       _cons |   -1.05315   .2120407    -4.97   0.000    -1.468742   -.6375579
-------------+----------------------------------------------------------------
average      |  (base outcome)
-------------+----------------------------------------------------------------
good         |
    govmode3 |
        MIX  |   .5031036   .3091962     1.63   0.104    -.1029098    1.109117
         BU  |   .6719647   .2146886     3.13   0.002     .2511828    1.092747
             |
       _cons |  -.7907856   .1930516    -4.10   0.000     -1.16916   -.4124114
-------------+----------------------------------------------------------------
v_good       |
    govmode3 |
        MIX  |   1.681759   .4600303     3.66   0.000     .7801157    2.583401
         BU  |   1.757643   .3853175     4.56   0.000     1.002435    2.512852
             |
```

```
       _cons |  -2.374906   .3696321    -6.43   0.000    -3.099371    -1.65044
------------------------------------------------------------------------------


Multinomial logistic regression                         Number of obs =    293
                                                        LR chi2(4)    =  15.50
                                                        Prob > chi2   = 0.0038
Log likelihood = -403.1249                              Pseudo R2     = 0.0189

------------------------------------------------------------------------------
    approval | Coefficient  Std. err.      z    P>|z|     [95% conf. interval]
-------------+----------------------------------------------------------------
v_bad        |
        2.MB |   .4087929   .5286635     0.77   0.439    -.6273684    1.444954
       _cons |  -2.151762   .3341076    -6.44   0.000    -2.806601   -1.496923
-------------+----------------------------------------------------------------
bad          |
        2.MB |   .4997647   .3368078     1.48   0.138    -.1603665    1.159896
       _cons |   -1.05315   .2120407    -4.97   0.000    -1.468742   -.6375579
-------------+----------------------------------------------------------------
average      |  (base outcome)
-------------+----------------------------------------------------------------
good         |
        2.MB |   .5031036   .3091962     1.63   0.104    -.1029098    1.109117
       _cons |  -.7907857   .1930516    -4.10   0.000     -1.16916   -.4124114
-------------+----------------------------------------------------------------
v_good       |
        2.MB |   1.681759   .4600303     3.66   0.000     .7801157    2.583401
       _cons |  -2.374906   .3696321    -6.43   0.000    -3.099371    -1.65044
------------------------------------------------------------------------------

.
. * Participation

Multinomial logistic regression                         Number of obs =  1,048
```

LR chi2(8) = 107.89
Prob > chi2 = 0.0000
Log likelihood = -1466.0256 Pseudo R2 = 0.0355

participat~n	Coefficient	Std. err.	z	P>|z|	[95% conf.	interval]
v_bad						
govmode3						
MIX	-.740559	.4203458	-1.76	0.078	-1.564422	.0833035
BU	-.1265307	.2579993	-0.49	0.624	-.6322	.3791385
_cons	-.4168938	.2391862	-1.74	0.081	-.8856901	.0519025
bad						
govmode3						
MIX	-.1940153	.2834375	-0.68	0.494	-.7495426	.361512
BU	-1.464693	.2125482	-6.89	0.000	-1.881279	-1.048106
_cons	.6817185	.1849906	3.69	0.000	.3191436	1.044293
average	(base outcome)					
good						
govmode3						
MIX	.2288416	.4828218	0.47	0.636	-.7174718	1.175155
BU	.549253	.3535665	1.55	0.120	-.1437247	1.242231
_cons	-1.386294	.3370999	-4.11	0.000	-2.046998	-.7255906
v_good						
govmode3						
MIX	-.4643056	.7424949	-0.63	0.532	-1.919569	.9909577
BU	-.125545	.4705335	-0.27	0.790	-1.047774	.7966837

```
       _cons |   -1.99243   .4351941    -4.58   0.000    -2.845395   -1.139465
------------------------------------------------------------------------------

Multinomial logistic regression                         Number of obs =    294
                                                        LR chi2(4)    =   4.41
                                                        Prob > chi2   = 0.3534
Log likelihood = -372.59488                             Pseudo R2     = 0.0059

------------------------------------------------------------------------------
participat~n | Coefficient  Std. err.      z    P>|z|     [95% conf. interval]
-------------+----------------------------------------------------------------
v_bad        |
        2.MB |  -.5465431   .3929757    -1.39   0.164    -1.316761    .2236751
       _cons |  -1.098612   .2144225    -5.12   0.000    -1.518873   -.6783519
-------------+----------------------------------------------------------------
bad          |  (base outcome)
-------------+----------------------------------------------------------------
average      |
        2.MB |   .1940153   .2834375     0.68   0.494     -.361512    .7495426
       _cons |  -.6817185   .1849906    -3.69   0.000    -1.044293   -.3191436
-------------+----------------------------------------------------------------
good         |
        2.MB |   .4228568   .4591909     0.92   0.357    -.4771408    1.322855
       _cons |  -2.068013   .3200052    -6.46   0.000    -2.695212   -1.440814
-------------+----------------------------------------------------------------
v_good       |
        2.MB |  -.2702903   .7273501    -0.37   0.710     -1.69587     1.15529
       _cons |  -2.674149   .4220911    -6.34   0.000    -3.501432   -1.846865
------------------------------------------------------------------------------

.
. * Accountability

Multinomial logistic regression                         Number of obs =  1,059
                                                        LR chi2(8)    = 113.41
```

```
                                                        Prob > chi2   = 0.0000
Log likelihood = -1462.2042                             Pseudo R2     = 0.0373

------------------------------------------------------------------------------
accountabi~y | Coefficient  Std. err.      z    P>|z|     [95% conf. interval]
-------------+----------------------------------------------------------------
v_bad        |
    govmode3 |
        MIX  |   -.290717   .3124921    -0.93   0.352    -.9031902    .3217562
         BU  |   1.162619    .207653     5.60   0.000     .7556266    1.569611
             |
       _cons |  -.7591051   .1826624    -4.16   0.000    -1.117117   -.4010933
-------------+----------------------------------------------------------------
bad          |  (base outcome)
-------------+----------------------------------------------------------------
average      |
    govmode3 |
        MIX  |   .3593381   .2951718     1.22   0.223     -.219188    .9378642
         BU  |   1.066639   .2246399     4.75   0.000     .6263529    1.506925
             |
       _cons |  -.9879467   .1980145    -4.99   0.000    -1.376048   -.5998455
-------------+----------------------------------------------------------------
good         |
    govmode3 |
        MIX  |   .6720938   .6908726     0.97   0.331    -.6819917    2.026179
         BU  |   2.760269   .5245778     5.26   0.000     1.732115    3.788423
             |
       _cons |     -3.157   .5105275    -6.18   0.000    -4.157616   -2.156385
-------------+----------------------------------------------------------------
v_good       |
    govmode3 |
        MIX  |   1.142097   1.235842     0.92   0.355    -1.280108    3.564303
         BU  |   2.927992   1.022692     2.86   0.004     .9235531     4.93243
             |
       _cons |  -4.543295   1.005305    -4.52   0.000    -6.513657   -2.572933
```

```
------------------------------------------------------------------------------


Multinomial logistic regression                         Number of obs =    298
                                                        LR chi2(4)    =   5.11
                                                        Prob > chi2   = 0.2761
Log likelihood = -343.36739                             Pseudo R2     = 0.0074

------------------------------------------------------------------------------
accountabi~y | Coefficient  Std. err.      z    P>|z|     [95% conf. interval]
-------------+----------------------------------------------------------------
v_bad        |
        2.MB |   -.290717   .3124921    -0.93   0.352    -.9031902    .3217562
       _cons |  -.7591051   .1826624    -4.16   0.000    -1.117117   -.4010933
-------------+----------------------------------------------------------------
bad          |  (base outcome)
-------------+----------------------------------------------------------------
average      |
        2.MB |   .3593381   .2951718     1.22   0.223     -.219188    .9378642
       _cons |  -.9879467   .1980145    -4.99   0.000    -1.376048   -.5998455
-------------+----------------------------------------------------------------
good         |
        2.MB |   .6720938   .6908726     0.97   0.331    -.6819917    2.026179
       _cons |     -3.157   .5105275    -6.18   0.000    -4.157616   -2.156385
-------------+----------------------------------------------------------------
v_good       |
        2.MB |   1.142097   1.235842     0.92   0.355    -1.280108    3.564303
       _cons |  -4.543295   1.005305    -4.52   0.000    -6.513657   -2.572933
------------------------------------------------------------------------------

.
. * Formation

Multinomial logistic regression                         Number of obs =    987
                                                        LR chi2(8)    =  40.28
```

Prob > chi2 = 0.0000
Log likelihood = -1412.574 Pseudo R2 = 0.0141

formation	Coefficient	Std. err.	z	P>|z|	[95% conf.	interval]
v_bad						
govmode3						
MIX	.3695992	.4497698	0.82	0.411	-.5119334	1.251132
BU	.0433149	.3234654	0.13	0.893	-.5906657	.6772955
_cons	-1.780586	.2791104	-6.38	0.000	-2.327633	-1.23354
bad						
govmode3						
MIX	.5826924	.3015447	1.93	0.053	-.0083244	1.173709
BU	.4140944	.2147517	1.93	0.054	-.0068112	.8350001
_cons	-.7997569	.1903574	-4.20	0.000	-1.172851	-.4266632
average	(base outcome)					
good						
govmode3						
MIX	.6638387	.3835118	1.73	0.083	-.0878307	1.415508
BU	1.085332	.2723906	3.98	0.000	.5514557	1.619207
_cons	-1.544197	.2527203	-6.11	0.000	-2.03952	-1.048875
v_good						
govmode3						
MIX	.7750643	.6074204	1.28	0.202	-.4154578	1.965586
BU	1.664052	.4394753	3.79	0.000	.8026965	2.525408
_cons	-2.696877	.421785	-6.39	0.000	-3.52356	-1.870193

Multinomial logistic regression   Number of obs =   276
LR chi2(4)   =   5.89
Prob > chi2   = 0.2073
Log likelihood = -363.00386   Pseudo R2   = 0.0081

formation	Coefficient	Std. err.	z	P>|z|	[95% conf.	interval]
v_bad						
2.MB	.3695992	.4497698	0.82	0.411	-.5119334	1.251132
_cons	-1.780586	.2791104	-6.38	0.000	-2.327633	-1.23354
bad						
2.MB	.5826924	.3015447	1.93	0.053	-.0083244	1.173709
_cons	-.7997569	.1903574	-4.20	0.000	-1.172851	-.4266632
average	(base outcome)					
good						
2.MB	.6638387	.3835118	1.73	0.083	-.0878307	1.415508
_cons	-1.544197	.2527203	-6.11	0.000	-2.03952	-1.048875
v_good						
2.MB	.7750643	.6074204	1.28	0.202	-.4154578	1.965586
_cons	-2.696877	.421785	-6.39	0.000	-3.52356	-1.870193

.
. * Fundraising

Multinomial logistic regression   Number of obs = 1,060
LR chi2(8)   = 78.40
Prob > chi2   = 0.0000

Log likelihood = −1462.1983 Pseudo R2 = 0.0261

fundraise	Coefficient	Std. err.	z	P>|z|	[95% conf.	interval]
v_bad						
govmode3						
MIX	1.396051	1.236655	1.13	0.259	−1.02775	3.819851
BU	3.125832	1.017966	3.07	0.002	1.130655	5.12101
_cons	−4.634729	1.004843	−4.61	0.000	−6.604184	−2.665274
bad						
govmode3						
MIX	.4797598	.5042981	0.95	0.341	−.5086463	1.468166
BU	1.483242	.3546158	4.18	0.000	.7882079	2.178276
_cons	−2.332144	.3312231	−7.04	0.000	−2.981329	−1.682959
average	(base outcome)					
good						
govmode3						
MIX	.3351786	.27627	1.21	0.225	−.2063007	.8766579
BU	.9104676	.193852	4.70	0.000	.5305246	1.290411
_cons	−.6834853	.1701162	−4.02	0.000	−1.016907	−.3500637
v_good						
govmode3						
MIX	1.282722	.3751602	3.42	0.001	.5474214	2.018022
BU	1.695086	.304037	5.58	0.000	1.099185	2.290988
_cons	−1.995672	.2848461	−7.01	0.000	−2.55396	−1.437384

```
Multinomial logistic regression                         Number of obs =    302
                                                        LR chi2(4)    =  13.33
                                                        Prob > chi2   = 0.0098
Log likelihood = -349.98488                             Pseudo R2     = 0.0187

------------------------------------------------------------------------------
   fundraise | Coefficient  Std. err.      z    P>|z|     [95% conf. interval]
-------------+----------------------------------------------------------------
v_bad        |
        2.MB |   1.396051   1.236655     1.13   0.259     -1.02775    3.819851
       _cons |  -4.634729   1.004843    -4.61   0.000    -6.604184   -2.665274
-------------+----------------------------------------------------------------
bad          |
        2.MB |   .4797598   .5042981     0.95   0.341    -.5086463    1.468166
       _cons |  -2.332144   .3312231    -7.04   0.000    -2.981329   -1.682959
-------------+----------------------------------------------------------------
average      |  (base outcome)
-------------+----------------------------------------------------------------
good         |
        2.MB |   .3351786     .27627     1.21   0.225    -.2063007    .8766579
       _cons |  -.6834853   .1701162    -4.02   0.000    -1.016907   -.3500637
-------------+----------------------------------------------------------------
v_good       |
        2.MB |   1.282722   .3751602     3.42   0.001     .5474214    2.018022
       _cons |  -1.995672   .2848461    -7.01   0.000     -2.55396   -1.437384
------------------------------------------------------------------------------

----------------------------------------------------------------------------------------------------
------------------------------------------------------
```

End of Appendix 2
